# LIF, a mitogen for choroidal endothelial cells, protects the choriocapillaris: implications for prevention of geographic atrophy

**DOI:** 10.15252/emmm.202114511

**Published:** 2021-11-15

**Authors:** Pin Li, Qin Li, Nilima Biswas, Hong Xin, Tanja Diemer, Lixian Liu, Lorena Perez Gutierrez, Giovanni Paternostro, Carlo Piermarocchi, Sergii Domanskyi, Ruikang K Wang, Napoleone Ferrara

**Affiliations:** ^1^ Department of Pathology University of California San Diego La Jolla CA USA; ^2^ Department of Ophthalmology University of California San Diego La Jolla CA USA; ^3^ Sanford Burnham Prebys Medical Discovery Institute La Jolla CA USA; ^4^ Department of Physics and Astronomy Michigan State University East Lansing MI USA; ^5^ Department of Bioengineering University of Washington Seattle WA USA

**Keywords:** age‐related macular degeneration, cathepsins, endothelial diversity, IL‐6 family, STAT3, Vascular Biology & Angiogenesis

## Abstract

In the course of our studies aiming to discover vascular bed‐specific endothelial cell (EC) mitogens, we identified leukemia inhibitory factor (LIF) as a mitogen for bovine choroidal EC (BCE), although LIF has been mainly characterized as an EC growth inhibitor and an anti‐angiogenic molecule. LIF stimulated growth of BCE while it inhibited, as previously reported, bovine aortic EC (BAE) growth. The JAK‐STAT3 pathway mediated LIF actions in both BCE and BAE cells, but a caspase‐independent proapoptotic signal mediated by cathepsins was triggered in BAE but not in BCE. LIF administration directly promoted activation of STAT3 and increased blood vessel density in mouse eyes. LIF also had protective effects on the choriocapillaris in a model of oxidative retinal injury. Analysis of available single‐cell transcriptomic datasets shows strong expression of the specific LIF receptor in mouse and human choroidal EC. Our data suggest that LIF administration may be an innovative approach to prevent atrophy associated with AMD, through protection of the choriocapillaris.

## Graphical abstract

Little progress has been made in the therapy of the dry form of age‐related macular degeneration (AMD). We identified LIF as a novel mitogen and survival factor for choroidal EC. Protection of the choriocapillaris may be an innovative approach to prevent atrophy associated with AMD.

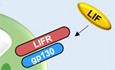

**The paper explained**

**Problem**

Major progress has been made in the prevention of blindness secondary to intraocular vascular diseases such as neovascular age‐related macular degeneration (AMD) through the use of VEGF inhibitors. However, dry AMD and geographic atrophy (GA) are major causes of vision loss in the elderly populations, and there are currently no effective treatments for these diseases. Loss of choriocapillaris (CC) has been shown to be an early event in AMD that precedes degeneration of retinal pigment epithelium (RPE). We hypothesized that strategies aiming to protect and/or regenerate the CC, potentially together with the RPE, may have therapeutic value for GA.

**Results**

Using a biochemical/functional approach, we sought to identify novel factors that may have protective effects on choroidal endothelial cells (CEC). We unexpectedly identified LIF, a member of the IL‐6 family, as a mitogen for cultured bovine CEC, although LIF has been previously characterized as an EC growth inhibitor. We show that LIF stimulation of the JAK‐STAT3 pathway mediates, depending on the EC type, both growth stimulation and growth inhibition. We also discovered that growth inhibition is mediated by STAT3‐induced cathepsin‐dependent apoptotic cell death and cell cycle arrest. Our data also show that LIF induces retinal and choroidal angiogenesis following intravitreal administration in the mouse. In addition, LIF and the related IL‐6 family member cardiotrophin‐1 (CT‐1) had protective effects on the CC in a model of oxidative injury, suggesting that these agents may help prevent the atrophy associated with AMD. Analysis of available single‐cell transcriptomic datasets shows high expression of the specific LIF receptor (LIFR) in human choroidal EC, comparable to the VEGF receptors.

**Impact**

This study advances our understanding of EC diversity and sheds light on EC‐type specificity of STAT3 signaling, potentially explaining some paradoxical results. Our data further suggest LIF administration as an innovative approach to prevent atrophy associated with AMD, through protection of the CC.

## Introduction

Angiogenesis is a major developmental and physiological process (Yancopoulos *et al*, 2000; Chung *et al*, 2010; Chung & Ferrara, 2011). The growth of new blood vessels is also a key aspect of a variety of pathological conditions, including tumors and intraocular vascular disorders (Adams & Alitalo, 2007; Chung & Ferrara, 2011). The newly formed vessels provide growing tumors with nutrients and oxygen and thus play an important role in tumor progression. Also, growth of abnormal and leaky blood vessels is associated with a variety of blinding ocular disorders (Adams & Alitalo, 2007; Chung & Ferrara, 2011; Potente *et al*, 2011). Over the last few decades, extensive efforts have been made to dissect the molecular basis of angiogenesis and to identify potential therapeutic targets. These efforts resulted in the discovery of the major signaling pathways involved in normal and abnormal vascular development (Adams & Alitalo, 2007; Chung & Ferrara, 2011; Potente *et al*, 2011). Much research has established the key role of the VEGF pathway in normal and pathological angiogenesis (Ferrara & Adamis, 2016; Apte *et al*, 2019). Indeed, current therapeutic approaches in angiogenesis rely on inhibiting the VEGF/VEGFR or Ang2/Tie2 pathways.

Conversely, promoting collateral vessel growth could provide a clinical benefit to patients with ischemic disorders, who have limited pharmacological options (Ferrara & Alitalo, 1999). This hypothesis led to numerous clinical trials in the past decades, testing a variety of angiogenic factors (e.g., VEGF‐A, VEGF‐C, HGF, FGF‐1, FGF‐2, and FGF‐4), in patients with coronary or limb ischemia (Isner, 1996; Carmeliet, 2005; Ferrara & Kerbel, 2005). However, these studies did not demonstrate therapeutic benefits, in spite of encouraging results in animal models (Simons, 2005). Thus, there is a need to further elucidate the molecular and biological basis of therapeutic angiogenesis.

Interestingly, earlier studies suggested the possibility of organ‐specific regulation of angiogenesis. Our laboratory described EG‐VEGF, a mitogen with a selectivity for endocrine gland EC (LeCouter *et al*, 2001). This finding raised the possibility that other factors with a selectivity for EC of specific vascular beds may exist. Indeed, several studies have emphasized the importance of organotypic properties of EC, e.g., the vascular bed‐specific release of growth factors and cytokines that can stimulate organ‐specific growth and regeneration (LeCouter *et al*, 2003; Red‐Horse *et al*, 2007; Rafii *et al*, 2016; Augustin & Koh, 2017).

Age‐related macular degeneration (AMD) is a leading cause of vision loss in Americans 50 years and older (Vingerling *et al*, 1995; Jager *et al*, 2008). Both genetic and environmental factors contribute to the pathogenesis of AMD (Jager *et al*, 2008; Zhang *et al*, 2012; Ambati *et al*, 2013; Yang *et al*, 2006). Early‐stage AMD is characterized by drusen and abnormalities of the retinal pigment epithelium (RPE). Late‐stage AMD can be neovascular (nv) (also known as wet or exudative) or non‐neovascular (known as atrophic or dry) (Ferris *et al*, 1984; Jager *et al*, 2008; Mitchell *et al*, 2018). While 10–15% of patients with intermediate AMD progress to nv form, the remaining patients who progress develop geographic atrophy (GA) (Jager *et al*, 2008; Fleckenstein *et al*, 2018). GA is characterized by severe visual impairment and scotomas due to loss of photoreceptors, RPE, and choriocapillaris (CC).

The last decade has witnessed significant progress in the prevention of blindness secondary to nv AMD (Ferrara & Adamis, 2016). Unfortunately, little progress has been possible in the therapy of dry AMD and GA. Loss of CC has been documented by histopathology and, more recently, through optical coherence tomography–angiography (OCT‐A) as an early event in AMD, and has been reported to occur underneath and beyond the areas of photoreceptor and RPE loss (Mullins *et al*, 2011b; Moreira‐Neto *et al*, 2018). Indeed, loss of CC has been reported to precede RPE degeneration (Curcio *et al*, 2000; Biesemeier *et al*, 2014). Recent studies provide evidence for deposition of membrane attack complexes in the choroid of patients with high‐risk *CFH* genotype (Mullins *et al*, 2011a). Therefore, loss of CC is strongly implicated in the pathogenesis of dry AMD and GA (Arya *et al*, 2018; Moreira‐Neto *et al*, 2018), raising the possibility that strategies aiming at protecting and/or regenerating the CC, together with the RPE, may be valuable. VEGF probably would not be suitable in this setting, given its vascular permeability‐enhancing properties (Apte *et al*, 2019).

Intriguingly, a recent exploratory analysis from the AREDS2 study has reported that enlargement of recent GA lesions slows down before the onset of nv AMD lesions, suggesting that non‐exudative nv tissue may prevent GA progression, likely due to perfusion improvement (Hwang *et al*, 2021).

In search of novel regulators of choroidal EC growth/survival, we screened human glioblastoma (GBM) cell lines since tumors have been historically proven to be a rich source of angiogenic factors (Kerbel, 2000). To identify such factors, we employed an unbiased proteomic/functional approach that relies on stimulating growth of primary bovine choroidal EC (BCE). We found that a particular GBM cell line (LN‐229) had unusual features that distinguished it from typical GBM, i.e., it had a very low VEGF expression (Depner *et al*, 2016). Intriguingly, the LN‐229‐conditioned medium had mitogenic effects on BCE, although none of the conventional EC mitogens was detectable. We identified LIF, a member of the IL‐6 family (Murakami *et al*, 2019), as the mitogen, although LIF has been previously characterized as an EC growth inhibitor. To understand the basis of such paradoxical effects, we analyzed signaling events elicited by LIF in BCE and bovine aortic EC (BAE), the EC type that was originally reported to be growth inhibited by LIF (Ferrara *et al*, 1992; Takashima & Klagsbrun, 1996). We also sought to establish whether LIF induces angiogenesis when injected in the eye and explored the possibility that LIF may have protective effects in models of injury to the CC, a structure that is critically affected in AMD.

## Results

### Identification of LIF as a mitogen for choroidal EC

**Figure 1:**
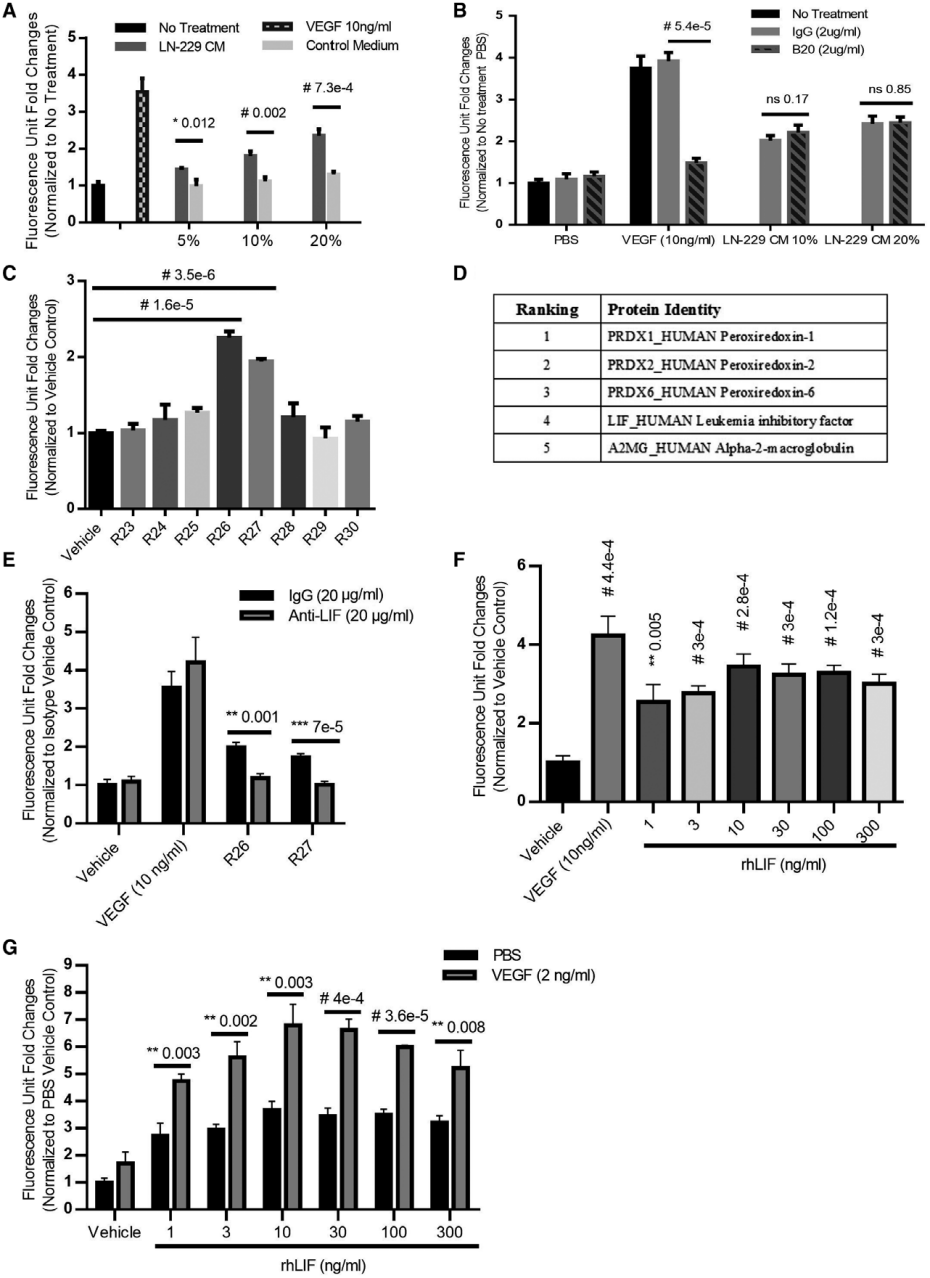
Identification of LIF as the EC mitogen in LN‐229‐conditioned medium (CM) LN‐229 CM stimulated growth of BCE cells, *n* = 3.VEGF neutralizing antibody (B20) failed to suppress BCE cell growth induced by LN‐229 CM, *n* = 3.Reverse‐phase fractions of LN‐229 CM induced BCE cell growth. BCE cells were incubated with fractions (2 μl/well) as indicated in the figure, *n* = 3.Candidate proteins generated from mass‐spectrometry analysis of LN‐229 CM reverse‐phase fractions. Candidates were identified by excluding intracellular proteins and proteins showing higher abundance in inactive fractions compared to those in mitogenic factions. Proteins were ranked for relative abundance as described in Materials and Methods.The anti‐LIF neutralizing antibody abolished BCE cell growth induced by reverse‐phase fractions, *n* = 3.Recombinant human LIF proteins stimulated growth of BCE cells in a dose‐dependent manner. BCE cells were cultured in the presence of vehicle, VEGF (10 ng/ml), and the indicated concentrations of recombinant human LIF (rhLIF, Sigma), *n* = 3.LIF and VEGF synergistically stimulated BCE cell growth. Cell proliferation was analyzed after 6 days using alamar blue as described in Materials and Methods, *n* = 3. Data information: Bars and error bars represent mean ± SD. All experiments were carried out in three independent studies. Two‐way ANOVA was used as statistical test. ns, not statistically significant. Source data are available online for this figure.

Media conditioned by LN‐229 cells (LN‐229 CM) stimulated growth of bovine choroidal EC (BCE) (Fig 1A). However, in agreement with previous studies (Depner *et al*, 2016), LN‐229 cells secreted very little VEGF in the medium (Appendix Fig S1A). The anti‐VEGF antibody B20‐4.1 did not suppress the mitogenic effects of LN‐229 CM (Fig 1B), suggesting the involvement of VEGF‐independent pathways. We examined the angiogenic factors profile of LN‐229 CM using specific antibody arrays. This analysis revealed that the majority of potential EC mitogens, including FGF‐1, FGF‐2, or HGF, were undetectable, except PDGF‐AA, CCL2 (also known as MCP‐1), and interleukin 8 (IL‐8), which were abundant in the CM (Appendix Fig S1B). However, antibodies neutralizing PDGF‐AA or CCL2 failed to suppress BCE cell growth induced by the LN‐229 CM (Appendix Fig S1C and D). In pilot experiments, we determined that IL‐8 lacks mitogenic effects on BCE cells.

To identify BCE mitogens in the LN‐229 CM, we undertook a biochemical/functional approach. The BCE mitogenic activity was enriched through two sequential chromatographic steps, anion‐exchange and reverse‐phase chromatography. At each step, only one peak of absorbance, composed of 4–5 contiguous fractions, showed mitogenic activity. The reverse‐phase column fractions were labeled as R1‐R45. The peak mitogenic fractions (R26 and R27), the minimally mitogenic (R25 and R28), and adjacent negative (R24 and R29) fractions (Fig 1C) were subjected to mass spectrometry analyses. A short list of five candidate proteins was generated by screening out intracellular proteins (Fig 1D). Four of the five proteins were serum components and/or redox enzymes (i.e., peroxiredoxins and alpha‐2‐macroglobulin), while LIF stood out as a cytokine. LIF, a member of the interleukin 6 (IL‐6) family of proteins (Murakami *et al*, 2019), is broadly expressed and exerts effects in multiple cell types and tissues, and has been implicated in various critical physiological processes including embryonic stem cell self‐renewal and blastocyst implantation (Nicola & Babon, 2015). The presence of LIF herein was unexpected, since this cytokine had been previously characterized as an EC growth inhibitor and an anti‐angiogenic agent (Pepper *et al*, 1995; Ash *et al*, 2005; Kubota *et al*, 2008). Nevertheless, an anti‐LIF polyclonal antibody completely suppressed growth of BCE cells induced by the reverse‐phase fractions (Fig 1E). These observations suggested that LIF might be responsible for the mitogenic effects. Indeed, recombinant LIF stimulated growth of BCE cells (Fig 1F) and bovine retinal EC (BRE) cells (Appendix Fig S2A). Interestingly, VEGF and LIF resulted in more than additive mitogenic effects in both BCE (Fig 1G) and BRE cells (Appendix Fig S2B), suggesting a synergistic relationship between the two factors. In addition, we tested the mitogenic effect of LIF on several human ECs. LIF stimulated the proliferation of human choroidal EC and human liver sinusoidal ECs to an extent comparable with VEGF (Appendix Fig S2C and D).

### Stimulation of BCE growth is mediated by the JAK‐STAT3 pathway

**Figure 2:**
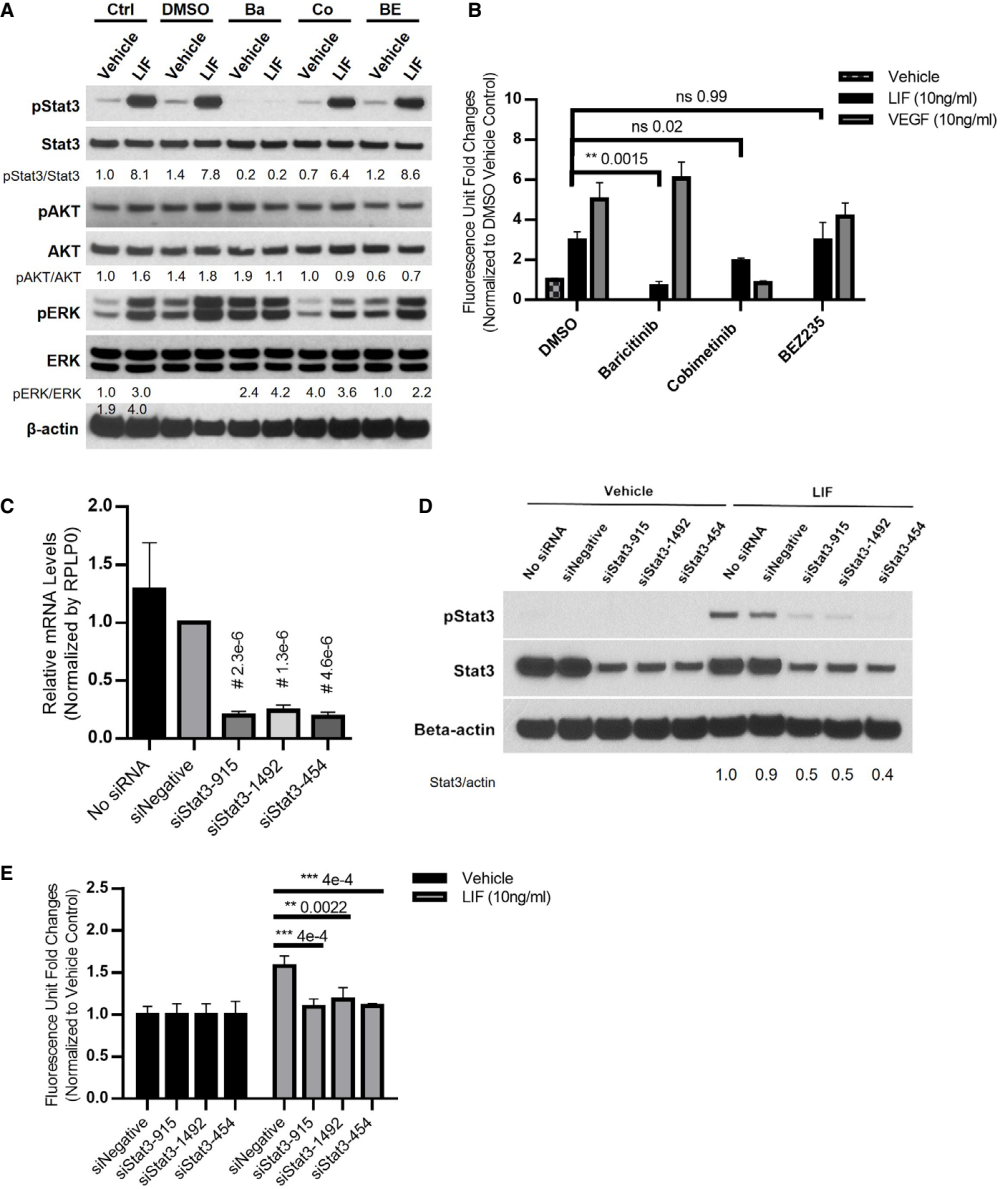
LIF promotes BCE cell growth via the JAK‐STAT3 pathway AThe JAK inhibitor baricitinib (Ba) blocked LIF‐induced STAT3 phosphorylation. BCE cells were preincubated with DMSO, baricitinib (2 μM), cobimetinib (Co) (150 nM), or BEZ235 (BE) (5 nM) for 1 h and were then treated with vehicle or LIF (10 ng/ml, Sigma) for 15 min. Ctrl, no preincubation with inhibitors.BBaricitinib suppressed LIF‐induced BCE cell growth. BCE cells were preincubated with DMSO, baricitinib, cobimetinib, or BEZ235 for 1 h and then treated with vehicle, LIF (10 ng/ml), or VEGF (10 ng/ml). Cell proliferation was analyzed after 6 days, *n* = 3.C, DSTAT3 knockdown in BCE cells. BCE cells were transfected with siNegative and siRNAs targeting STAT3. qRT‐PCR was performed to examine STAT3 mRNA levels. STAT3 level in siNegative was set as 1. Data from three independent experiments were averaged and are presented in C. In D, cells transfected with siRNAs were treated with LIF (10 ng/ml) or vehicle for 15 min. Whole‐cell lysates were subjected to Western blotting with the indicated antibodies.ELIF‐induced BCE cell growth was abolished by STAT3 knockdown. BCE cells with STAT3 knockdown were cultured with LIF (10 ng/ml, Sigma) or vehicle. Cell proliferation was analyzed after 3 days. Fluorescence reading at 590 nm for each vehicle group was set as 1, *n* = 3. siNegative, negative control siRNA not targeting any known genes. Data information: Bars and error bars represent mean ± SD. All experiments were carried out in three independent studies. Two‐way ANOVA was used as statistical test. Source data are available online for this figure.

Although all members of the IL‐6 family share a receptor component, gp130, LIF signaling transduces via the gp130:LIFR receptor dimer, while IL‐6 activates its downstream signal through the IL6Rα:gp130:gp130:IL6Rα tetramer (Nicola & Babon, 2015; Murakami *et al*, 2019). Among four Janus kinases (JAK1, JAK2, JAK3, and TYK2) associated with gp130, LIF signaling selectively activates JAK1 through transphosphorylation (Rodig *et al*, 1998; Nicola & Babon, 2015). Upon activation by LIF, JAKs elicit three distinct signaling cascades: JAK‐STAT, PI3K‐AKT‐mTOR, and RAS‐MAPK, which contribute to different functions in a cell‐type‐specific manner. As to JAK‐STAT pathway, LIF signaling preferentially activates STAT3, although STAT1 and STAT5 can also be phosphorylated by JAK1 (Kiu & Nicholson, 2012). To examine which pathways are responsible for LIF‐induced growth stimulation in BCE cells, we employed a set of small‐molecule inhibitors: baricitinib, cobimetinib, and BEZ235, which are specifically against JAK1/2, MEK1/2(MAPK pathway), and PI3K/mTOR, respectively (Serra *et al*, 2008; Liu *et al*, 2019; Tong *et al*, 2020). In BCE cells, LIF treatment for 15 min elicited phosphorylation of STAT3 and ERK but had little effect on AKT phosphorylation (Fig 2A). Preincubation with the JAK1/2 inhibitor baricitinib almost completely suppressed LIF‐induced STAT3 and ERK/MAPK phosphorylation (Fig 2A), while cobimetinib pretreatment blocked ERK phosphorylation but showed no effects on STAT3 and AKT phosphorylation (Fig 2A). BEZ235 had only moderate effects on AKT phosphorylation regardless of LIF treatment (Fig 2A). Moreover, baricitinib completely blocked LIF‐induced cell growth, while cobimetinib showed minimal effects and the PI3K/mTOR inhibitor BEZ235 had no effect on LIF‐stimulated cell growth (Fig 2B). These observations suggested that the MAPK and PI3K pathways might not be major contributors to LIF stimulation in BCE cells, and thus JAK‐STAT might be implicated. Since STAT3 is the preferential mediator in LIF‐induced JAK‐STAT signaling cascade (Kiu & Nicholson, 2012) and has been implicated in proliferation and survival in a wide variety of cell types, we further examined the role of STAT3 in BCE by siRNA knockdown. siRNAs successfully dampened STAT3 levels at both RNA and protein levels in BCE cells (Fig 2C and D). Downregulation of STAT3 blocked LIF‐induced BCE cell growth *in vitro* (Fig 2E). Moreover, LIFR siRNAs abolished the growth‐promoting effect of LIF in BCE cells, confirming that the proliferation was mediated via the LIF/LIFR pathway (Appendix Fig S3A and B).

### LIF inhibits BAE growth via the JAK‐STAT3 pathway

**Figure 3:**
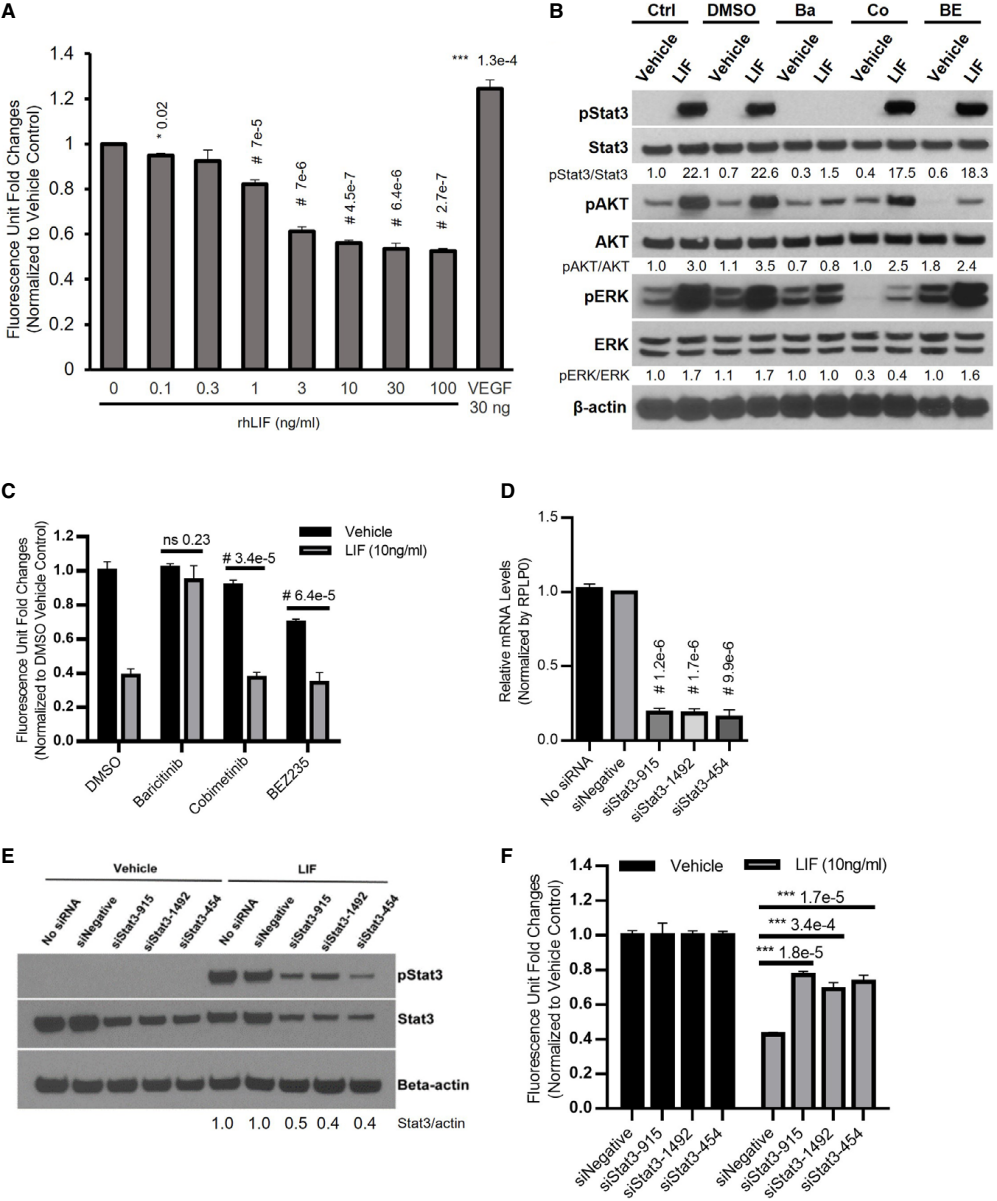
LIF inhibits BAE cell growth through the JAK‐STAT3 pathway ARecombinant human LIF inhibited growth of BAE cells in a dose‐dependent manner. BAE cells were cultured in the presence of vehicle and indicated concentrations of recombinant human LIF (rhLIF). Cell proliferation was analyzed after 6 days, *n* = 3.BJAK inhibitor baricitinib blocked activation of STAT3 by LIF. BAE cells preincubated with DMSO and inhibitors for 1 h were treated with vehicle and LIF (10 ng/ml) for 15 min. Whole‐cell lysates were subjected to Western blotting with indicated antibodies. Ctrl, no preincubation with inhibitors; Ba, baricitinib (2 μM); Co, cobimetinib (150 nM); BE, BEZ235 (5 nM).CThe JAK inhibitor baricitinib reversed LIF‐induced BAE growth inhibition. BAE cells preincubated with inhibitors for 1 h were treated with vehicle and LIF (10 ng/ml, Sigma). Cell proliferation was analyzed after 6 days using alamar blue, *n* = 3.D, EKnockdown of STAT3 in BAE cells. BAE cells were transfected with siRNAs targeting STAT3. qRT‐PCR was performed to examine STAT3 mRNA levels. STAT3 level in siNegative was set as 1. Data from three independent experiments were averaged and shown in D. In E, cells transfected with siRNAs were treated with LIF (10 ng/ml, Sigma) and vehicle for 15 min. Whole‐cell lysates were subjected to Western blotting with indicated antibodies.FLIF‐induced BAE cell growth inhibition was abolished by knockdown of STAT3. BAE cells with STAT3 knockdown were cultured with LIF (10 ng/ml, Sigma) and vehicle. Cell proliferation was analyzed after 3 days. Fluorescence reading for each vehicle group was set as 1, *n* = 3. Data information: Bars and error bars represent mean ± SD. All experiments were carried out in three independent studies. siNegative, negative control siRNA not targeting any known genes. Two‐way ANOVA was used as statistical test. Source data are available online for this figure.

In agreement with previous studies (Ferrara *et al*, 1992), LIF resulted in BAE cell growth inhibition (Fig 3A), To interrogate the LIF‐induced signaling cascade in BAE cells, we again used baricitinib, cobimetinib, and BEZ235 in order to inhibit LIF‐gp130:LIFR downstream components JAK1/2, MEK1/2, and PI3K/mTOR (Serra *et al*, 2008; Liu *et al*, 2019; Tong *et al*, 2020). In BAE cells, LIF treatment for 15 min led to phosphorylation of STAT3, ERK (MAPK), and AKT (Fig 3B). Baricitinib pretreatment significantly suppressed LIF‐induced phosphorylation of STAT3, ERK, and AKT, while cobimetinib and BEZ235 pretreatment also effectively repressed phosphorylation of ERK and AKT, respectively (Fig 3B). Remarkably, baricitinib was the only inhibitor that reversed growth suppression induced by LIF in BAE cells (Fig 3C), suggesting that the JAK‐STAT pathway mediated effects of LIF in BAE cells. To further examine whether inhibition of BAE cells by LIF was attributed to the JAK‐STAT3 cascade, STAT3 was knocked down by approximately 80% with three different siRNA in BAE cells (Fig 3D and E). Interestingly, knockdown of STAT3 in BAE cells reduced growth inhibition by LIF (Fig 3F). Consistent with these findings, LIFR knockdown by siRNA also abolished the inhibitory effects of LIF on BAE cells (Appendix Fig S3C and D). These findings further support the notion that LIF inhibited BAE growth via LIF/LIF receptor and JAK‐STAT3 pathway.

### LIF inhibits BAE growth via cathepsin L‐dependent cell death and cell cycle arrest

We next examined which growth inhibitory effects (e.g., cell cycle arrest, cellular senescence, or programmed cell death) were induced by LIF in BAE cells. Since IL‐6‐STAT3 signaling was tightly associated with cellular senescence (Kojima *et al*, 2013), we first hypothesized that LIF‐STAT3 axis also induced senescence in BAE cells. However, in the senescence‐associated β‐galactosidase assay, we did not observe increased numbers of senescent cells in BAE cell treated with LIF for 48 h (Appendix Fig S4A), suggesting that senescence was not the main effect elicited by LIF in BAE cells. Interestingly, staining for the cell death marker Annexin V showed an increased proportion of cells were Annexin V positive in BAE cells treated with LIF for 24 h (Appendix Fig S4B and C), indicating that LIF treatment induced cell death. Surprisingly, co‐incubation with the caspase inhibitors (Q‐VD‐OPH, Z‐VAD‐fmk, and Z‐DEVD‐fmk) or poly (adenosine 5'‐diphosphate ribose) polymerase (PARP) inhibitor (5‐AIQ) did not prevent cell death induced by LIF (Appendix Fig S4D), suggesting that a caspase‐independent pathway may be involved.

It has been previously reported that STAT3 can induce caspase‐independent cell death via upregulation of lysosomal proteases cathepsins L (CTSL) (Kreuzaler *et al*, 2011). Therefore, we examined whether LIF might elicit such a signaling cascade in BAE cells. Interestingly, CTSL was dramatically upregulated by LIF at both mRNA and protein levels by 24 h of treatment in the BAE cells, while CTSL mRNA levels in BCE cells were undetectable by qRT‐PCR, irrespective of whether the cells were incubated with vehicle or LIF (Appendix Fig S5A and B). Co‐incubation with CA074me, an inhibitor antagonizing both cathepsin B and L, alleviated LIF‐induced growth inhibition in BAE cells in a dose‐dependent manner of CA074me (Appendix Fig S5C and D). Moreover, another cathepsin L‐specific inhibitor, CAA0225, also repressed LIF‐induced growth inhibition in BAE cells, although by a lesser extent (Appendix Fig S5E). In contrast, the cathepsin B‐selective inhibitor CA074 was not able to suppress LIF‐induced effects in BAE cells even at the highest dose tested, 50 μM (Appendix Fig S5F). Interestingly, cathepsin L mRNA (CTSL) expression in BCE cells was undetectable by qRT‐PCR, irrespective of LIF administration. These data collectively indicated that LIF‐induced upregulation of cathepsin L in BAE cells, which in turn led to caspase‐independent cell death.

Moreover, after incubation with LIF for 48 h, BAE cells showed significantly reduced BrdU incorporation compared to the vehicle control (Appendix Fig S6A and B), suggesting that cell cycle arrest was elicited by LIF. This notion was supported by downregulation of cyclin A/B in LIF‐treated BAE but not in BCE cells (Appendix Fig S6C). This result confirmed that LIF induced opposite effects on endothelial due to cell‐type‐specific transcriptional programs.

In addition, LIF inhibited growth of human dermal microvascular ECs (Appendix Fig S7A). A biphasic effect on dermal ECs was observed, with a maximal inhibitory effect at 3–30 ng. At higher LIF concentrations, the inhibition was relieved. Although the mechanism of the biphasic effect remains to be determined, cathepsin L was upregulated at LIF concentrations resulting in growth inhibition. The association of growth inhibition with cathepsin upregulation is consistent with the findings in BAE cells (Appendix Fig S7B). No cathepsin mRNA upregulation was observed in the other human EC tested.

### Differential gene expression induced by LIF in BAE versus in BCE cells

To characterize the differential effects of LIF in BAE versus BCE cells, genes induced/suppressed by LIF were analyzed by RNA‐seq following incubation with LIF for 6 h. Remarkably, LIF treatment led to distinct gene expression patterns in these two cell types (Appendix Fig S8; Datasets [Link], [Link], [Link]). The GO results at baseline in BCE versus BAE cells (Dataset EV1) show a significant upregulation of angiogenesis pathways at baseline in BCE cells. In BCE cells after LIF we also see upregulations of angiogenesis pathaways, including the VEGF and JAK‐STAT Pathways (Dataset EV2). Specific genes upregulated in BCE cells after LIF addition included STAT3 and other well‐known angiogenesis genes (HIF1A, SOCS3, OSMR, TNFRSF1A, THBS2, EFNA1, PDGFC, MYC, JUNB, STC1, TNFSF18, CD14, VEGFC, and NTS) (Dataset EV3).

### LIF promotes retinal EC growth *in vivo*

As noted, we found that LIF can induce proliferation of choroidal and retinal EC *in vitro*. However, previous studies in LIF^‐/‐^ or LIF overexpressing mice concluded that LIF negatively affects vascular functions in developing eyes (Ash *et al*, 2005; Kubota *et al*, 2008). To resolve these apparent discrepancies, we directly examined the effects of LIF *in vivo*.

**Figure 4:**
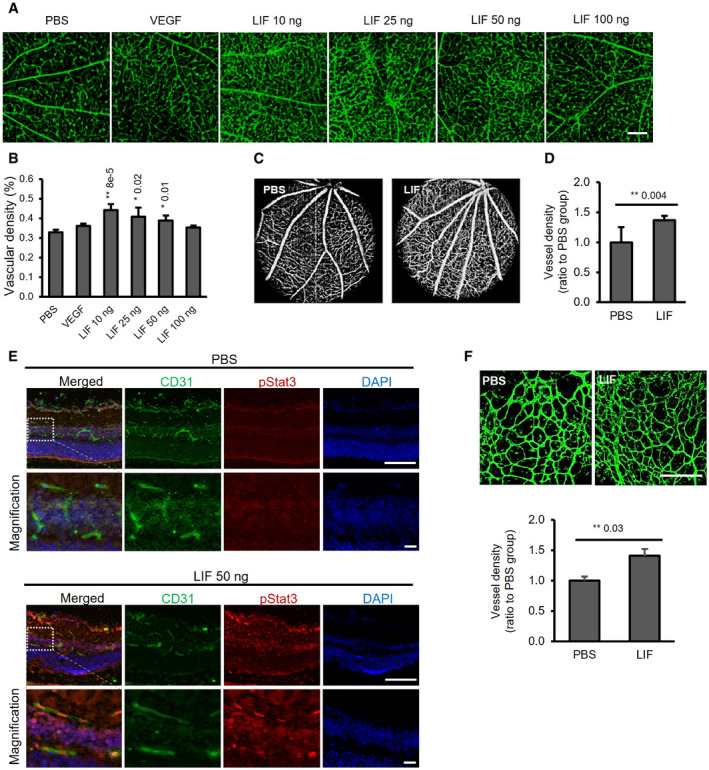
LIF promotes angiogenesis in *in vivo* models A, BIntravitreal injection of LIF increases blood vessel density in the mouse eyes. Adult mice were intravitreally injected with VEGF (10 ng) or LIF (10–100 ng, Sigma). Seven days after injection, PFA‐fixed choroid–sclera complexes and retina were subjected to CD31 IF. Representative images of CD31‐positive vessels are shown in A. Scale bar = 100 µm. Vascular density, determined with ImageJ software, is shown in B), *n* = 5–8.C, DOCT‐A imaging of LIF‐treated mouse retina. Adult mice were intravitreally injected with 1 μl of LIF (50 ng, Sigma) or vehicle solution (PBS). Retinal OCT‐A images were obtained 7 days after the injection and representative images are shown. Blood vessel density was determined as percentage of vessel‐covered area/total area surface using ImageJ software and shown in D), *n* = 7–8.ESTAT3 was phosphorylated in retinal ECs after LIF treatment. Fifty nanogram of our in‐house LIF with low endotoxin levels was injected. Phospho‐Stat3 was examined on cryosection of retina after 2 h by IHC. Magnification in the dashed box is shown in the lower panel. *n* = 5. Scale bar = 100 µm.FFive‐day‐old neonatal mice were intravitreally injected with LIF (50 ng) or vehicle solution (PBS). After 3 days, retinas were subjected to IF staining with Dyight‐488‐labeled lectin. Representative images for similar ocular loci and quantification of lectin‐labeled area using ImageJ software were shown, *n* = 4. Scale bar = 100 µm. Data information: Bars and error bars represent mean ± SEM. All experiments were carried out in three independent studies. Two‐way ANOVA was used as statistical test. Source data are available online for this figure.

Leukemia inhibitory factor, at the dose of 10 ng per eye, significantly increased retinal microvessel density, as assessed by both whole‐mount staining of retina with an antibody against EC surface marker CD31 (Fig 4A). Indeed, in several experiments, we found that 10–50 ng LIF consistently increased retinal microvessel density, while the dose of 100 ng was less effective (Fig 4A and B), consistent with the bell‐shaped responses observed for many cytokines (Atanasova & Whitty, 2012). OCT‐A imaging also documented significant increases in retinal vessel density following LIF injection (Fig 4C and D). Immunohistochemical (IHC) staining for CD31 also demonstrated that LIF injection increased vascular density in cross sections of adult mouse retina (Appendix Fig S9A). A time‐course study shows an increase in retinal vascular density even after 14 days, with a repeated LIF injection at day 7 (Appendix Fig S9B).

To further confirm that LIF can stimulate retinal ECs *in vivo*, cryosections of retinas from mice intravitreally injected with LIF or control were used to probe the phosphorylation level of STAT3 by IHC staining. Two hours after LIF injection, there was a clear STAT3 phosphorylation signal in ECs, as assessed by CD31 IHC (Fig 4E). The rapidity of the stimulation argues for a direct effect of LIF on ECs. Ongoing studies, employing conditional cell‐type‐specific *lifr* inactivation, should help determine whether paracrine mechanisms also play a role. Consistent with previous studies, activation of STAT3 was also detected in the retinal ganglion cell layer (Zhang *et al*, 2008a).

To characterize these newly formed vessels, a co‐staining of NG2, a pericyte marker, and CD31 was carried out. The results show that after LIF treatment, vessels were covered by pericytes to the same extent as the control group, suggesting these LIF‐induced retinal vessels were structurally normal (Appendix Fig S9C).

The difference between the aforementioned observations that LIF may negatively affect vascular development (Ash *et al*, 2005; Kubota *et al*, 2008) and our data led us to speculate that LIF possibly performs distinct roles in retinal angiogenesis at different developmental stages. Importantly, LIF also plays a critical role in retinal astrocyte maturation which can in turn affect development of retinal vasculature (West *et al*, 2005; Sakimoto *et al*, 2012). To examine the effects of LIF on the developing retinal vasculature and to minimize its impact on astrocyte development, LIF was intravitreally injected into 5‐day postnatal (P5) mice, a stage in which retinal vasculature is developing but the astrocyte network has already established and is undergoing maturation (Duan *et al*, 2017). We found that after LIF exposure for 3 days in neonatal mice, there was a significant increase in vascular density (Fig 4F).

Sakimoto and colleagues injected LIF intravitreally into P3 LIFKO or APLKO mice and observed a reduction in vascular density at P5 (Sakimoto *et al*, 2012). We injected multiple doses of LIF at P3 and measured vascular density at P5 and P8. In our hands, LIF neither promoted nor inhibited retinal angiogenesis at P5, although it induced an increase in retinal vascular density at P8 at the dose of 50 ng. (Appendix Fig S10B and D). The 500‐ng dose had no effect, consistent with a bell‐shaped dose response (Appendix Fig S10D). It is difficult, however, to compare our experiments in wild‐type mice with the above‐mentioned study, which tested LIF KO and APJ KO mice, reported to have abnormalities in the retinal vasculature (Sakimoto *et al*, 2012).

Interestingly, LIF, unlike VEGF, did not induce vascular permeability in the guinea pig skin (Appendix Fig S11A). In addition, we sought to determine whether LIF induced retinal microvascular leakage in mice using TRITC‐labeled dextran. LIF (100 ng), VEGF (100 ng), or combination of LIF and VEGF was injected intravitreally. TRITC‐dextran was used to indicate the leakage. Unlike VEGF, LIF did not induce vascular leakage or increased the leakage in LIF/VEGF combination group (Appendix Fig S11B).

We also tested the effects of two additional IL‐6 family members, cardiotrophin‐1 (CT‐1) (Pennica *et al*, 1995) and oncostatin M (OSM) (Gearing & Bruce, 1992), on retinal vascularization. Comparable to LIF, 20 and 100 ng CT‐1 resulted, respectively, in approximately 30 and 50% increases in retinal vascular density (Appendix Fig S12A and B). In contrast, OSM treatment decreased vascular density in the retina (Appendix Fig S12C and D). Interestingly, cathepsin B and L expression was upregulated in the eyes of OSM treated, but not in the LIF or CT‐1 treated, suggesting that the opposite effects of OSM and LIF (or CT‐1) are due to differential effects on cathepsin expression (Appendix Fig S12E).

To verify that the proangiogenic effects were truly induced by LIF rather than by trace amounts of contaminants such as LPS or by unspecific events related to the injection, in initial experiments recombinant LIF (Sigma) was heat inactivated by exposure to 95°C for 2 h, which does not affect endotoxin stability. Such treatment largely abolished LIF ability to promote angiogenesis *in vivo* (Appendix Fig S13A and B) and mitogenesis *in vitro* (Appendix Fig S13C). The LPS conc. in the Sigma LIF was reported to be < 0.1 EU/µg protein, a value that should be adequate, especially considering the low amounts of LIF injected in our experiments. Moreover, we expressed and purified recombinant LIF in our laboratory. The LPS level of this in‐house LIF was 0.02 EU/mg protein, a concentration > 100 lower than that in Sigma LIF. Therefore, this reagent should be fully suitable for *in vivo* studies (Aiello *et al*, 1995; Nowak‐Sliwinska *et al*, 2018). This LIF preparation promoted retinal angiogenesis, similar to the Sigma protein.

To further confirm that the increased retinal blood vessel density was mediated by LIF, an anti‐LIF Mab, D25 (Kim *et al*, 1992), was co‐injected with 50 ng LIF in the mouse vitreous. This Mab has been recently reported to inhibit pancreatic cancer progression in GEMMs (Shi *et al*, 2019; Wang *et al*, 2019a). We found that Mab D25 completely inhibited LIF‐induced BCE proliferation or BAE growth inhibition (Appendix Fig S14A and B). Ten µg of Mab D25 completely blocked the retinal angiogenesis induced by LIF, while 2 µg partially blocked the effect. The control Mab had no inhibitory effect (Appendix Fig S14C).

### LIF stimulates choroidal angiogenesis

**Figure 5:**
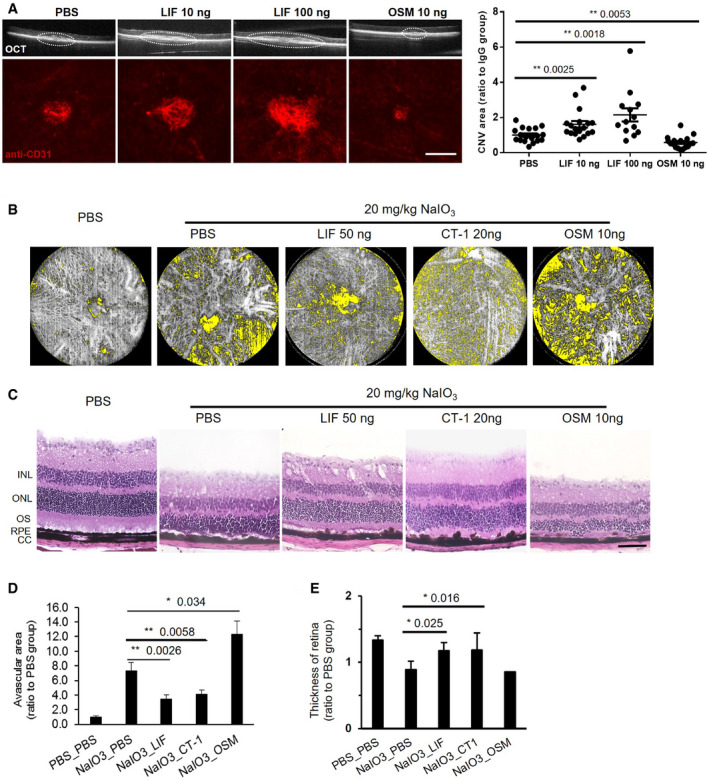
Effects of LIF and other IL‐6 family members in mouse ocular models IF enhanced laser‐induced CNV. Intravitreal administration of LIF (10 ng and 100 ng, Sigma) and OSM (10 ng) after laser‐induced CNV. PBS was used as vehicle control. OCT‐A imaging and immunostaining of choroid flat mounts were carried out 10 days after laser induction. Quantification of CNV area is shown. Dashed line circles indicate the CNV in OCT‐A images. *n* = 5. Dot plot shows all data points from three independent experiments. Scale bar = 100 µm.Sodium iodate was administered to induce retinal and CC damage in mice. After sodium iodate injection, the indicated amounts of LIF, CT‐1, or OSM were injected in the eyes. Choroid capillaries were imaged by OCT‐A system, *n* = 5. The avascular area in CC is indicated in yellow.H & E staining of eyes treated with different IL‐6 proteins following sodium iodate injection (*n* = 5). Representative images are shown. Scale bar = 100 µm.Avascular area in choroid in sodium iodate model was determined and quantified using Image J.Thickness of retina in H&E staining was quantified and bar graph was shown using ImageJ. Data information: Bars and error bars represent mean ± SEM. All experiments were carried out in three independent studies. Two‐way ANOVA was used as statistical test. Source data are available online for this figure.

Laser‐induced rupture of Bruch membrane is a commonly used technique to obtain CNV in different animal species, including the mouse (Ryan, 1979). The CNV model in the mouse was performed to test whether LIF further enhances choriocapillaris growth. LIF or OSM was intravitreally injected after laser induction. After 10 days, the OCT‐A imaging showed that the neovascularization area in LIF‐treated eyes was significantly larger than PBS group. Quantification of the lesion areas was performed after flat‐mount CD31 staining and showed that the CNV area was ˜60% larger after the treatment with 10 ng LIF and twice the size after treatment with 100 ng LIF. In contrast, OSM inhibited laser‐induced CNV, in agreement with the effects noted in the retinal vasculature (Fig 5A).

### Protective Effects of LIF in a model of atrophic AMD

The NaIO_3_ model in the mouse is a widely used preclinical model of atrophic AMD (Kannan & Hinton, 2014). In this model, both RPE layer and CC are heavily damaged by oxidative stress damage induced by NaIO_3_ (Mizota & Adachi‐Usami, 1997). Therefore, LIF, CT‐1, and OSM were tested for their ability to promote CC recovery in this model. After intravenous injection of NaIO_3_, LIF, CT‐1, or OSM was injected intravitreally. A novel OCT‐A imaging system, which is designed for live mice imaging of CC, was used to monitor CC atrophy. After sodium iodate injection, OCT‐A imaging clearly showed that CC was heavily damaged in PBS group. Avascular areas in CC were quantified to indicate the degree of injury. Consistent with their effects on the retinal vasculature, LIF and CT‐1 reduced avascular areas when compared to the PBS group. In contrast, avascular areas in OSM‐treated choroids were larger than in the PBS group (Fig 5B and D). In H&E staining, after sodium iodate treatment, the outer segment layer of retina was heavily degenerated causing a significant reduction in retinal thickness, which is consistent with previous studies (Mizota & Adachi‐Usami, 1997). LIF or CT‐1 treatment markedly rescued this injury and maintained the thickness and structure of retina, indicating a protective effect in this oxidative injury model (Fig 5C and E). Moreover, LIF could reduce the avascular area in CC from day 5 after NaIO_3_ injection and could last at least until day 12 (Appendix Fig S15A). In the NaIO_3_ model, LIF effects were dose dependent, from 10 ng and the effect was saturated at 50 ng (Appendix Fig S15B). Therefore, we used 50 ng in the subsequent experiments.

### LIF is expressed and secreted by pericytes but not by choroidal EC

**Figure 6:**
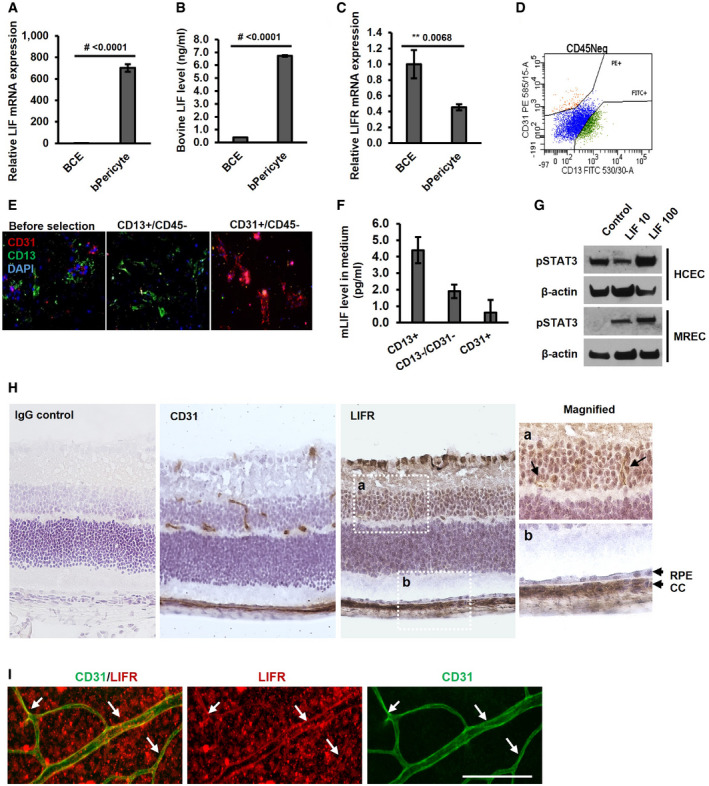
Expression of LIF and LIFR in ECs Q‐PCR (Taqman) analysis of LIF expression in BCE cells and bovine pericytes (bPericyte).Bovine LIF ELISA of BCE cells and bovine pericyte. Cells were cultured for 48 h before harvesting the medium.LIFR expression in BCE cells and bovine pericyte by qPCR (Taqman).Image of FACS selection of CD13^+^/CD45^−^ and CD31^+^/CD45^−^ cells.Immunostaining for CD31, CD13, and DAPI in isolated primary mouse cells before and after FACS selection.Mouse LIF expression in FACS‐selected cells.Human choroidal EC (HCEC) and mouse retinal EC (MREC) were treated with LIF 10 ng and LIF 100 ng for 30 min. (PBS was used as control). STAT3 phosphorylation was detected by Western blot.H&E staining of LIFR and CD31 in mouse eye sections. The magnified images of the dashed line boxes, a and b, are shown on the right panel. Box a shows LIFR staining in retina and box b showed LIFR staining in RPE/choroid layer.Whole‐mount staining of LIFR and CD31 in mouse retina. The fluorescent staining for CD31 (green) and LIFR (red) is shown. White arrows indicate the co‐staining of CD31 and LIFR on retinal ECs. Data information: Bars and error bars represent mean ± SEM. All experiments were carried out in three independent studies. Two‐way ANOVA was used as statistical test.

To confirm LIF and LIFR expression in primary EC and pericytes, Q‐PCR and ELISA assays were carried out. In primary human choroid EC and mouse retinal EC, LIF was barely detected by QPCR or bovine LIF ELISA, while LIFR resulted in a strong signal. Similarly, although LIFR was found in both primary bovine pericytes and BCEs, LIF was only detected in primary bovine pericytes (Fig 6A–C). To further confirm these findings, primary mouse choroid EC (CD31^+^/CD45^−^ cells) and pericytes (CD13^+^/CD45^−^ cells) were isolated from mouse choroid explants and selected by FACS (Fig 6D). Before and after selection, the identity of cells was confirmed by immunostaining with anti‐CD31 and anti‐CD13 antibodies (Fig 6E). The expression of LIF in the CD13^+^ group was over four times higher than that in CD31^+^ group and LIF was also detected in CD13^−^/CD31^−^ groups. These results indicate that LIF is mainly produced by pericytes (or other cell types), but not by EC (Fig 6F).

### Expression of LIFR in mouse retinal and choroidal cells by IHC

**Figure 7:**
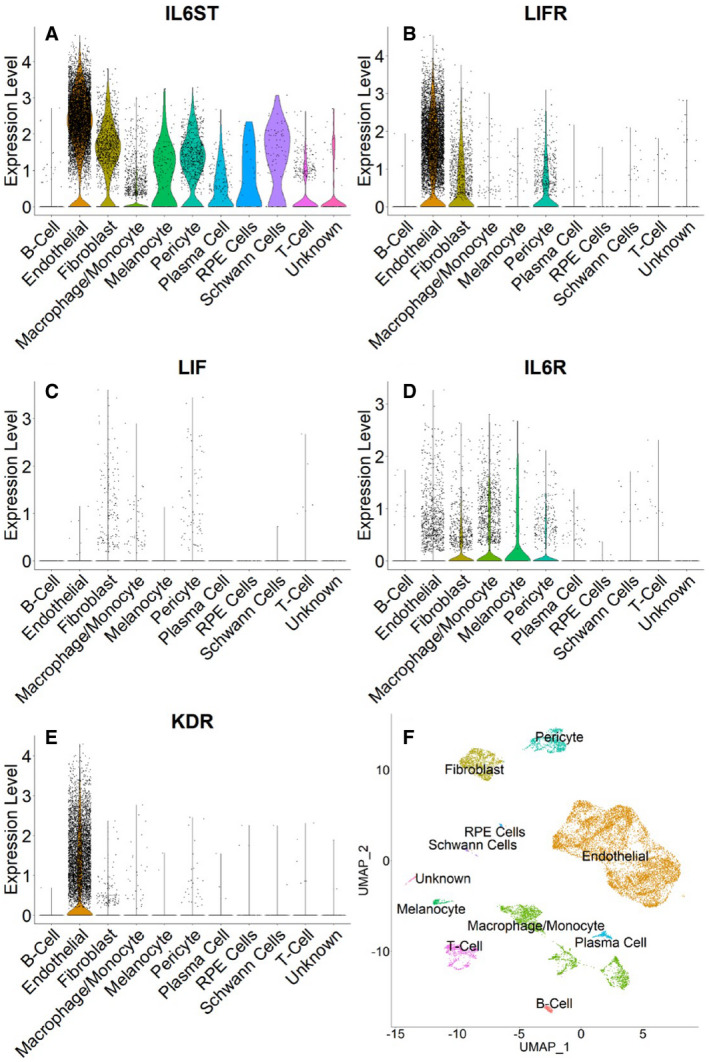
LIFR, but not LIF, is highly expressed in human choroidal ECs by single‐cell transcriptomic analysis A–EViolin plots of the log scale normalized expression of LIF receptor subunits, IL6ST (A), LIFR (B), LIF (C), IL6R (D), and KDR (E) in all cell types in the human choroid (based on Voigt *et al*, 2019). Note the wide distribution of IL6ST (gp130), the common subunit to all receptors of the IL‐6 family (A). In contrast, LIFR is strongly expressed in ECs and to a lesser extent in fibroblasts and pericytes (B). LIF is expressed in fibroblasts, macrophages/monocytes, and pericytes and minimally in ECs (C). The specific IL‐6 receptor (IL6R) is highly expressed in macrophage/monocytes (D), while LIFR has little expression in these cells (B). KDR shows, as expected, high expression selectively in EC (E).FCell clusters in *uniform manifold approximation and projection* (UMAP) space.

It has been reported that, at least in the neonatal mouse retina, LIF is produced by EC, while LIFR is expressed in surrounding cells such as astrocytes (Kubota *et al*, 2008). According to this hypothesis, EC‐derived LIF modulates the release of paracrine factors such as VEGF from glial and other cell types, which can in turn affect EC and other cell types (Kubota *et al*, 2008). On the other hand, our observation that LIF induces proliferation and STAT3 phosphorylation in cultured BCE (Fig 2) suggested that LIF could directly stimulate EC growth through binding to and activation of LIFR in EC. Treatment of human choroid EC and mouse retinal EC with LIF also resulted in STAT3 activation as assessed by a robust phosphorylation signal, indicating the presence of LIFR and IL6ST in human and mouse retinal EC (Fig 6G). These results suggest that LIF acts on choroidal EC via LIFR‐STAT3 signaling as a paracrine factor produced by pericytes and fibroblasts (see also Fig 7). To resolve this issue, we sought to determine whether LIF and its receptors are expressed in retinal or choroidal EC *in situ*. Immunohistochemical staining of LIFR in mouse eye sections shows that LIFR is highly expressed in the CC, while no significant expression was detected in the RPE layer (Fig 6H). LIFR was also expressed in multiple cell types in the retina. The expression pattern of LIFR in the retina presented a vessel‐like pattern, which was confirmed by CD31 staining (Fig 6H). Co‐immunofluorescent staining of LIFR and CD31 was carried out in mouse retinal flat mounts. Although LIFR was also expressed in other cell types, LIFR and CD31 co‐localized, suggesting the expression of LIFR in retinal vessels (Fig 6I). As already noted, 2 h after LIF injection, we detected a clear STAT3 phosphorylation signal in ECs in retinal sections (Fig 4E). The rapidity of the effects argues for a direct effect of LIF on ECs.

### Single‐cell transcriptomic analysis of LIF and LIFR expression

For human choroid, we clustered single‐cell transcriptome datasets (Voigt *et al*, 2019) using *Seurat* (Stuart & Satija, 2019; Wang *et al*, 2019b). Cell types were then identified and 8491 of the 14234 cells were classified as endothelial (Fig 7). The expression of genes of interest was then examined for each cell type (Fig 7 and Table EV1). LIF was expressed mainly in pericytes and fibroblasts (10% and 9% of cells, respectively), but had negligible expression in EC (.001%) (Fig 7C and Appendix Fig S16A). IL6ST (gp130) is a subunit shared by the receptors of the IL6 family, while the LIFR subunit is specific to the LIF receptor (Murakami *et al*, 2019). While IL6ST was expressed in several different cell types (Fig 7A), LIFR was mostly expressed in EC and, to a lesser extent, in fibroblasts and pericytes (Fig 7B, Table EV2). Notably, LIFR expression was minimal or absent in RPE cells. We also examined the expression of the specific IL6 receptor (IL6R). IL‐6 is the prototype member of the family and is a key mediator of inflammation (Murakami *et al*, 2019). LIF has pro‐inflammatory effects in some contexts (Kerr & Patterson, 2004) and anti‐inflammatory in others (Banner *et al*, 1998). Therefore, it was interesting to compare expression of IL6R and LIFR in inflammatory cells in the choroid. While IL6R was strongly expressed in cells of the monocyte/macrophage lineage, little LIFR expression was detected in these cells (Fig 7B). KDR (VEGFR2), as expected, showed high expression selectively in EC (Fig 7E).

Also, in a mouse choroidal EC dataset, *lif* was virtually undetectable, while *lifr* was strongly expressed, comparably to *vegfr2* (https://endotheliomics.shinyapps.io/murine_ectax/) (Rohlenova *et al*, 2020).

LIFR expression in retinal EC was detected in multiple mouse or human datasets (Macosko *et al*, 2015; Menon *et al*, 2019; Yan *et al*, 2020a, 2020b), although it was lower than in choroidal EC. LIF expression was much lower than LIFR in these retinal EC datasets.

To determine the expression of *lif* and *lifr* in EC from multiple mouse organs, we analyzed transcriptomic data from *Tabula muris* (Consortium, 2018). This dataset does not include ocular tissues. The analysis shows that *lifr* expression was highest in liver sinusoidal EC. However, EC from other organs had significant expression, at comparable levels (Appendix Fig S16B). *Lif* was rarely expressed.

We cultured human ECs which had been isolated from different organs and performed qPCR analysis to determine LIFR expression. The results show that, although the expression levels varied among different cell lines, they were in a relatively similar range (Appendix Fig S16C).

### Computational analysis of receptors in choroid EC

A set of receptors was identified measuring the differential expression of their directly connected downstream genes in EC versus other cell types, using single‐cell gene expression data from human choroidal cells (Voigt *et al*). These receptors were then clustered using the similarity of gene co‐expression within single cells. The cluster containing the main VEGF receptor (KDR) included 18 receptors, including the other major VEGF receptor (FLT1) and the two LIF receptor subunits, LIFR and IL6ST (gp130). This set of receptors was found to be significantly enriched for angiogenesis receptors (*P* < 0.0005). The results of this gene expression analysis are consistent with the experimental findings showing the role of LIFR in angiogenesis (Appendix Table S1).

The gene expression of LIFR in a large collection of single‐cell datasets of EC in different tissues was analyzed and LIFR expression was found to be very heterogeneous (Franzen *et al*, 2019) (Table EV2). For example, in addition to choroid EC, LIFR expression is high in EC from liver and testis but it is low in several brain areas.

In a separate computational study, we present an extensively validated method to identify a set of choroid endothelial receptors important in angiogenesis and this set again includes the VEGF and LIF receptors (preprint: Domanskyi *et al*, 2021).

## Discussion

We employed a proteomic/functional discovery strategy that relies on growth stimulation of BCE. Advantages of bovine EC are a stable phenotype for several passages and strong and reproducible proliferative responses to key EC growth factors such as FGF‐2 or VEGF. These properties enabled the original biochemical purification of these proteins (Shing *et al*, 1984; Ferrara & Henzel, 1989). The use of BCE also enabled seminal observations in eye research, such as identification of the role of RPE‐secreted VEGF on CEC growth (Sakamoto *et al*, 1995). Cultured human CE (HCE) would theoretically be more relevant, but they have been reported to exhibit little or no growth stimulation in response to VEGF, suggesting that they do not fully recapitulate key *in vivo* properties of HCE (Geisen *et al*, 2006; Peterson *et al*, 2007; Stewart *et al*, 2011).

Using our platform, we identified LIF as a mitogen for choroidal EC *in vitro*. The strong expression of LIFR in mouse and human choroidal EC by single‐cell transcriptomics and the angiogenic effects of intravitreally injected LIF validated this strategy. Recently, using a similar methodology, we discovered a novel pro‐angiogenic mechanism that relies on activating stress pathways (Zhong *et al*, 2020).

LIF was originally isolated and cloned in 1987 as an inducer of differentiation and an inhibitor of proliferation of leukemia cells (Gearing *et al*, 1987), but numerous, apparently unrelated, biological activities are known to be mediated by LIF (reviewed in (Nicola & Babon, 2015). LIF is a member of the IL‐6 cytokine family, which is comprised of 10 members: IL‐6, LIF, OSM, CT‐1, IL‐11, IL‐27, IL‐35, IL‐39, and cardiotrophin‐like cytokine factor 1 (CLCF1) (reviewed in Murakami *et al*, 2019).

In 1992, our laboratory identified LIF as a potent inhibitor of bovine aortic EC (BAE) growth (Ferrara *et al*, 1992). However, LIF had little inhibitory effects on adrenal capillary ECs, suggesting EC‐type‐specific effects. Subsequent reports described LIF as an inhibitor of FGF‐2‐ and VEGF‐induced EC proliferation (Pepper *et al*, 1995; Takashima & Klagsbrun, 1996). Indeed, prior to our present study, LIF has been described primarily as a negative regulator of EC growth/angiogenesis, although some studies have shown mitogenic effects of LIF in immortalized EC lines generated through SV40 large T antigen (Vasse *et al*, 1999). It is conceivable that differences in experimental model systems, dosing regimens, and perhaps also in the quality/purity of the reagents tested (e.g., LPS levels) explain at least some of the conflicting findings.

Decades after our initial report (Ferrara *et al*, 1992), we surprisingly identified LIF as a mitogen for BCE, emphasizing the biological versatility of this protein. We hypothesized that dissecting the signal transduction pathways elicited by LIF in both BCE and BAE cells would provide a valuable model system to identify some key mechanisms that might explain the diversity of LIF actions in EC and may also inform the investigation of HCE and other EC cell types.

We were able to confirm the earlier observation that LIF results in BAE cell growth inhibition (Ferrara *et al*, 1992). We show here that this could be largely attributed to cell death as evidenced by increase in Annexin V staining upon LIF treatment. Interestingly, two inhibitors of lysosomal cysteine protease cathepsin L, but not caspase inhibitors, reversed LIF‐induced cell death, providing evidence for caspase‐independent cell death. Moreover, the cathepsin B‐specific inhibitor CA074 failed to rescue BAE cell death, and cathepsin L but not cathepsin B was upregulated in LIF‐treated BAE cells, indicating that cathepsin L is the executer of LIF‐induced lysosomal cell death. In contrast, BCE did not show any induction of cathepsins by LIF. Interestingly, among four human ECs we tested, LIF inhibited the growth of dermal microvascular ECs. This effect was associated with upregulation of Cathepsin L by LIF treatment (Appendix Fig S7).

Induction of cathepsins B and L has been implicated in autophagy and cell death (Kaasik *et al*, 2005; Pensa *et al*, 2014). To our knowledge, this is the first report to implicate the LIF‐cathepsin L pathway in induction of EC death. Interestingly, both LIF and cathepsin L have been implicated in the development and progression of vascular diseases such as abdominal aortic aneurysm and atherosclerosis (Gillett *et al*, 1993; Liu *et al*, 2006; Li *et al*, 2009). These data broadly suggest a potential role of the LIF‐cathepsin L pathway in regulating the vasculature in pathological settings. LIF also led to reduced BrdU incorporation (Appendix Fig S4), accompanied by decrease in cyclin A/B expression, in BAE cells, indicating that LIF‐induced cell cycle arrest might play a role in BAE growth inhibition. It has been reported that cyclin A1 and cyclin B1 are direct STAT3 targets (Snyder *et al*, 2008). Also, STAT3 has been implicated in both upregulation and downregulation of cyclin A/B depending on specific settings (Zhou *et al*, 2017; Robinson *et al*, 2019), and suppression of cyclin A expression by STAT3 was mediated by its direct target PIM1 (Jin *et al*, 2014). This might explain why LIF repressed expression of cyclin A/B in BAE cells but not in BCE cells, since induction of PIM1 by LIF was observed only in BAE cells (Appendix Fig S6E).

An unexpected finding of our study is the opposite responses elicited by the same signaling pathway in two EC types, suggesting that activated STAT3 transactivates distinct sets of genes in these two cell types. Indeed, we found differential expression of some genes following LIF treatment in BCE and BAE cells, including downregulation of S phase and G2/M cyclin genes CCNA2 and CCNB1 as well as upregulation of lysosomal cysteine protease CTSL in BAE cells but upregulation of proliferative gene MYC only in BCE cells. Moreover, among the human EC we tested, expression of CTSL was upregulated only in dermal microvascular EC at LIF concentrations that resulted in growth inhibition (Appendix Fig S6E). In addition, in agreement with the opposite effect of LIF and OSM on retinal angiogenesis, only OSM upregulated CTSL expression following injection in the mouse eye (Appendix Fig S12E). Taken together, these observations suggest that CTSL expression could be a key biomarker to determine whether LIF (or other IL‐6 family member) has detrimental effects and thus help select patients and doses.

These observations may reflect the fact that different EC types have their unique gene expression pattern/epigenetic profiling, which determines their differential responses to the same stimulus (LeCouter *et al*, 2003; Nolan *et al*, 2013; Augustin & Koh, 2017). Our discovery of opposite effects of LIF in different EC exemplifies a novel aspect of such diversity: The same signaling pathway may mediate divergent effects, depending on EC‐type‐specific transcriptional programs.

Our *in vivo* studies suggest that LIF can directly stimulate ECs via STAT3 activation (Fig 4E). We also tested the hypothesis that recruitment of inflammatory cells such as macrophages may contribute to LIF effects by paracrine mechanisms. However, after LIF intravitreal injection, the number of macrophages in the retina was not increased relative to control (Appendix Fig S17A). We also performed *in vitro* studies on isolated mouse peritoneal macrophages. After incubation with LIF, we measured the expression levels of several pro‐angiogenic factors: VEGF, PDGF, bFGF, PlGF, TGFβ, angiopoietin2, and LIF itself. However, only PlGF was slightly upregulated by LIF (10 ng) (1.9‐fold) (Appendix Fig S17B).

Interestingly, CT‐1 also induced retinal angiogenesis and was also protective in the NaIO_3_ model. However, OSM had opposite effects, suggesting a specificity in such actions. Our initial evidence points to induction of cathepsin L by OSM, but not by LIF or CT‐1, in the retina as a possible mechanism for such differential effects.

According to some reports, LIF expression levels are negatively correlated with retinal vascular development in some genetically engineered mouse models (GEMMs) (Ash *et al*, 2005; Kubota *et al*, 2008). However, the constitutive nature of these mutations makes it difficult to dissect the direct effects of LIF, considering that this cytokine can affect multiple cell types in the eye at various stages of development and mediate a variety of paracrine interactions that could affect the vasculature in multiple ways (Ash *et al*, 2005; West *et al*, 2005; Kubota *et al*, 2008).

It has been reported that, at least in the neonatal mouse retina, LIF is produced mainly by EC, while LIFR is expressed in surrounding cells such as astrocytes (Kubota *et al*, 2008). However, recent single‐cell RNA sequencing studies have shown that, in several mouse organs examined, LIF is predominantly expressed in stromal cells including pericytes and fibroblasts and rarely in EC, while LIFR is frequently expressed in vascular EC (Appendix Fig S16B) (Consortium, 2018; Rohlenova *et al*, 2020). Importantly, analysis of single‐cell transcriptome data from human choroidal blood vessels (Voigt *et al*, 2019) is consistent with these conclusions (Fig 7B). In fact, in CD31‐enriched RPE‐choroid dataset, the highest LIFR expression was in EC and was comparable to the VEGF receptors or Tie2 (https://singlecell‐eye.com) (Table EV2 and Fig 7). An unexpected finding from the same choroidal datasets was the minimal LIFR expression in RPE cells, especially when compared to expression in EC (Fig 7B). This is in apparent contrast with previous *in vitro* and *in vivo* studies (Li *et al*, 2011; Chucair‐Elliott *et al*, 2012), suggesting that the RPE layer is a major LIF target. However, this finding is consistent with our IHC data in the mouse showing little or no LIFR immunostaining in the RPE (Fig 6H). Nevertheless, even low‐abundance transcriptomic signals may be associated with significant biological effects and further studies are required to address this issue.

Interestingly, LIFR expression in retinal EC was detected in multiple datasets (Macosko *et al*, 2015; Menon *et al*, 2019; Yan *et al*, 2020a, 2020b), although it was lower than in choroid EC. In this context, particularly high LIFR expression is detected not only in the choroid but also in choroid plexus and liver. In all of these cases, the vasculature is fenestrated, suggesting a vascular bed‐specific expression. Nevertheless, it is important to emphasize that not all EC express LIFR, at least as assessed by an analysis of a series of single‐cell transcriptome studies (Franzen *et al*, 2019), (Table EV1).

In a recent computational study (preprint: Domanskyi *et al*, 2021) we show, using a method validated independently in a large number of single‐cell gene expression datasets, that the VEGF receptor KDR, LIFR and IL6ST (gp130) are part of a naturally co‐occurring and evolutionary conserved set of human choroid EC receptors. This finding supports the results reported here and provides additional information about other receptors.

As already noted, LIF has complex effects on multiple cell types (Nicola & Babon, 2015), making difficult to identify a function of this cytokine that might represent a therapeutic target or application. However, several recent studies have described unique roles of LIF, clearly distinct from other family members such as IL‐6. For example, unexpected lung protective effects of LIF in the course of bacterial or viral infections have been described (Quinton *et al*, 2012; Foronjy *et al*, 2014). Also, two recent studies have implicated LIF in the progression of pancreatic cancer (Shi *et al*, 2019; Wang *et al*, 2019a).

The finding that LIF and CT‐1 have protective effects in the NaIO_3_ model raises the possibility that they may be of value for protecting the CC and thus for preventing atrophy in AMD, potentially in combination with other approaches that are currently pursued (Kerur *et al*, 2018; Kim *et al*, 2019; Wright *et al*, 2020). The lack of direct permeabilizing effects of LIF may prove particularly useful in this respect.

A key question for a potential therapeutic application is how to deliver to the eye LIF, a cytokine that has a short half‐life, at least in the systemic circulation (Stewart *et al*, 1992). It is reassuring that new technologies and delivery systems exist that enable long‐term delivery of proteins in the eye. One example is the refillable port delivery system for ranibizumab that provided steady release for at least 6 months and enabled a substantial reduction in the number of injections needed to maintain visual acuity (Campochiaro *et al*, 2019). Also, another member of the IL‐6 family, CNTF, was tested in GA patients, using encapsulated transfected cells as a delivery system. A trend toward improved vision was noted, although the progression of GA was not slowed down (Zhang *et al*, 2011). Both LIF and CNTF have neurotrophic effects that should be beneficial to AMD patients. However, CNTFR is not expressed in choroidal ECs based on analysis of mouse (Rohlenova *et al*, 2020) and human (Voigt *et al*, 2019) transcriptomic datasets. We hypothesize that the strong expression of LIFR in choroidal EC and the protective effects of LIF on the CC may be advantageous in this setting.

## Materials and Methods

### Reagents

Antibodies: anti‐human LIF polyclonal antibody was from Sigma (CAT# L9277). Normal goat IgG isotype control was from R&D Systems (CAT# AB‐108‐C). Anti‐LIF monoclonal antibody D25 (Kim *et al*, 1992) and mouse IgG2A isotype control were from Genentech.

Small‐molecule inhibitors: Baricitinib (Apexbio Technology, CAT# A414150), cobimetinib (MedChemExpress, CAT# HY‐13064), BEZ235 (Selleckchem, CAT# S1409), Z‐VAD‐FMK (R&D Systems, CAT# FMK001), Z‐DEVD‐FMK (R&D Systems, CAT# FMK004), Q‐VD(OMe)‐OPh (Apexbio Technology, CAT# A8165), 5‐AIQ hydrochloride (Sigma, CAT# A7479), CA‐074 me (Calbiochem, CAT # 205531), CA‐074 (Tocris, CAT # 4863), and CAA0225 (Calbiochem, CAT# 219502).

Recombinant Proteins: human LIF (Sigma, CAT# SRP9001), human LIF (Biolegend, CAT# 593902), human PDGF‐AA (Peprotech, CAT# 100‐13A), human Peroxiredoxin 1 (Abcam, CAT# ab74172), human IL‐8 (Biolegend, CAT# 574202), and human VEGF 165 (R&D Systems, CAT# 293‐VE).

### Cell culture

LN‐229 human glioblastoma cells (ATCC, CRL‐2611) were maintained in high‐glucose DMEM supplemented with 5% FBS. Bovine choroidal EC (BCE) (VEC Technologies, Rensselaer, NY, Cat# BCME‐4) (P5‐P9) and bovine retinal EC (BRE) (VEC Technologies, Rensselaer, NY, Cat# BRME‐3) (P5‐P9) cells were maintained in low‐glucose DMEM supplemented with 10% bovine calf serum (BCS), 2 mM glutamine, 5 ng/ml bFGF, and 10 ng/ml VEGF on fibronectin‐coated culture plates. Bovine aortic endothelial (BAE) cells (Cell Applications Inc. San Diego, CA, Cat# B304‐05, lot#1190) (P5‐P10) were maintained in DMEM–low glucose supplemented with 10% BCS. Human choroidal ECs (HCECs) (Celprogen, Torrance, CA, Cat#36052‐03) were maintained in choroid endothelial growth medium (Celprogen, M36052‐03S) with antibiotics in gelatin‐coated culture plates. Human lung microvascular ECs (Lonza, CC‐2527), human dermal microvascular ECs (CC‐2543), and human liver sinusoidal ECs (ACBRI 566) were cultured in EGM2‐MV medium (Lonza, CC‐3202). All cells were maintained at 37°C in a humidified atmosphere with 5% CO_2_. All cells employed in the present study were authenticated with specific markers and were mycoplasma free.

### EC proliferation assays

Bovine microvascular EC proliferation assays were performed essentially as previously described (Yu *et al*, 2008; Xin *et al*, 2016; Zhong *et al*, 2020). BCE (1 × 10^3^ cells/well) or BRE (5 × 10^2^ cells/well) cells were seeded in 96‐well plates in culture medium (DMEM–low glucose supplemented with 10% BCS, 2 mM glutamine, and antibiotics) plus testing materials BAE cells were plated in 96‐well plates at a density of 2 × 10^3^ cells. Given the higher proliferation rate of BAE compared to other EC types, BAE proliferation assays were done in DMEM–low glucose supplemented with 1% BCS, 2 mM glutamine, and antibiotics. Human ECs were seeded at a density of 1 × 10^3^ cells/well in gelatin‐coated 96‐well plates in assay medium (EBM‐2 supplemented with antibiotics) plus testing materials. The total assay volume was 200 μl per well. For assays involving antibodies or small‐molecule inhibitors, inhibitors or vehicle controls were first added and then test materials were added 1 h later. After 6 days (unless otherwise specified), cells were incubated with alamar blue for 4 h. Fluorescence was measured at 530 nm excitation wavelength and 590 nm emission wavelength. Each experiment was carried out in duplicate/triplicate and repeated at least three times.

### Preparation of LN‐229 cell conditioned medium

5 × 10^6^ LN‐229 cells were seeded in a 15‐cm culture dish with 35 ml of culture medium (DMEM–high glucose with 0.5% FBS and 1% antibiotics) and incubated at 37°C for 72 h. The LN‐229 CM were collected by centrifuging, filtered with a 0.22 μm filter, and stored at −80°C for later use.

### Chromatographic enrichment of EC mitogens in LN‐229 CM

Approximately 400 ml of LN‐229 CM were concentrated and then subjected to enrichment by two sequential chromatographic steps. CM was buffer exchanged to 20 mM Tris, pH 8.0, filtered (0.2 µm), and loaded to a 5‐ml HI‐Trap Q™ HP column (GE Healthcare, Pittsburgh, PA) using an AKTA Explorer System (GE Healthcare). After a stepwise elution with 0.2, 0.5, 1, and 2 M NaCl in the Tris buffer, aliquots of eluted fractions were tested in the BCE growth assay as described above. The mitogenic fractions were then pooled, diluted in 0.1% trifluoroacetic acid/H2O (TFA, ThermoFisher), and applied to a Synchropak RP C4 reverse‐phase column (4.6 × 100 mm, Eichrom Technologies, Darien, IL). The column was eluted with a linear gradient of acetonitrile/0.1% TFA. The fractions were evaporated, using a MiVac DUO Concentrator (Genevac, Ipswich, UK), washed, resuspended in PBS, and tested as above. The mitogenic fractions and adjacent negative ones were subjected to mass spectrometry analysis.

### ELISA

VEGF levels in LN‐229 CM samples were determined by a human VEGF ELISA kit (R&D Systems, CAT# DVE00) according to manufacturers’ instructions. Cathepsin L levels in BAE cells were measured using a bovine cathepsin L ELISA kit (MyBioSource, Inc, CAT # MBS2887609) per manufacturer’s instructions. Mouse and bovine LIF concentrations were measured using specific ELISA kits (R&D Systems, Cat# MLF00; MyBioSource, Inc, Cag# MBS766054). Cells were cultured for 48 h before harvesting the medium. 0.01% Tween was added before ELISA.

### Mass spectrometry analysis

Trypsin‐digested peptides were analyzed by ultra‐high pressure liquid chromatography (UPLC) coupled with tandem mass spectroscopy (LC‐MS/MS) using nanospray ionization. The nanospray ionization was performed using a Orbitrap Fusion Lumos hybrid mass spectrometer (ThermoFisher Scientific) interfaced with a nanoscale reversed‐phase UPLC Dionex UltiMate™ 3000 RSLC Nano System (ThermoFisher Scientific) using a 25 cm, 75‐µm ID glass capillary packed with 1.7‐µm C18 (130) BEHTM beads (Waters corporation). Peptides were eluted from the C18 column into the mass spectrometer using a linear gradient (5–80%) of acetonitrile. Mass spectrometer parameters are as follows: an MS1 survey scan using the orbitrap detector [mass range (*m/z*): 400–1,500 (using quadrupole isolation), 120,000 resolution setting, spray voltage of 2,200 V, ion transfer tube temperature of 275 C, AGC target of 400,000, and maximum injection time of 50 ms] was followed by data‐dependent scans (top speed for most intense ions, with charge state set to only include +2–5 ions, and 5 s exclusion time, while selecting ions with minimal intensities of 50.000 at in which the collision event was carried out in the high energy collision cell [HCD Collision Energy of 30%]), and the fragment masses were analyzed in the ion trap mass analyzer. Protein identification was carried out using PMI‐Byonic (PMI) version 2.9.30. Perl scripts based on the calculations described by Paoletti *et al* (2006). A Second Perl script was used to align all data from all samples into a single table. A list of five candidate proteins was established according to the following criteria: (i). the candidates should be extracellular or secretory proteins; (ii). the candidate proteins should be present in the active fractions but not detected in the negative fractions; and (iii) if also present in the negative fractions, the abundance of such proteins in negative fractions should be lower than that of the active fractions and the abundance peaked in the most active fractions.

### Senescence β‐galactosidase assay

BAE cells were plated onto six‐well culture plates at a density of 1.5 × 10^5^ cells/well and incubated in 2 ml of DMEM–low glucose supplemented with 1% BCS and antibiotics. Recombinant human LIF (10 ng/ml) or vehicle control (0.1% BSA in PBS) was added to the cells. Upon treatment for 48 h, BAE cells were examined for senescence‐associated β‐galactosidase activities using Senescence β‐Galactosidase Staining Kit (Cell Signaling, CAT# 9860) according to manufacturer’s instruction. Briefly, cells were fixed in 1 ml of fixative solution at room temperature for 10 min following removal of culture medium and wash with 2 ml of PBS. After two washes with PBS, cells were incubated in 1 ml of β‐galactosidase staining solution at 37°C overnight. Upon removal of staining solution, cells were immersed in 2 ml of 70% glycerol and stored at 4°C. Images from randomly chosen fields were obtained using Keyence Microscope BZ‐X710 (Keyence Corporation, Osaka, Japan). The experiment was carried out in duplicate/triplicate and repeated three times.

### RNA sequencing

5 × 10^5^ BCE and BAE cells were seeded in 6‐cm culture plates in 5 ml of maintenance medium overnight. Cells were then incubated in assay medium (for BCE, DMEM–low glucose supplemented with 10% BCS and antibiotics; for BAE, DMEM–low glucose supplemented with 1% BCS and antibiotics) for 3 h. Cells were subsequently incubated with LIF (10 ng/ml) or vehicle (0.1% BSA in PBS) for 6 h. Total RNAs were isolated using the RNeasy Plus Mini Kit (Qiagene, CAT# 74136) following manufacturer’s instructions and stored at −80°C. Library preparation for transcriptome sequencing, clustering/sequencing, and data analysis were performed by Novogene Inc. at Davis, CA. Briefly, a total amount of 3 μg RNA per sample was used as input material for the RNA sample preparations. Sequencing libraries were generated using NEBNext^®^ Ultra™ RNA Library Prep Kit for Illumina^®^ (NEB, USA) following manufacturer’s recommendations and index codes were added to attribute sequences to each sample. The clustering of the index‐coded samples was performed on a cBot Cluster GenerationSystem using HiSeq PE Cluster Kit cBot‐HS (Illumina) according to the manufacturer’s instructions. After cluster generation, the library preparations were sequenced on an Illumina Hiseq platform and 125/150 bp paired‐end reads were generated. The assembly of the Bos taurus genome Bos_taurus_UMD_3.1 (GenBank assembly accession: GCA_000003055.3) was used as the reference genome. Index of the reference genome was built using Bowtie v2.2.3 and paired‐end clean reads were aligned to the reference genome using TopHat v2.0.12. HTSeq v0.6.1 was used to count the reads numbers mapped to each gene. And then FPKM (expected number of fragments per kilobase of transcript sequence per millions base pairs sequenced) of each gene was calculated based on the length of the gene and reads count mapped to this gene. Differential expression analysis of two conditions/groups (two biological replicates per condition) was performed using the DESeq R package (1.18.0). The resulting *P*‐values were adjusted using the Benjamini and Hochberg’s approach for controlling the false discovery rate (Benjamini & Hochberg, 2000). Genes with an adjusted *P* < 0.05 found by DESeq were assigned as differentially expressed. FPKM data were then clustered using the log_10_(FPKM+1) values. Red denotes genes with high expression levels, and blue denotes genes with low expression levels. The color range from red to blue represents log_10_(FPKM+1) from large to small.

### STAT3 knockdown by siRNAs

BCE and BAE cells were plated onto six‐well culture plates at a density of 1.5 × 10^5^ cells/well and cultured overnight. Two milliliter of antibiotics‐free culture medium was used to replace the old medium. siRNAs, including siNegative (Ambion, CAT# AM4611), siSTAT3‐915 (Invitrogen, CAT# 361146C04), siSTAT3‐1492 (Invitrogen, CAT# 361146C05), and siSTAT3‐454 (Invitrogen, CAT# 384235A10), were mixed with Lipofectamine RNAiMAX reagent (ThermoFisher Scientific, CAT# 13778150) in Opti‐MEM™ I Reduced Serum Medium (Gibco, CAT# 31985062) according to manufacturer’s instructions. Briefly, a mix containing 25 pmol of siRNA, 7.5 μl of RNAiMAX reagent, and 125 μl of Opti‐MEM medium was used to transfect cells in each well, which makes a final siRNA concentration of 12.5 nM. A mix of RNAiMAX and Opti‐MEM was used as no siRNA control. Cells were incubated with siRNAs for 8 h and then cultured in fresh medium. Twenty‐four hours after transfection with siRNAs, cells were used for EC proliferation assays and RNA/protein extraction.

### Western Blotting

BCE and BAE cells were cultured in growth medium overnight. Medium was removed and cells were then washed twice with PBS. Recombinant human LIF was added to cells for 15 min, after a 3‐h incubation in the following media: DMEM–low glucose supplemented with 10% BCS, 2 mM glutamine and antibiotics for BCE cells, and DMEM–low glucose supplemented with 1% BCS and antibiotics for BAE cells. A higher serum concentration in the BCE incubation medium was employed given the lower viability of BCE compared to BAE cells. If applicable, small‐molecule inhibitors were added to the cells 1 h prior to LIF treatment. Cells were then lysed with RIPA lysis buffer (Life Technologies, CAT# 89901) plus protease and phosphatase inhibitor cocktail (ThermoFisher Scientific CAT# 78440). Equal amounts of protein were subjected to electrophoresis and then transferred onto PVDF membranes. Membranes were blocked with 5% non‐fat milk in TBST at room temperature for 1 h, incubated with primary antibodies indicated below in TBST containing 0.5% non‐fat milk at 4°C overnight then with secondary HRP‐conjugated antibodies (1:2,000, GE Healthcare) at room temperature for 1h. Signals were developed with SuperSignal™ West Chemiluminescent Substrate (ThermoFisher Scientific). Western blot was also confirmed with a digital blot scanning system (LI‐COR), which allowed us to image the phosphorylation bands and total bands at the same time without stripping. Primary antibodies used: anti‐phospho‐STAT3 (Cell Signaling, CAT# 9131, 1:3,000), anti‐STAT3 (Cell Signaling, CAT# 4904, 1:3,000), anti‐phospho‐ERK (Cell Signaling, CAT# 4376, 1:5,000), anti‐ERK (Cell Signaling, CAT# 4695, 1:5,000), anti‐phospho‐AKT Ser473 (Cell Signaling, CAT# 4060, 1:2,000), anti‐AKT (Cell Signaling, CAT# 4691, 1:2,000), and HRP‐conjugated anti‐beta‐actin (Sigma, CAT# AC‐15, 1;10,000). Two fluorescent antibodies were used for the co‐imaging of total and phosphorylated bands at the same time, IRDye 800CW goat anti‐mouse and IRDye 680RD goat anti‐rabbit secondary antibodies.

### RNA extraction and qRT‐PCR

BCE and BAE cells, after the indicated treatments, were lysed with Trizol reagent (Invitrogen, CAT# 15596026) and subjected to RNA extraction following manufacturer’s instructions. One microgram of total RNAs was reverse transcribed to cDNAs using the High‐Capacity cDNA Reverse Transcription Kit (Applied Biosystems, CAT# 4368814). Equal amounts (generally 10 ng/reaction) of cDNAs were subjected to qRT‐PCR analyses using the TaqMan Fast Advanced Master Mix (Applied Biosystems, CAT# 4444557) and the ViiA7 Real‐time PCR system. Relative mRNA levels of the examined genes were normalized to the internal control RPLP0 (Ribosomal Protein Lateral Stalk Subunit P0), determined by comparing with control sample group, and reported as fold changes. TaqMan gene expression assay probes were used: bovine RPLP0 (Bt03218086_m1), STAT3 (Bt03259865_m1), CTSL1 (Bt03257307_m1 and Bt03257309_m1), CTSB (Bt03259161_m1), MYC (Bt03260377_m1), JunB (Bt03246919_s1), CCNA2 (Bt03240503_g1), CCNB1 (Bt03237853_g1), and PIM1 (Bt03212957_m1). The experiment was carried out in triplicate and repeated three times.

### Annexin V staining for cell death

BAE cells were plated at a density of 2 × 10^4^ cells/well with 1 ml of culture medium in 12‐well plates and then incubated at 37°C overnight. After removal of culture medium, cells were incubated in 0.5 ml of DMEM–low glucose plus 1% BCS. LIF (10 ng/ml) and vehicle control (0.1% BSA in PBS) were added to the cells. Following LIF treatment for 24 h, cells were examined for cell death marker Annexin V using Annexin V‐Cy5 Apoptosis Staining Detection Kit (Abcam, CAT# ab14150) according to manufacturer’s instructions. Briefly, medium was removed, and 0.5 ml of Annexin V binding solution was laid over onto the cells. Cells were incubated at room temperature for 5 min following addition of 5 μl of Annexin V‐Cy5. Then, the staining solution was replaced with 0.5 ml of Annexin V binding solution. Imaging of Annexin V staining was performed using Keyence Microscope BZ‐X710 (Keyence Corporation, Osaka, Japan). Four random fields were selected and the percentages of Annexin V‐staining area in total cell‐covered area as indicatives for cell death were determined using ImageJ software. The experiment was carried out in triplicate and repeated three time.

### BrdU incorporation assay

BAE cells were plated at the density of 2 × 10^4^ cells/well with 1 ml of culture medium in a 12‐well plate with an 18‐mm poly‐D‐lysine‐treated coverslip in each well, incubating at 37°C overnight. After removal of culture medium, cells were incubated in 0.5 ml of DMEM–low glucose plus 1% BCS. LIF (10 ng/ml) and vehicle control (0.1% BSA in PBS) were added to the cells. Upon LIF treatment for 48 h, cells were subjected to incubation with 10 µM of BrdU for 4 h. Then, cells are subjected to BrdU immunofluorescence staining using a Fluor alexa‐488‐conjugated antibody against BrdU (Biolegend, CAT# 364106, 1:400). Briefly, cells were fixed with 3.7% formaldehyde in PBS at room temperature for 15 min. Cell DNAs were denatured with 1N HCl on ice for 10 min and 2N HCl at room temperature for 10 min following cell permeabilization with 0.1% Triton X‐100 in PBS (PBST). Cell coverslips were incubated with fluor alexa‐488 conjugated BrdU antibody in 5% goat serum‐PBST overnight at 4°C. Then, coverslips were mounted to glass slides with Fluoroshield Mounting Medium with DAPI (Abcam, CAT# ab104139). Imaging of BrdU staining was performed using Keyence Microscope BZ‐X710 (Keyence Corporation, Osaka, Japan). Four fields were randomly selected for each sample; the percentages of BrdU‐positive cells were determined by dividing the numbers of BrdU‐positive nuclei with the numbers of total nuclei (DAPI‐positive). The experiment was carried out in duplicate/triplicate and repeated three times.

### Purification of histidine‐tagged human LIF

For construction of human LIF expression plasmid, a nucleic acid fragment encoding the full‐length human LIF with a poly His‐tag at the C terminus was synthesized and inserted in pcDNA3.1(+) vector at NotI/XbaI site by GenScript USA, Inc. The Expi293 expression system (Life Technologies, A14524) was used to generate the conditioned media for purification. Pyrogen‐free reagents were employed. To inactivate endotoxin, columns and equipment (Akta Explorer System, GE Healthcare) were sanitized prior to use by exposure to 0.5 N NaOH for approximately 40 min, as described (Xin *et al*, 2021). LPS levels were measured by ToxinSensor™ Chromogenic LAL Endotoxin Assay Kit (GenScript, Cat. No. L00350).

Conditioned medium was adjusted to 20 mM sodium phosphate, pH 7.4, 0.5 M NaCl, 20 mM imidazole, and 0.01% Tween 20 before applying to HisTrap™ HP 1 ml column (GE Healthcare). The column was then washed with buffer containing 50 mM imidazole, and the elution of protein was achieved in 100 mM imidazole. Eluted protein was buffer exchanged into PBS containing 0.01% Tween 20. Recombinant protein was more than 95% pure, with LPS levels of approximately 0.02 EU/mg protein.

### Mouse macrophage isolation

Peritoneal resident macrophages were isolated from C57BL/6 mice as previous described (Zhang *et al*, 2008b). Mice were euthanized by rapid cervical dislocation. The abdomen of each mouse was dipped in 70% ethanol and the intact peritoneal wall was exposed manually. Ten‐ml PBS was injected into the peritoneal cavity and peritoneal fluid was then withdrawn. The peritoneal exudate cells (PEC) were transferred in a refrigerated centrifuge and spun for 10 min at 400 × *g*. Macrophages were resuspended and cultured with DMEM/F12 medium supplemented with 10% FBS. Three hours after isolation, fresh culturing media were changed and macrophages were incubated with LIF or vehicle control (PBS) for 16 h. Cells were harvested and RNA was prepared for qPCR analysis.

### Intravitreal injection of recombinant proteins

Six‐ to eight‐week male or P5 C57BL/6J mice were anesthetized with ketamine/xylazine cocktail. The indicated amounts of recombinant LIF (Sigma, CAT# SRP9001) in 1 μl of PBS and PBS vehicle control were injected intravitreally with a 33‐gauge Hamilton syringe. Seven (for adult mice) or three (for P5 mice) days after injection, animals were euthanized, eyes were enucleated, and fixed in 4% paraformaldehyde (PFA) for 15 min. Choroid–sclera complexes and retinas were separated and anti‐CD31 immunofluorescence (IF) or lectin labeling was performed to evidence the vasculature. For CD31 IF, rat anti‐mouse antibody (BD Biosciences, CAT# 550274) was diluted 1:100 and incubated overnight at 4°C. After 4‐h incubation with the Alexa Fluor‐488‐conjugated anti‐rat antibody (Life Technologies, CAT# A11006), whole mounts were imaged via the 488‐nm channel using Keyence Microscope BZ‐X710 (Keyence Corporation, Osaka, Japan) or A1R Confocal STORM super‐resolution system (Nikon). For lectin staining, Dylight‐488‐labeled lectin (Vector Laboratories, CAT# DL‐1174) was diluted at 1:200 and images were obtained using A1R Confocal STORM super‐resolution system (Nikon). Antibodies used for immunostaining: CD31 (BD, #550274) and NG2 (Invitrogen, #53‐6504‐82). Each experiment was repeated three times with similar results each time, and each treatment group consists of four or five individual samples. Before experiments, mice were allocated to different groups randomly and blindly. C57BL/6J mice were purchased from the Jackson Laboratory. All animal housing and experimental procedures were approved by the Institutional Animal Care and Use Committee (IACUC) of the University of California San Diego and conducted in accordance with the guidelines of the Animal Care Program (ACP).

### Measurement of retinal leakage

Adult mice were anesthetized as above described. rhLIF (100 ng/1 μl), rhVEGF_165_ (100 ng/1 μl), or PBS (1 μl) were intravitreally injected. The combination of LIF and VEGF at the same doses was also injected. After 7 days, the mixture of TRITC‐conjugated dextran (50 mg/ml, 0.1 ml, 70S; Sigma‐Aldrich) and FITC‐lectin (1 mg/ml, 0.1 ml; Vector laboratory, FL‐1171‐1) was injected into the tail vein, and animals were sacrificed by anesthetic overdose 15 min later. Eyes were removed and the retinas were dissected. Flat mounts were imaged by Keyence microscope. Quantification of leakage was defined as the ratio of TRITC–dextran area to FITC–lectin area.

### Immunofluorescent staining of retinal cryosections

LIF (50 ng) was intravitreally injected in C57bl/6 mice. Before experiments, mice were allocated to different groups randomly and blindly. After 2 h, mice were euthanized, and the eyes were enucleated and frozen in OCT compound (Sakura, 4583) and cooled by liquid nitrogen for cryosectioning. Frozen tissues were sliced into 10 μm sections. H&E and immunofluorescent staining was performed. Cryosections were dried thoroughly and fixed with 4% PFA for 10 min and then washed twice before blocking with 10% goat serum in PBS. Cryosections were incubated with anti‐mouse CD31 antibody (BD Biosciences, 550274) and rabbit anti‐phosphor‐STAT3 (Cell Signaling, # 9131) overnight. Fluorescent secondary anti‐rat and anti‐rabbit antibodies (Life Technologies A11006 and A11012) were used. All images were taken on Keyence microscope.

### Laser‐induced choroidal neovascularization (CNV)

C57BL/6J mice (6–8 weeks) in both genders were anesthetized with ketamine/Xylazine cocktail before laser treatment. Before experiments, mice were allocated to different groups randomly and blindly. CNV lesions were induced by laser photocoagulation using a diode laser (IRIDEX, Oculight GL) and a slit lamp (Zeiss) with a spot size of 50 µm, power of 180 mW, and exposure duration of 100 ms (Lambert *et al*, 2013; Silva *et al*, 2017). Four laser burns were typically induced at 3, 6, 9, and 12 o’clock position around the optic disc in each eye. IL family proteins and vehicle control were injected intravitreally, in a 1 μl volume. Ten days after laser induction, choroid–sclera complexes and retinas were separated and anti‐CD31 immunofluorescence (IF) was performed to evidence the vasculature by whole‐mount staining of both retina and choroidal tissues. For CD31 IF, rat anti‐mouse antibody BD 550274 was diluted 1:100 and incubated overnight at 4°C. After 4 h incubation with a secondary anti‐rat antibody (Life Technologies A11006), whole mounts were imaged at 488 nm. Quantification of neovascularization in lesion area and vascular density in retina was carried out by Image J. *P* values were assessed by Student’s *t*‐test (significant change, *P* < 0.05).

### Quantification of retina vasculature

Quantification of vasculature was carried out in software ImageJ. The threshold of vascular signal was adjusted, and vessel‐covered area was calculated by Analyze the Particle tools. Vascular areas were measured and normalized to the control group.

### Sodium Iodate model

Eight‐week‐old C57BL/6J male mice were anesthetized with ketamine/xylazine cocktail. Before experiments, mice were allocated to different groups randomly and blindly. Sterilized NaIO3 was administered as a single intravenous injection (20 mg/kg body weight) (Mizota & Adachi‐Usami, 1997; Hanus *et al*, 2016). Control mice were injected with PBS. PBS, LIF (50 ng), CT‐1 (different doses), or OSM (10 ng) was injected intravitreally in five mice groups. Five, 7, 9, and 12 days after injection, CC was monitored by OCT‐A system. Twelve days after injection, mice were sacrificed, and eyes were harvested for H&E and immunofluorescent staining. Avascular areas in the CC were analyzed using ImageJ.

### Optical coherence tomography angiography (OCT‐A) imaging

Optical coherence tomography angiography (OCT‐A) imaging of the retina in adult mice was performed 7 days after LIF injection, using a 1,300 nm optical coherence tomography angiography (OCT‐A) system developed by Dr. R.K. Wang’s group at University of Washington Seattle, in agreement with previously described methodology (Xu *et al*, 2019). Briefly, the swept laser operated in single‐longitude mode with a 90 nm bandwidth centered at 1,300 nm and 200 kHz A‐line rate was used to scan mouse retina and to generate images of vasculature in a field of view of 1.5 × 1.5 mm^2^. 2,500 B‐frames were captured at 500 cross‐sections with five repeated B‐frames at each cross‐section. The B‐frames were segmented and choriocapillaris layer was extracted as 3–5 pixels below the RPE layer. To quantify the retinal vascular density, retinal and choroidal layers in 3D structure OCT scans were separated by the hyper‐reflecting retinal pigment epithelium (RPE). The maximum intensity projection was generated. The vessel density was then determined by calculating the percentages of vessel‐covered area in total area of view using the ImageJ software.

### Mouse choroid explants

Three‐week‐old C57BL/6J male mice were anesthetized with ketamin/xylazine and euthanized by cervical dislocation. Eyes were enucleated and transferred in medium (EGM‐2 MV, Lonza, cat. CC‐3202). After removing cornea, lens, and retina, the eyecup that contains choroid and sclera was cut into approximately 1 mm × 1 mm squares. The squares were placed into 30 μl Matrigel (Corning, cat. 354234) in 24‐well plates. The explants were incubated in a cell culture incubator at 37°C with 5% CO_2_ for 10 min for the Matrigel to solidify, subsequently 0.5 ml medium was added and explants were allowed to grow for 6 days. The explants were then harvested and incubated with 2 µg/ml Dispase/Collagenase (Sigma, cat.10269638001) for 1 h to dissolve Matrigel and to disperse cells. The cell solutions were filtered through a 70 µm cell strainer (Sigma, CLS431751) and washed with PBS containing 0.1% BSA.

### FACS

FACS was used to detect cell subpopulations from isolated mouse choroidal explants. Cells were stained with FITC CD13 (BD Pharmingen, cat. 558744), APC CD45 (Biolegend, cat. 103111), PE CD31 (Biolgend 102507), or PE PDGFR (Biolegend, cat 136005) for 1 h in PBS containing 0.1% BSA. Then the stained cells were washed and stained with Propidium Iodide (Invitrogen, P3566) to detect dead cells. The cells were analyzed and sorted with a FACSariaII cell sorter. UltraComp eBeads (Invitrogen, cat. 01‐2222‐45) were used for compensation, FMO controls were used for gating. FACS results were analyzed using FlowJo 7.6.2 software (FlowJo, LLC., Ashland, Oregon).

### Statistical analysis

Experiments were repeated at least three times with similar results except for the mass spectrometry analyses. Bar charts represent mean ± standard deviation (SD). For comparison between the only two groups in a study, two‐tailed Student’s *t*‐test was performed. For comparisons among groups in a study with more than two groups of data, one‐way ANOVA with multiple comparisons was performed. For comparisons among groups in a study with two or more variables, two‐way ANOVA with multiple comparisons was performed. *P* < 0.05 was deemed as statistically significant. All statistical analyses were performed using Graphpad Prism software package.

### Datasets for single‐cell analysis

Log‐scale normalized choroidal single‐cell gene expression data from Voigt *et al* (Voigt *et al*, 2019) were downloaded from GSE135922. Data from donors 4–7 (CD31^+^ enriched) were clustered using Seurat (Stuart *et al*, 2019). Cluster cell types were determined using known markers (Newman *et al*, 2019; Voigt *et al*, 2019). Seurat was used to create violin plots of the expression of genes of interest in each cell type.

These choroidal single‐cell data were used to select receptors and transcription factors whose neighbors within the Parsimonious Composite Network (PCN) (Huang *et al*, 2018) were enriched for differentially expressed genes between endothelial and other cell types. The correlation matrix including the enriched receptors and transcription factors was calculated. Agglomerative clustering was then performed on this correlation matrix, splitting the genes into six clusters. The cluster containing KDR and FLT1, the primary receptors responsible for the VEGF pathway, included 18 receptors, and the statistical significance for enrichment for angiogenesis genes (GO:0001525) was calculated using the hypergeometric test.

## Author contributions

QL, PL, and NF designed research; QL, PL, LPG, NB, LL, SD, GP, CP, HX, TD, and NF performed research; AH, GP, CP, RKW, and QL analyzed data; QL, PL, and NF wrote the manuscript.

## Conflict of interest

NF, PL, and QL are inventors in a patent application describing this technology. The technology has been licensed to a start‐up company. GP and CP own equity in Salgomed, Inc.

## Supporting information



Appendix

Table EV1

Table EV2

Dataset EV1

Dataset EV2

Dataset EV3

Source Data for Figure 1

Source Data for Figure 2

Source Data for Figure 3

Source Data for Figure 4

Source Data for Figure 5

## Data Availability

RNA sequencing datasets generated in this study are available on NCBI Gene Expression Omnibus with the accession number GSE186481 (https://www.ncbi.nlm.nih.gov/geo/query/acc.cgi?acc=GSE186481).
