# Supplementary material for: LIF, a mitogen for choroidal endothelial cells, protects the choriocapillaris: implications for prevention of geographic atrophy
Source: EMBO Mol Med. 2021 Nov 15;14(1):e14511. doi: 10.15252/emmm.202114511 (PMC8749470; doi:10.15252/emmm.202114511)
Supplement: Supplementary file 1 — Appendix [file EMMM-14-e14511-s009.pdf]

Table of content

Appendix Figure S1-17
Appendix Table S1

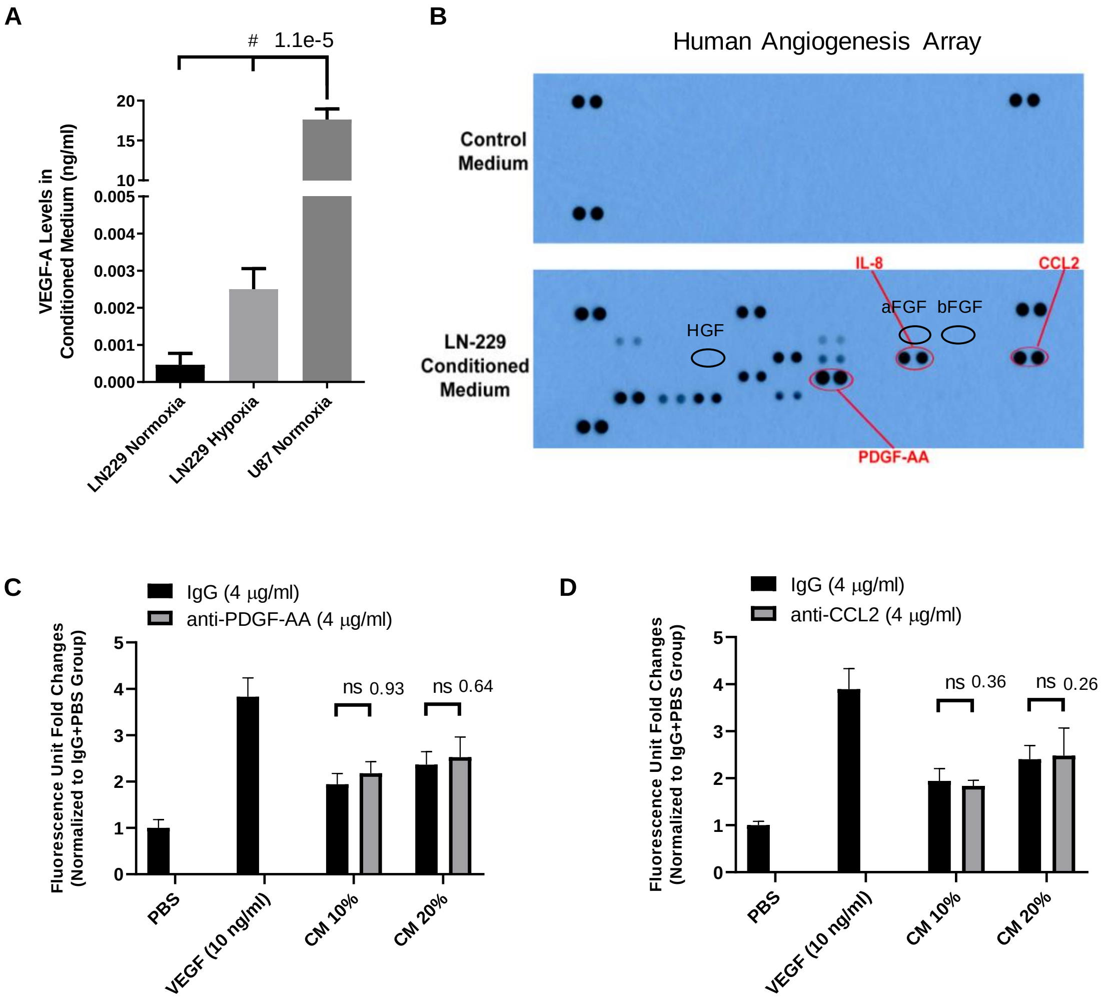

Appendix Figure S1. **Release of angiogenic factors by LN-229 cells.**

A) LN-229 cells secreted little VEGF compared to U-87MG cells. LN-229 and U-87MG cells were incubated in normoxic or hypoxic conditions for 72h. VEGF concentrations in the conditioned media were determined by ELISA Three independent experiments were carried out with three technical replicates in each experiment.

B) PDGF-AA, CCL2 and IL-8 are the most abundant angiogenic factors in LN-229 CM. The control medium and LN-229 CM were concentrated five times using a filter with a pore size of 10 kDa. 0.5 ml of concentrated LN-229 CM and concentrated control medium were applied to human angiogenesis factor antibody array (R&D Systems, CAT# ARY007) following

manufacturer's instructions. The layout scheme of the human angiogenesis factor antibody array and the chemiluminescence blot of LN-229 CM angiogenesis factor antibody array are shown.
C) and **D**) Antibodies neutralizing PDGF-AA and CCL2 could not block LN-229 CM mitogenic activity in BCE cells. BCE cells pre-incubated with anti-PDGF-AA (μg/ml) (**C**) or anti-CCL2 (μg/ml) (**D**) and isotype goat IgG (4 μg/ml) for 1 hours were treated with indicated concentrations of LN-229 CM, PBS, or 10ng/ml VEGF. Three independent experiments were carried out with three technical replicates in each.
Data information: Bars and error bars represent mean ± SD. #, $p \leq 0.0001$; ns, not statistically significant. Two-way ANOVA test was used as statistical test.

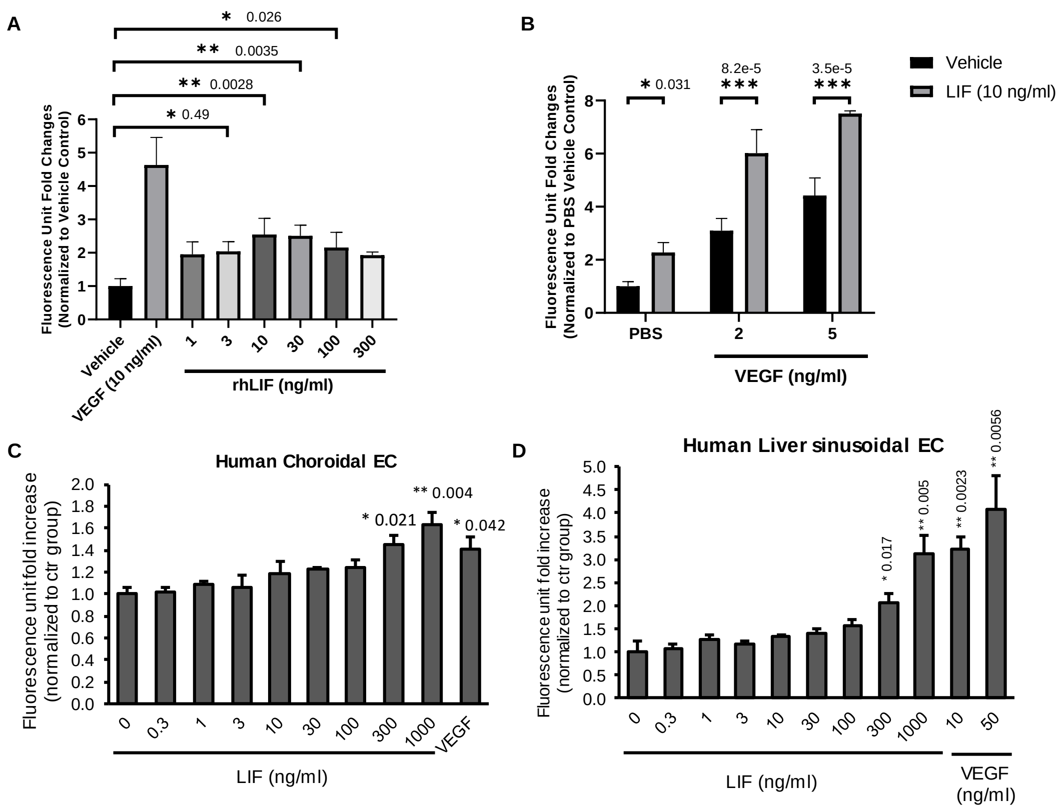

Appendix Figure S2. Recombinant LIF stimulates BRE and human EC cell growth.

A) BRE cells were treated with the indicated concentrations of recombinant human LIF, vehicle control, or 10ng/ml VEGF. Cell growth was measured at day 6. Cell growth in each treatment group was normalized to the vehicle control group. Three independent experiments were carried out with three technical replicates in each experiment.

B) Synergistic effects of LIF and VEGF on BRE cell growth. BRE cells were treated with the indicated concentrations of recombinant human VEGF in the presence of 10 ng/ml LIF or vehicle control. Cell growth was determined at day 6. Cell growth in each treatment group was normalized to the vehicle plus PBS control group. Three independent experiments were carried out with three technical replicates in each experiment.

C) Recombinant human LIF stimulated growth of HCECs in a dose-dependent manner. HCECs were cultured in the presence of vehicle, VEGF (10 ng/ml) and the indicated concentrations of

recombinant human LIF (rhLIF). Three independent experiments were carried out with three technical replicates in each experiment.

D) LIF induced human liver sinusoidal EC growth in a concentration-dependent manner. Concentrations are indicated in graph.

Data information: Bars and error bars represent mean ± SD. *, $p < 0.05$; **, $p < 0.01$; ***, $p < 0.001$. Two-way ANOVA test was used as statistical test. A representative experiment is shown from three independent studies.

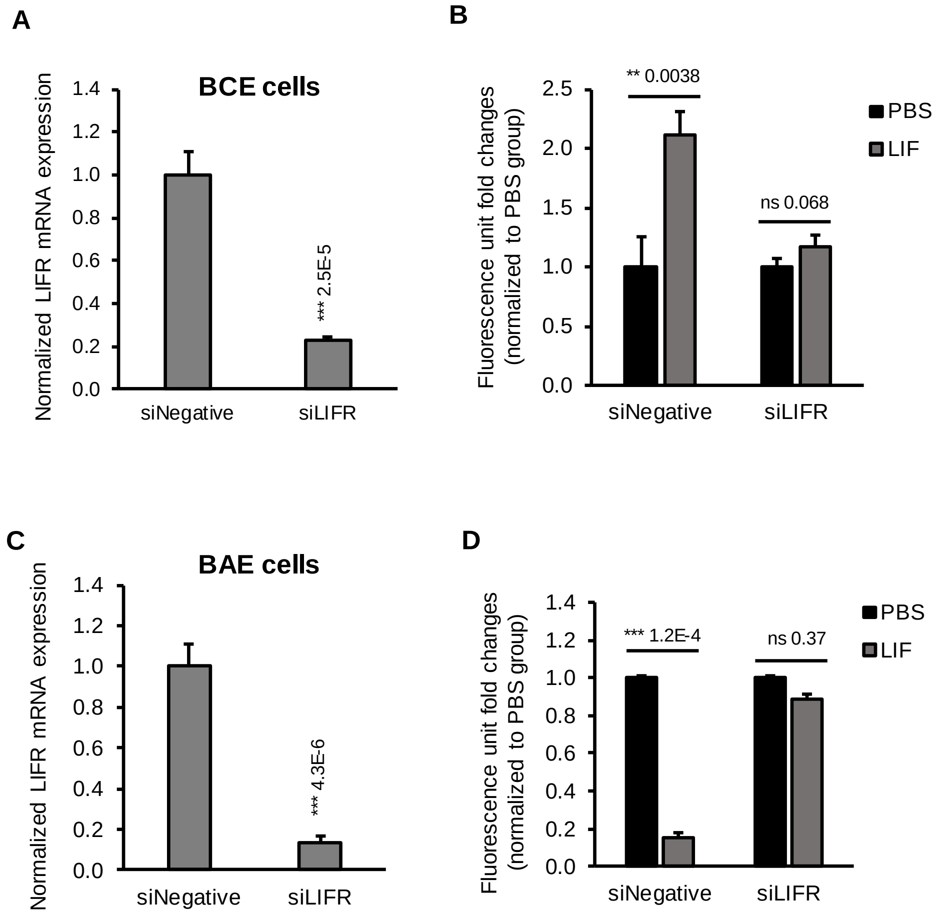

Appendix Figure S3. LIF effects on BCE or BAE cells are blocked by LIFR knockdown.

A) LIFR was knocked-down by siRNA in BCE cells. A control siRNA was used as negative control (siNegative). Three independent experiments were carried out with three technical replicates in each experiment.

B) LIF-induced BCE proliferation was nearly abolished by LIFR siRNA. Three independent experiments were carried out with three technical replicates in each experiment.

C) LIFR knock-down in BAE by siRNA. A control siRNA was used as negative control (siNegative). Three independent experiments were carried out with three technical replicates in each experiment.

D) LIF-induced proliferation in BAE cells was largely abolished by LIFR siRNA. n = 3.

Data information: Bars and error bars represent mean ± SD. A representative experiment is shown from three independent studies. **, $p < 0.01$; ***, $p < 0.001$. Three independent

experiments were carried out with three technical replicates in each experiment. Two-way ANOVA test was used as statistical test.

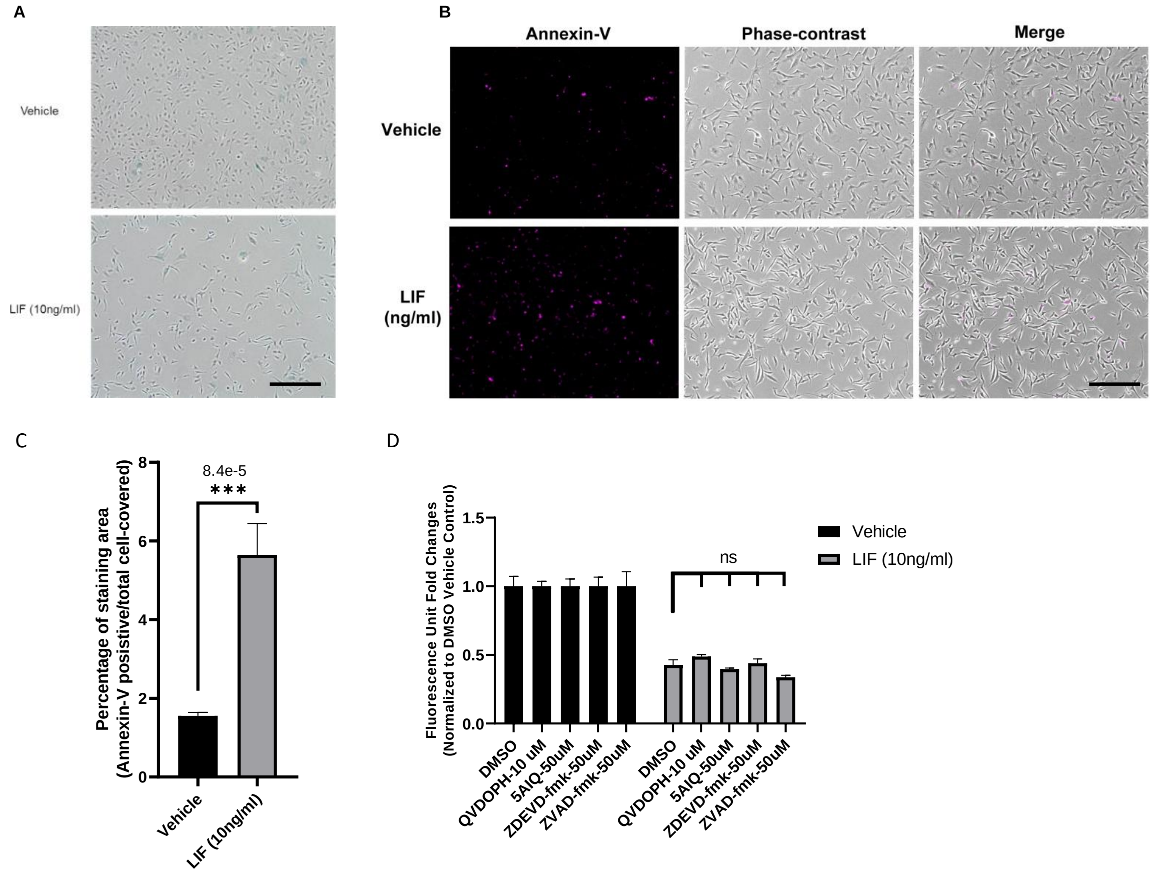

Appendix Figure S4. LIF does not induce senescence or caspase-dependent apoptosis in BAE cells.

A) After treatment with LIF for 48 hours, BAE cells were examined for senescence associated β-galactosidase activities. Senescence-associated-β-galactosidase activity was indicated with blue-colored staining in the representative images. Scale bar = 200 µm.

B) and **C**). LIF treatment induced cell death in BAE cells. Upon treatment with LIF (10 ng/ml) or vehicle for 24 hours, BAE cells were stained with Annexin V-Cy5. Representative images are shown in B. Scale bar = 200 µm. Percentages of Annexin V-positive area versus total cell-covered area were calculated and presented in C. Bars and error bars represent mean±SD. Three independent experiments were carried out with three technical replicates in each experiment.

D) Inhibitors of caspases or PARP did not rescue LIF-induced BAE cell death. BAE cells pre-incubated with indicated concentrations of inhibitors (in DMSO) and DMSO control for 1 hour were treated with LIF (10 ng/ml) or vehicle control. Cell growth was determined at day 6. Cell

growth for each LIF-treated group was normalized to the vehicle control group with the same inhibitor treatment. Three independent experiments were carried out with three technical replicates in each experiment. ns, $p > 0.05$, not statistically significant. Two-way ANOVA test was used as statistical test.

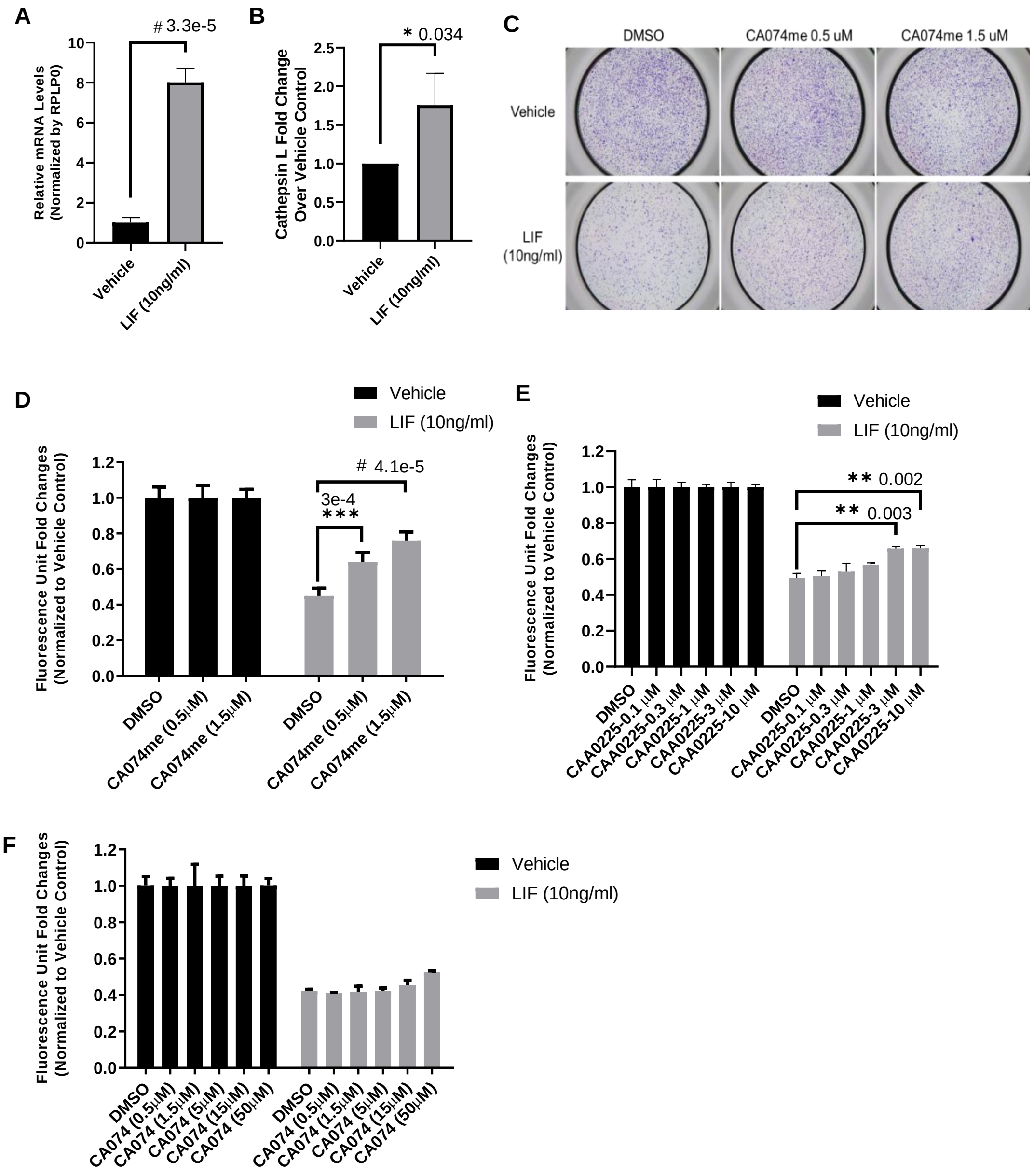

Appendix Figure S5. LIF inhibits BAE cell growth *via* regulating cell death.

A) LIF induces cathepsin L expression in BAE cells. Following treatment with LIF (10 ng/ml) or vehicle for 24 hours, qRT-PCR was performed to examine cathepsin L (CTSL) mRNA levels in BAE cells. The CTSL level in vehicle group was set as 1. CTSL mRNA levels in each sample were compared to the vehicle group and are presented as fold changes. Three independent experiments were carried out with three technical replicates in each experiment.

B) Total proteins from LIF treated BAE cells were used for bovine cathepsin L ELISA. The cathepsin L protein levels in the vehicle-treated group were set as 1. Induction fold changes for cathepsin L protein (LIF-treated samples versus vehicle group) were calculated and fold changes were shown. Three independent experiments were carried out with three technical replicates in each experiment.

C) The inhibitor of cathepsin B/L CA074me alleviated LIF-induced cell death in BAE cells. BAE cells pre-incubated with indicated concentrations of CA074me (in DMSO) and DMSO control for 1 hour were treated with LIF (10 ng/ml) and vehicle control. After treatment for 6 days, cells were fixed and stained with 1% crystal violet for 5 minutes. Representative images are shown here.

D) and **E**) Cathepsin L inhibitors CA074me and CAA0225 alleviated LIF-induced BAE cell growth inhibition. BAE cells pre-incubated with indicated concentrations of CA074me and CAA0225 for 1 hour were treated with vehicle, LIF (10 ng/ml) and VEGF (10 ng/ml). Cell growth was analyzed after 6 days. Three independent experiments were carried out with three technical replicates in each experiment.

F) Cathepsin B-selective inhibitor CA074 did not rescue LIF-induced cell death in BAE cells. BAE cells pre-incubated with the indicated concentrations of CA074 (in DMSO) and DMSO control for 1 hour were treated with LIF (10 ng/ml) and vehicle control. Cell growth was determined at day 6. Cell growth for each LIF-treated group was normalized to the corresponding vehicle control group Three independent experiments were carried out with three technical replicates in each experiment.

Data information: Bars and error bars represent mean ± SD. *, $p < 0.05$; **, $p < 0.01$; *** $p < 0.001$; #, $p \leq 0.0001$; ns, not statistically significant. Two-way ANOVA test was used as statistical test.

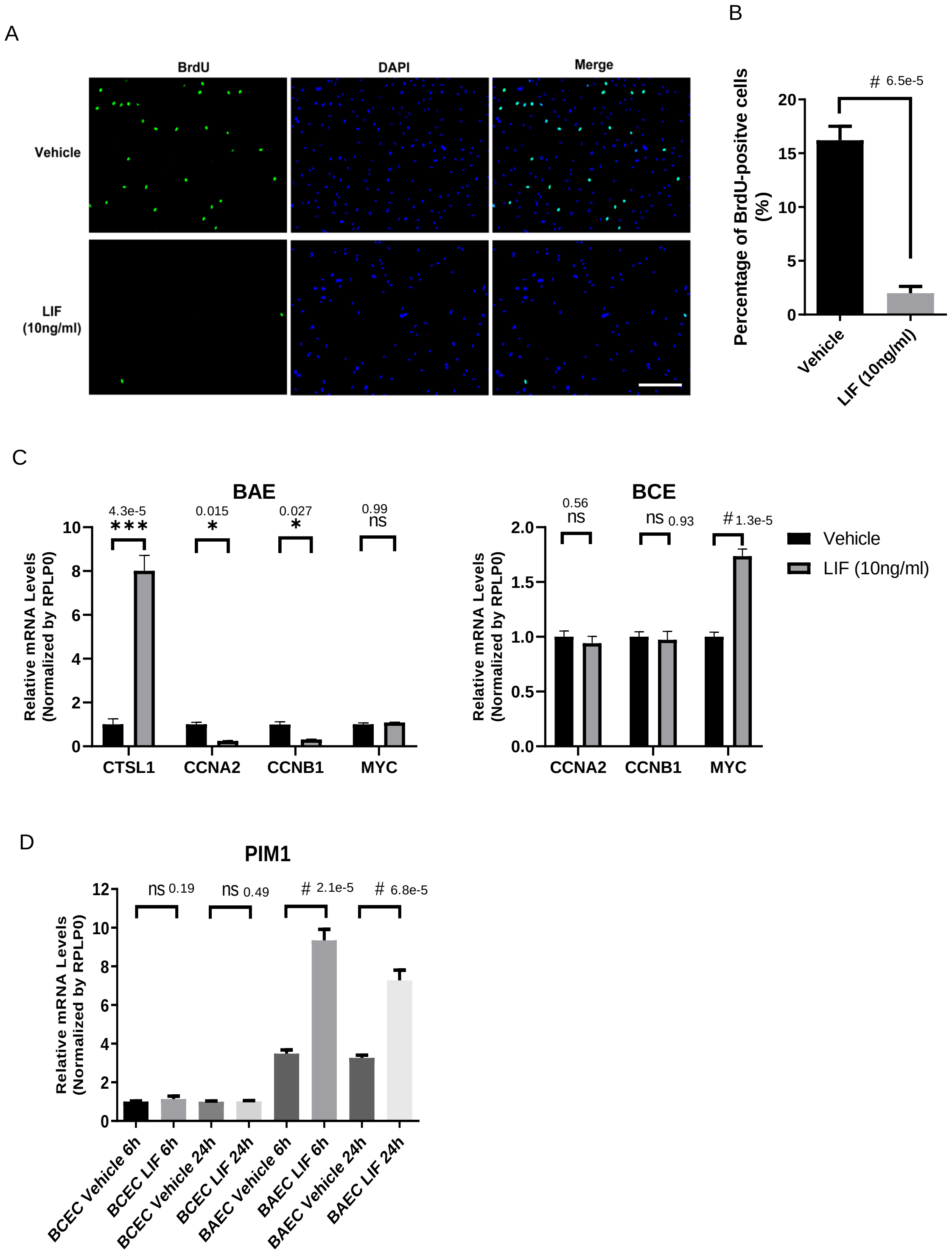

Appendix Figure S6. LIF induces cell cycle arrest in BAE cells.

A) and **B**) LIF treatment reduced BrdU incorporation in BAE cells. Upon treatment with LIF (10 ng/ml) and vehicle for 48 hours, BAE cells were incubated with 10 μM of BrdU for 4 hours.

Representative images of BrdU incorporation detected with an Alexa Fluor-488 conjugated BrdU antibody are shown in A. Scale bar = 200 µm. Percentages of BrdU positive nuclei versus DAPI-stained total nuclei were calculated and shown in B Three independent experiments were carried out with three technical replicates in each experiment.

C) Repression of cyclin A and B expression by LIF in BAE. BAE and BCE cells were treated with LIF (10 ng/ml) and vehicle for 24 hours. qRT-PCR was performed to examine CTSL1, CCNA2, CCNB1 and MYC mRNA levels. For each gene probe, the vehicle-treated group levels were set as 1. mRNA levels in LIF-treated samples were normalized to the vehicle group Three independent experiments were carried out with three technical replicates in each experiment.

D) LIF-treatment induced PIM1 gene expression in BAE. BCE and BAE were treated with LIF (10 ng/ml) or vehicle control for 6 hours and 24 hours. qRT-PCR was performed as described above. PIM1 expression level for each group was normalized to that of BCEC with vehicle treatment for 6 hours Three independent experiments were carried out with three technical replicates in each experiment.

Data information: Bars and error bars represent mean ± SD. *, $p < 0.05$; *** $p < 0.001$; #, $p \leq 0.0001$; ns, not statistically significant.   Two-way ANOVA test was used as statistical test.

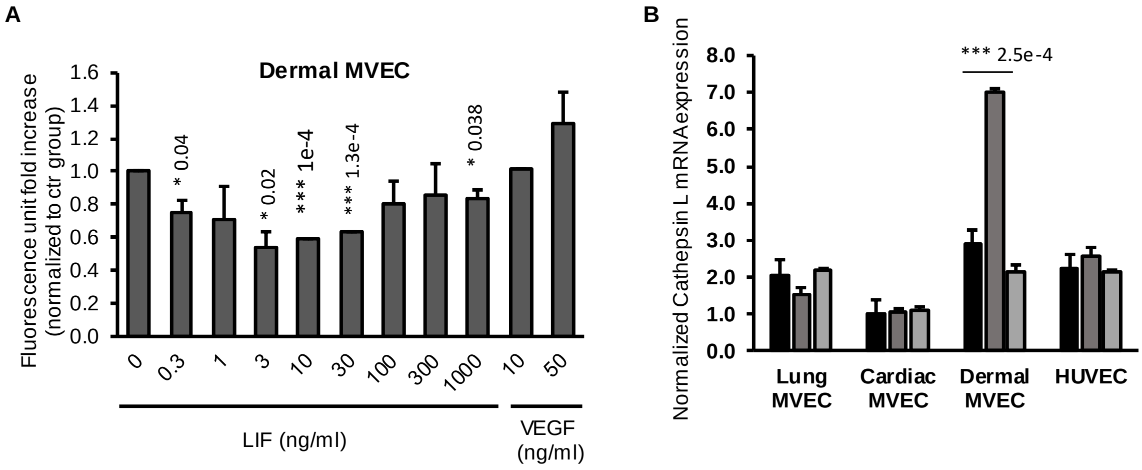

Appendix Figure S7. LIF-induced inhibition of human dermal EC growth is associated with upregulation of cathepsin L.

A) LIF inhibited human dermal EC growth in a concentration-dependent manner. VEGF was used as a positive control. Three independent experiments were carried out with three technical replicates in each experiment.

B) Cathepsin L was upregulated in dermal microvascular ECs (Dermal MVEC). RNA samples from multiple human ECs were used for TaqMan qPCR assay. LIF 50 ng/ml and 500 ng/ml groups were analyzed for Cathepsin L expression. PBS was used as vehicle control. Three independent experiments were carried out with three technical replicates in each experiment. Data information: Bars and error bars represent mean ± SD. A representative experiment is shown from three independent studies. *, $p < 0.05$; *** $p < 0.001$. Two-way ANOVA test was used as statistical test.

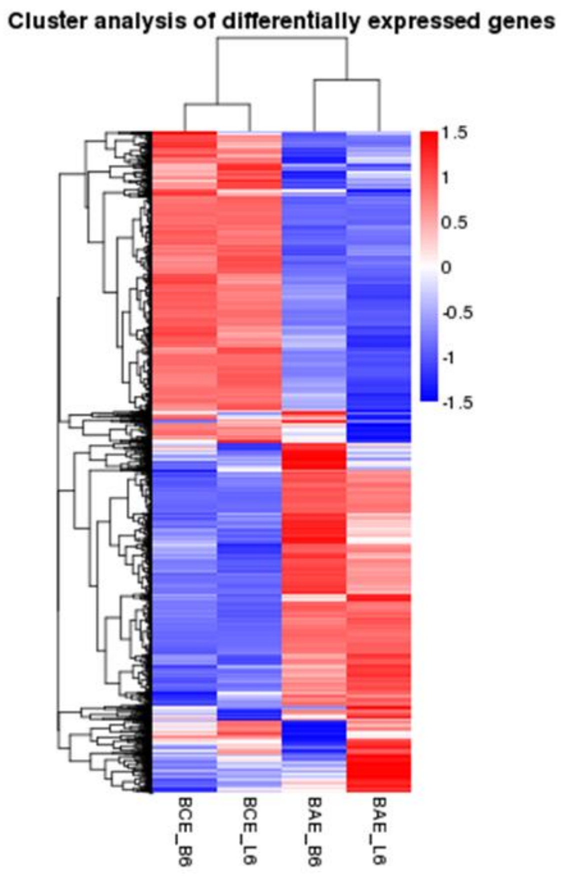

Appendix Figure S8. BCE and BAE cells show different gene expression patterns in response to LIF treatment.

The FPKM cluster analysis of RNA-seq data revealed distinct gene expression patterns in BAE and BCE cells treated with LIF for 6 hours (see supplementary files 1-4). BCE_B6, BCE cells treated with vehicle for 6 hours; BCE_L6, BCE cells treated with 10 ng/ml LIF for 6 hours; BAE_B6, BAE cells treated with vehicle for 6 hours; BAE_L6, BAE cells treated with 10 ng/ml LIF for 6 hours. Two individual samples were used for each treatment group.

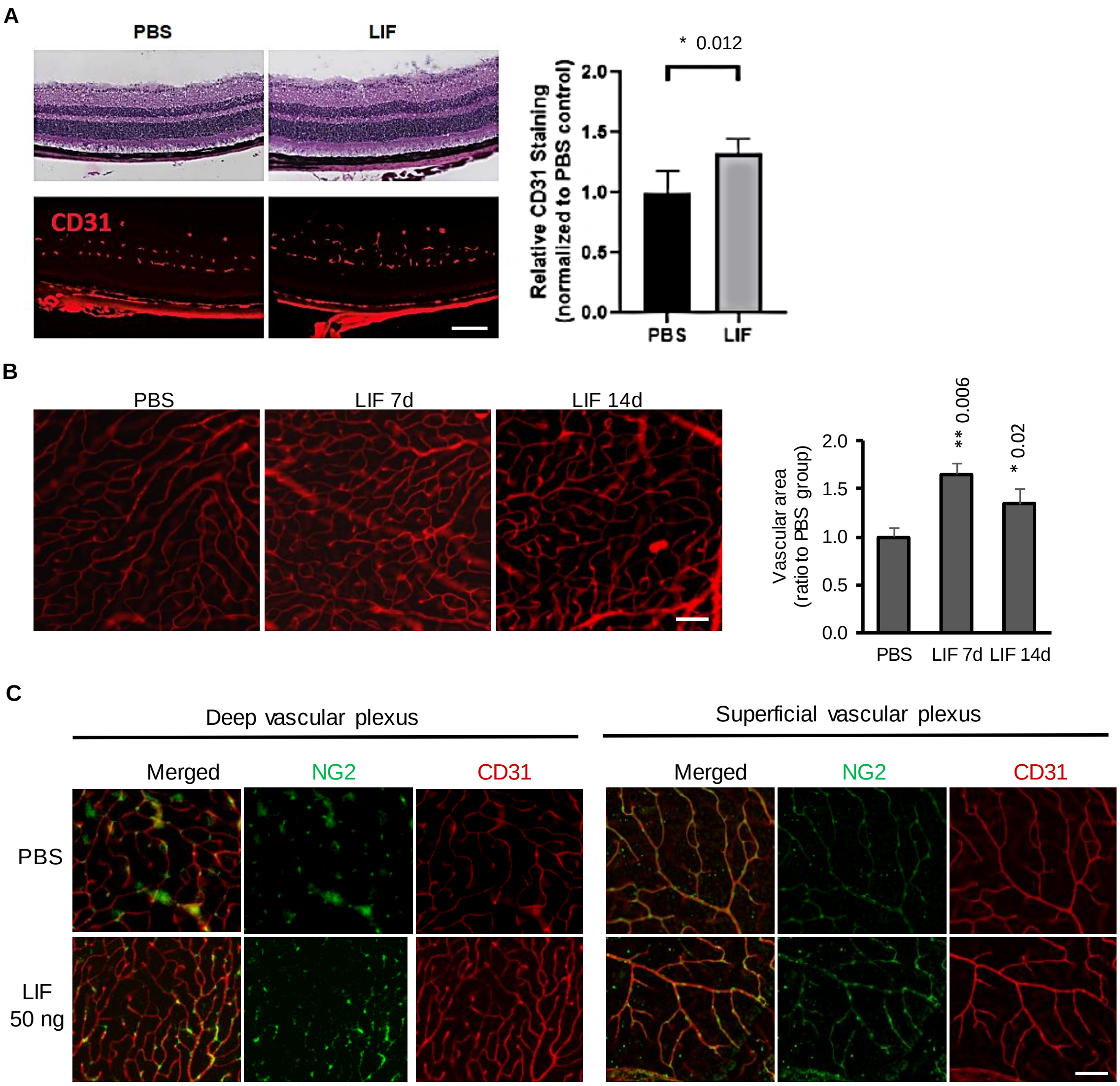

Appendix Figure S9. LIF-induced retinal blood vessels are covered by pericytes

A) LIF treatment increases vascular density in the mouse retina. Adult mice were intravitreally injected with LIF (50 ng) or vehicle. Seven days after injection, frozen sections of mouse eyes were subjected to H&E staining and CD31 IF staining. Scale bar = 100 µm. Representative images and quantification of CD31-positive using ImageJ software were shown in the right panel. Four independent experiments were carried out with five technical replicates in each experiment.

B) LIF induced retinal angiogenesis at both 7 days and 14 days. 50 ng of in-house LIF with low-endotoxin levels was injected in mouse vitreous. For the 14 days timepoint, LIF was injected a

second time at day 7. Retinal vasculature was investigated by whole mount staining of CD31. Scale bar = 100 µm. Quantification of vascular density is shown. Three independent experiments were carried out with five technical replicates in each experiment.

C) LIF-induced retinal vessels are covered by pericytes. 50 ng of our in-house LIF with low-endotoxin levels was injected and retinas were harvested 7 days after injection. Pericyte marker, NG2, and CD31 were co-stained on retina flat mount. Scale bar = 100 µm.

Data information: Bars and error bars represent mean ± SEM. A representative experiment is shown from three independent studies. *, $p < 0.05$; **, $p < 0.01$. Two-way ANOVA test was used as statistical test.

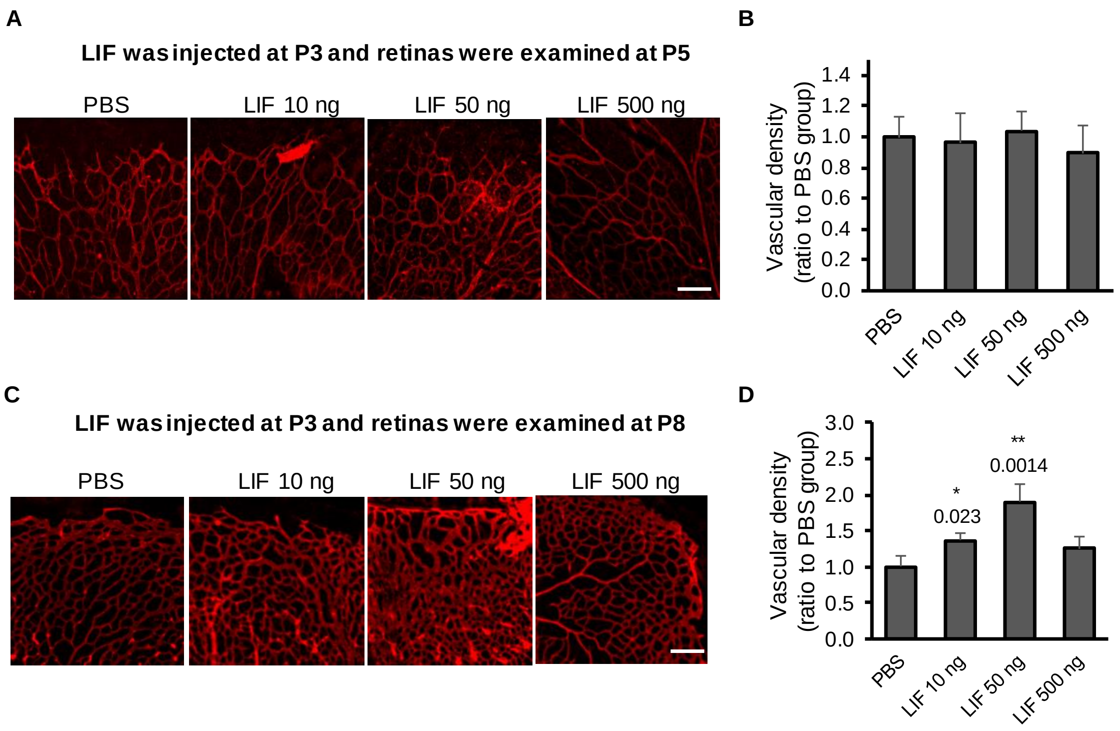

Appendix Figure S10. Effects of LIF on retinal angiogenesis in P3 neonatal mice.

A, B) Our in-house LIF with low-endotoxin levels was injected at different doses in neonatal mice at P3. Retinas were harvested and stained with CD31 antibody at P5. Scale bar = 100 µm. There was no significant differences after LIF injection. Vascular density was quantified by ImageJ and bar graph is shown in **B**) Three independent experiments were carried out with five technical replicates in each experiment

C, D) Different doses of recombinant LIF with low-endotoxin levels (see Materials and Methods) were intravitreally injected in neonatal mice at P3. Retinas were harvested and stained with anti-CD31 antibody at P8. Scale bar = 100 µm. There was a significant induction of angiogenesis at P8 after LIF injection. Vascular density was quantified by ImageJ and bar graph is shown in **D**), n = 5. Three independent experiments were carried out with five technical replicates in each experiment *, $p < 0.05$; **, $p < 0.01$. Two-way ANOVA test was used as statistical test.

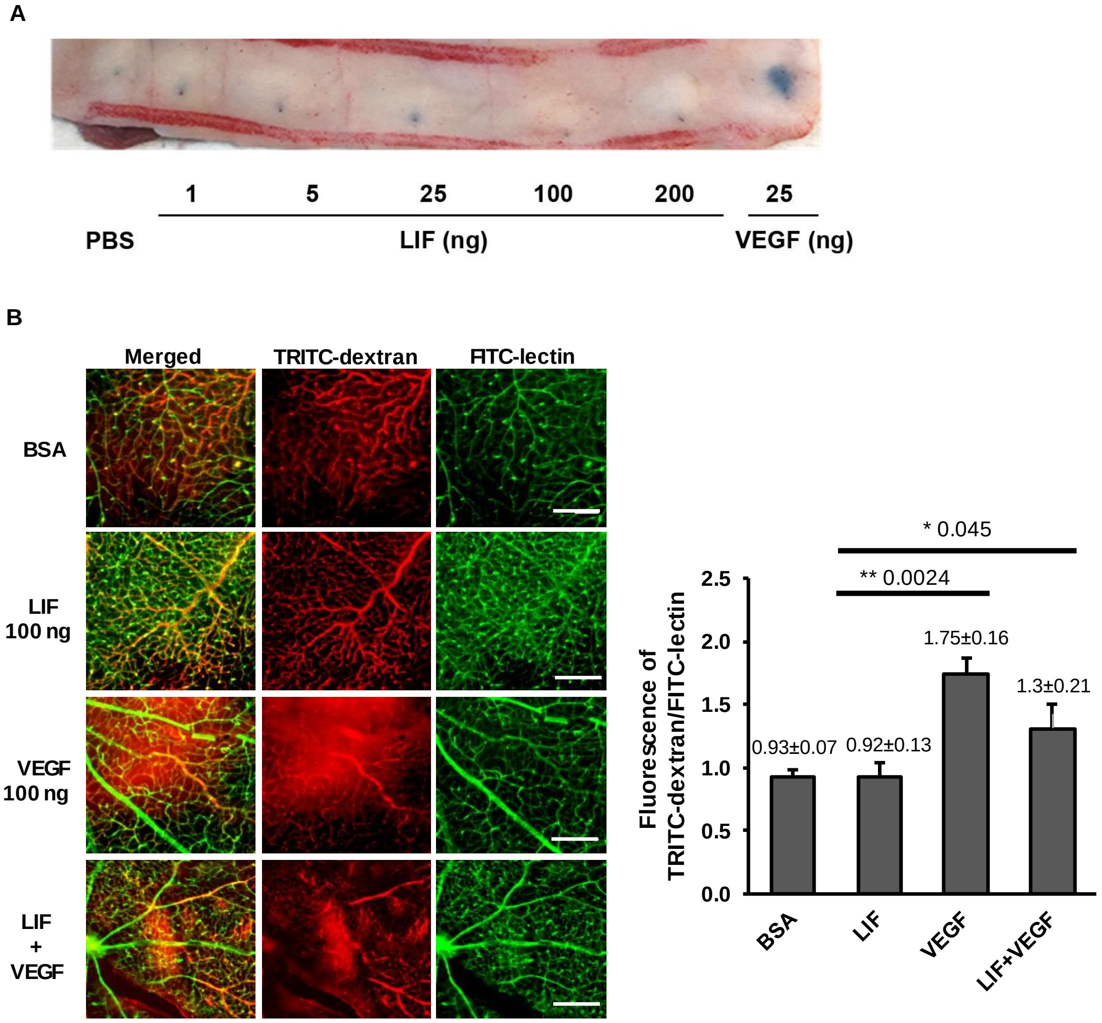

Appendix Figure S11. **LIF does not induce vascular permeability in the guinea pig skin or in the mouse retina**.

A) Hairless male guinea pigs (Crl: HA-Hrhr/IAF, 450–500 g, Charles River Laboratories) were anesthetized by intraperitoneal (i.p.) administration of xylazine (5 mg/kg) and ketamine (75 mg/kg). The animals then received an intravenous injection (penile vein) of 1 ml of 1% Evans blue dye. After 15 min, intradermal injections (0.05 ml/per site) of different doses (1, 5, 25, 100, 200 ng per injection site) of rhLIF in PBS were administrated into the area of trunk posterior to the shoulder. 0.05 ml of PBS and 25 ng of VEGF in 0.05 ml of PBS were injected as negative and positive controls. 30 min after the intradermal injections, animals were euthanized by i.p.

injection of pentobarbital (200 mg/kg). Skin tissues were dissected from the connective tissues and photographed, n=2.

B) Vascular leakage in the mouse retina. LIF (100 ng), VEGF (100 ng) or the combination of LIF and VEGF was injected intravitreally (0.1% BSA/PBS as control). 7 days later, TRITC-dextran was intravenously injected (see Methods) to evidence vascular leakage. Retinal vasculature was labeled by FITC-lectin, n=5. Three independent experiments were carried out with five technical replicates in each experiment. Scale bar = 100 µm. Bars and error bars represent mean ± SEM. A representative experiment is shown from three independent studies. *, $p < 0.05$; ***, $p < 0.001$. Two-way ANOVA test was used as statistical test.

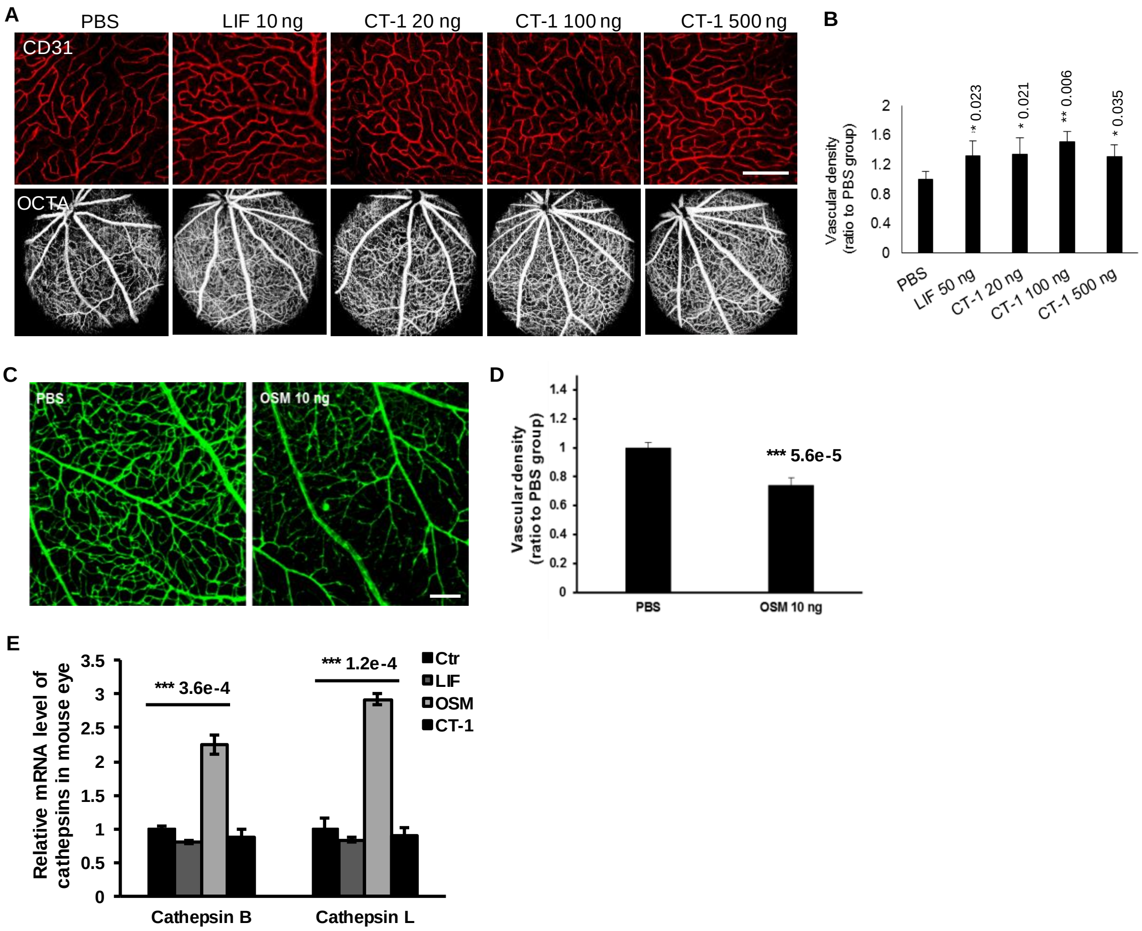

Appendix Figure S12. **Effects of LIF, CT-1 and OSM on retinal angiogenesis in adult mice.**

A) Recombinant LIF (50 ng) and different doses of CT-1 in 1µl and PBS were injected intravitreally. Retinal vasculature was evidenced by OCT-A imaging and CD31 immunofluorescent staining, n=5. Scale bar = 100 µm.

B) Retinal flat mount stainings were imaged using confocal microscope. Quantification of vessels was performed using Image J. Three independent experiments were carried out with five technical replicates in each experiment.

C) and **D**) Recombinant OSM (10 ng) in 1µl and PBS vehicle control were injected intravitreally. Retinal vasculature was visualized and quantified with CD31 immunofluorescent staining, n=5. Scale bar = 100 µm. Three independent experiments were carried out with five technical replicates in each experiment.

E) OSM-treatment induced cathepsin B and L gene expression in mouse eyes. LIF, OSM, CT-1 or vehicle control was intravitreally injected in mouse eyes. After 24 hours, qRT-PCR was performed as described above. Cathepsin B and L expression levels for each group were normalized to that of vehicle treatment, n=3. Three independent experiments were carried out with three technical replicates in each experiment.

Data information: Bars and error bars represent mean ± SEM. A representative experiment is shown from three independent studies. *, $p < 0.05$, **, $p < 0.01$; *** $p < 0.001$. Two-way ANOVA test was used as statistical test.

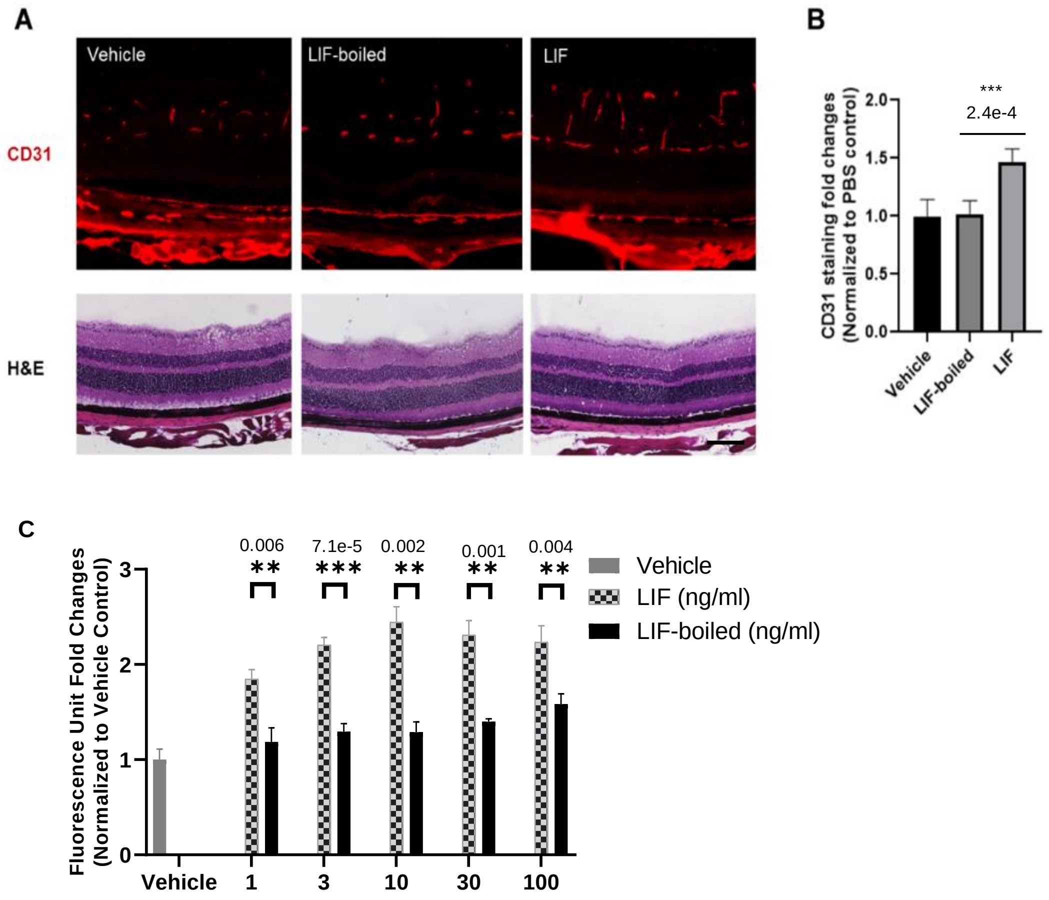

Appendix Figure S13. Heat-inactivation abolished LIF effects *in vivo* and *in vitro*.

A, B) LIF (Sigma) was heat-inactivated by boiling at 95℃ for 2 hours. Adult mice were intravitreally injected with 50 ng of untreated or boiled LIF. After 7 days, frozen sections of mouse eyes were subjected to H&E and CD31 IF staining. Representative images are shown in **A**). Scale bar = 100 µm. Quantification of CD31-positive area was performed using ImageJ software. Untreated and boiled LIF groups were compared to vehicle in **B**, n=4-5. Three independent experiments were carried out with four to five technical replicates in each experiment.

C) BCE cells were cultured in the presence of vehicle, the indicated concentrations of LIF or boiled LIF, for 6 days. Cell growth in each treatment group was normalized to that of the vehicle group, n=3. Three independent experiments were carried out with three technical replicates in each experiment.

Data information: Bars and error bars represent mean ± SEM. A representative experiment is shown from three independent studies. ***, $p < 0.001$; **, $p < 0.01$.

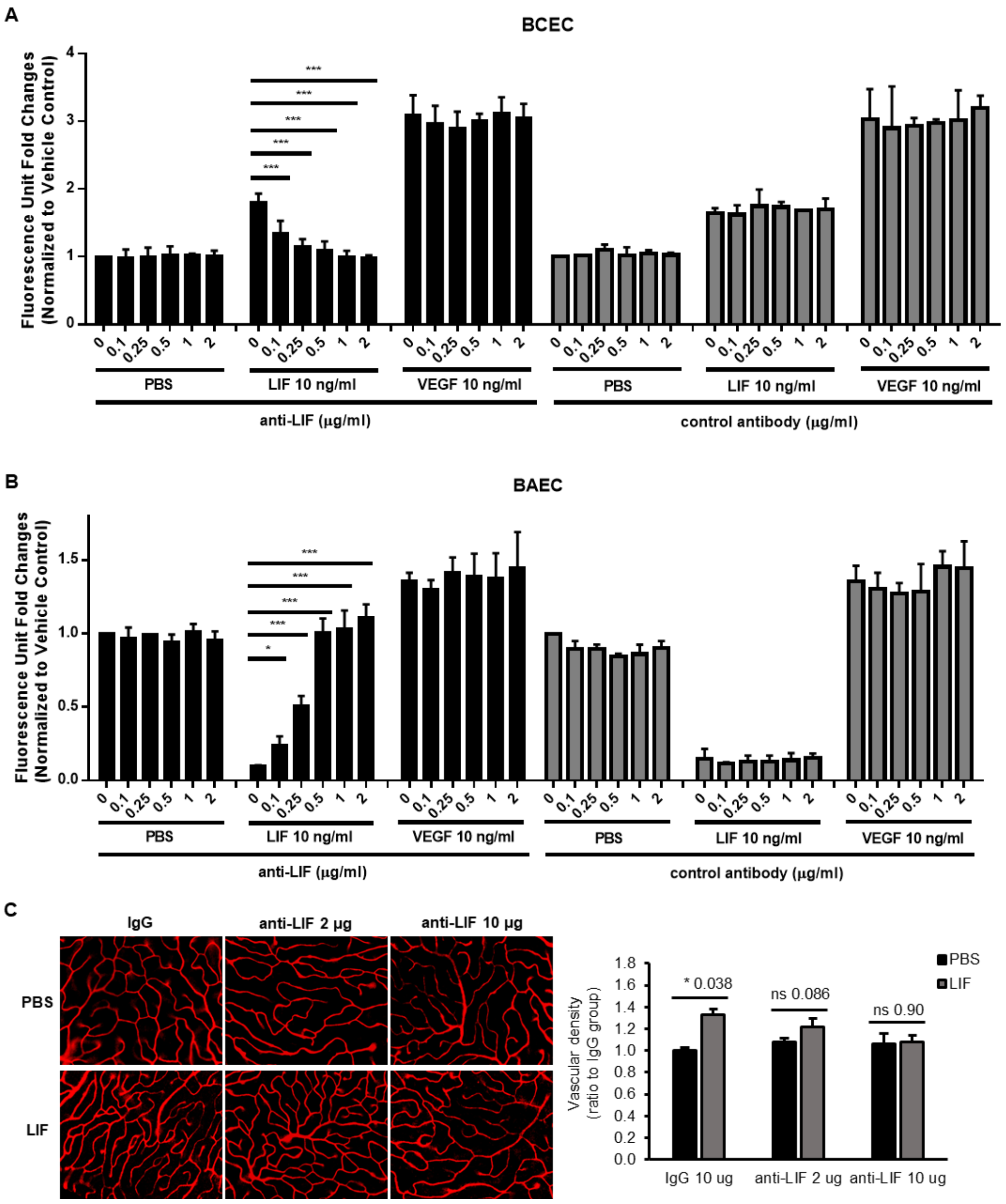

Appendix Figure S14. Anti-LIF antibody treatment abolished the pro-angiogenic effects of LIF *in vitro* and *in vivo*.

A) Different concentrations of anti-LIF antibody (0.1-2 µg/ml) were used to neutralize LIF effects in BCE cells (A) and BAE cells (**B**). Proliferation assays were carried out and VEGF was used as a control. n=3. Three independent experiments were carried out with three technical replicates in each experiment.

C) 2 or 10 µg of anti-LIF antibody was injected in mouse vitreous together with 50 ng of in-house LIF with low-endotoxin levels (see Materials and Methods). IgG2A isotype was used as control. Retinal flat mounts were harvested after 7 days. CD31 was used as a vascular marker. Quantification of vascular density is shown in the right panel, n = 6. Three independent experiments were carried out with six technical replicates in each experiment.

Data information: Bars and error bars represent mean ± SEM. *, $p < 0.05$; ***, $p < 0.001$; ns, not statistically significant. Two-way ANOVA test was used as statistical test.

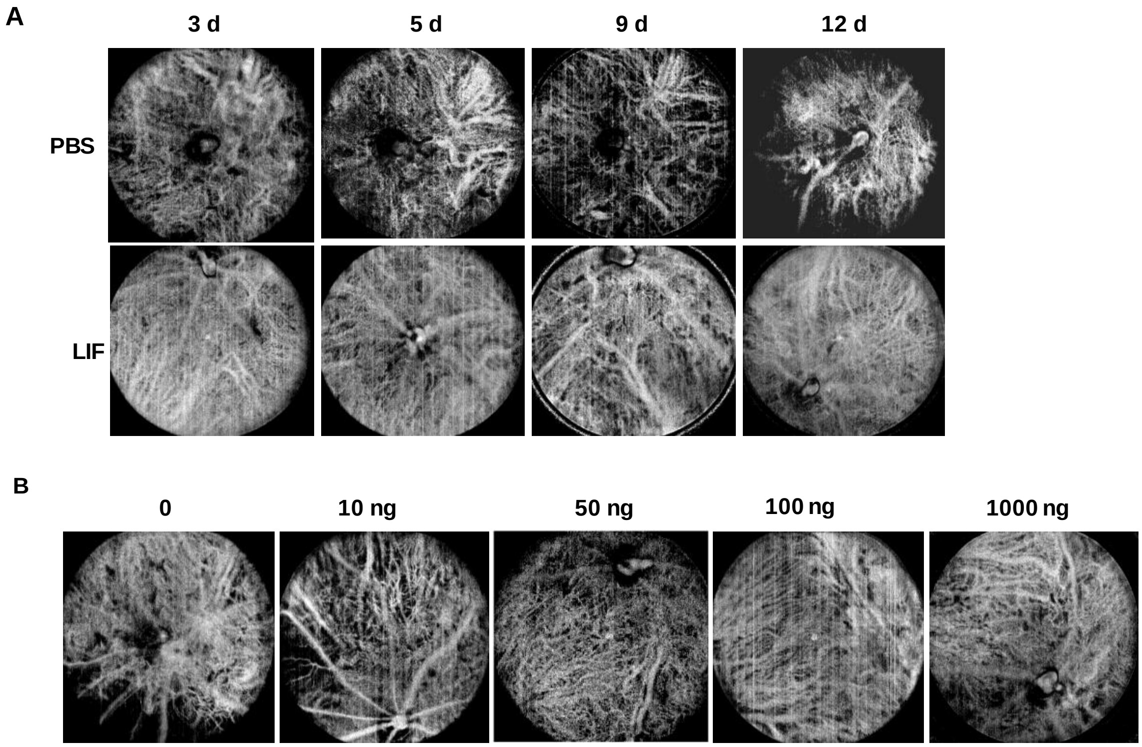

Appendix Figure S15. **Time- and dose-dependent protective effects of LIF in the Na iodate-induced injury model.**

A) After sodium iodate injection, 50 ng LIF or PBS was intravitreally injected and eyes were imaged using OCT-A system at 3d, 5d, 9d, and 12d after injection.

B) After sodium iodate injection, the indicated amounts of LIF were injected in the eyes. PBS was used as vehicle control. Choroidal capillaries were imaged by OCT-A at day 12.

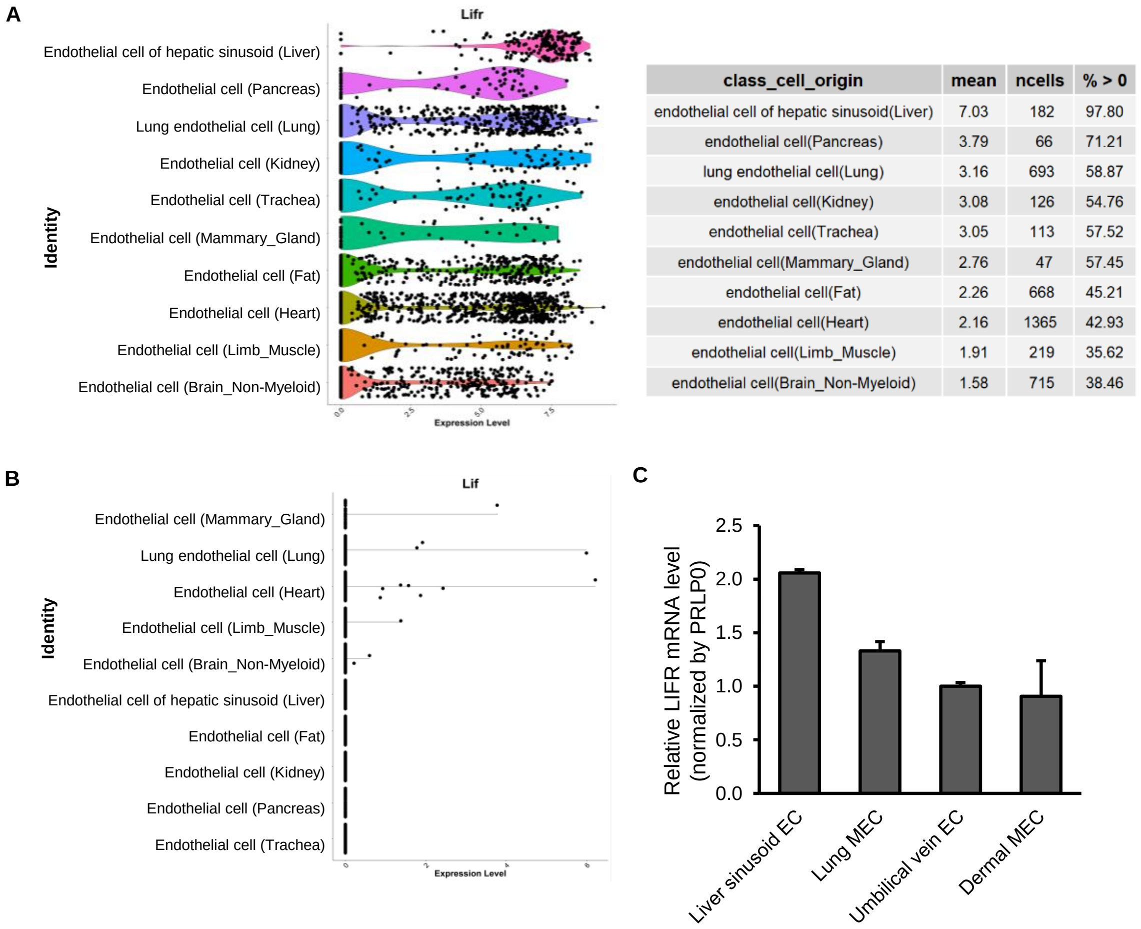

class_cell_origin	mean	ncells	% > 0
endothelial cell of hepatic sinusoid(Liver)	7.03	182	97.80
endothelial cell(Pancreas)	3.79	66	71.21
lung endothelial cell(Lung)	3.16	693	58.87
endothelial cell(Kidney)	3.08	126	54.76
endothelial cell(Trachea)	3.05	113	57.52
endothelial cell(Mammary_Gland)	2.76	47	57.45
endothelial cell(Fat)	2.26	668	45.21
endothelial cell(Heart)	2.16	1365	42.93
endothelial cell(Limb_Muscle)	1.91	219	35.62
endothelial cell(Brain_Non-Myeloid)	1.58	715	38.46

Appendix Figure S16. Differential LIFR expression in mouse and human endothelial cells.

A, B) Violin plots of the log scale normalized expression of LIF receptor (**A**) and LIF (**B**) in EC from different mouse organs, based on *Tabula muris* (Consortium, 2018). Left panel of (A) showed the quantification of LIFR expression in different endothelial cells.

C) qPCR (Taqman) analysis of LIFR expression in different cultured human endothelial cell types. Bars and error bars represent mean ± SD.

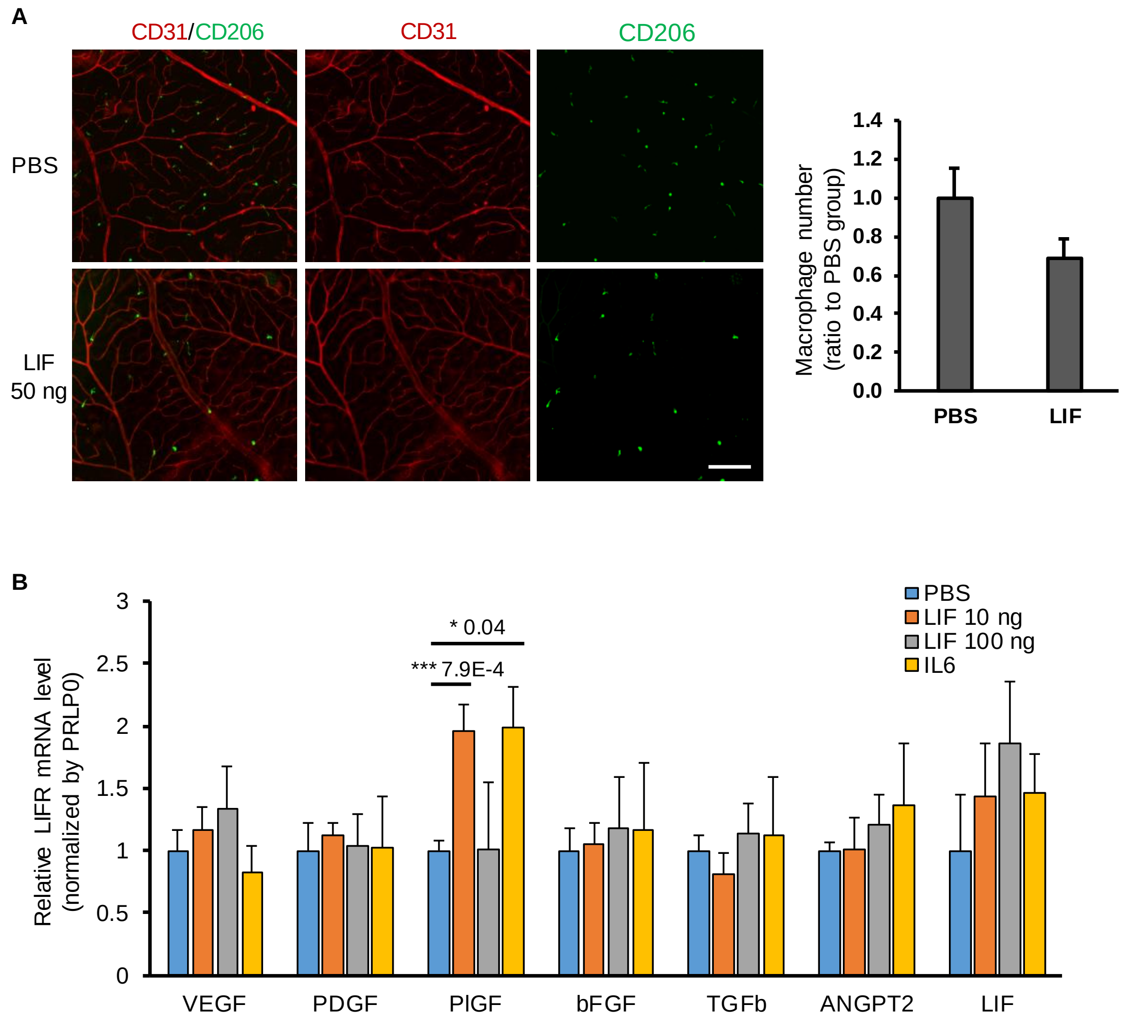

Appendix Figure S17. LIF does not increase macrophage numbers in the retina.

A) 50 ng of in-house LIF with low-endotoxin levels (see Materials and Methods) was injected in the mouse vitreous. Retina flat mounts were harvested and stained after 3 days. CD206 was used as a macrophage marker and CD31 as a vascular endothelial cell marker. Scale bar = 100 µm. Quantification is shown. n = 6. Three independent experiments were carried out with six technical replicates in each experiment.

B) Taqman qPCR analysis shows expression of several angiogenic factors after LIF treatment (16 hours) in mouse peritoneal macrophages.

Data information: Bar graphs and error bars represent mean ± SEM. *, $p < 0.05$; ***, $p < 0.001$ Two-way ANOVA test was used as statistical test.

Gene	Average log fold change	Percentage endothelial	Percentage other	P val adj	KDR correlation
KDR	1.30	53	3	< E-305	1.00
FLT1	2.06	75	12	< E-305	0.36
EDNRB	0.20	29	18	2.8E-44	0.29
ENG	1.85	83	33	< E-305	0.28
ROBO4	0.75	41	2	< E-305	0.26
NRP1	0.60	57	27	1.2E-277	0.26
PLXND1	0.85	57	20	< E-305	0.25
ITGA6	1.20	55	7	< E-305	0.25
LIFR	1.37	71	21	< E-305	0.25
TGFBR2	1.17	72	37	< E-305	0.23
IL6ST	1.43	87	50	< E-305	0.22
FZD4	0.48	31	8	2.2E-250	0.22
TGFBR3	0.90	49	24	5.8E-256	0.10
SELP	1.02	49	2	< E-305	0.10
LEPR	0.07	44	20	5.0E-147	0.06

Appendix Table S1. Endothelial differential expression (log fold increase in endothelial cells), percentage of cells expressing the gene and correlation with KDR of receptors found to cluster with KDR in the correlation matrix of endothelial significant receptors and transcription factors

Consortium, T.M. 2018. Single-cell transcriptomics of 20 mouse organs creates a Tabula Muris. *Nature* 562:367-372.
