# Supplementary figures and images for: LIF, a mitogen for choroidal endothelial cells, protects the choriocapillaris: implications for prevention of geographic atrophy
Source: EMBO Mol Med. 2021 Nov 15;14(1):e14511. doi: 10.15252/emmm.202114511 (PMC8749470; doi:10.15252/emmm.202114511)

## Slide 1
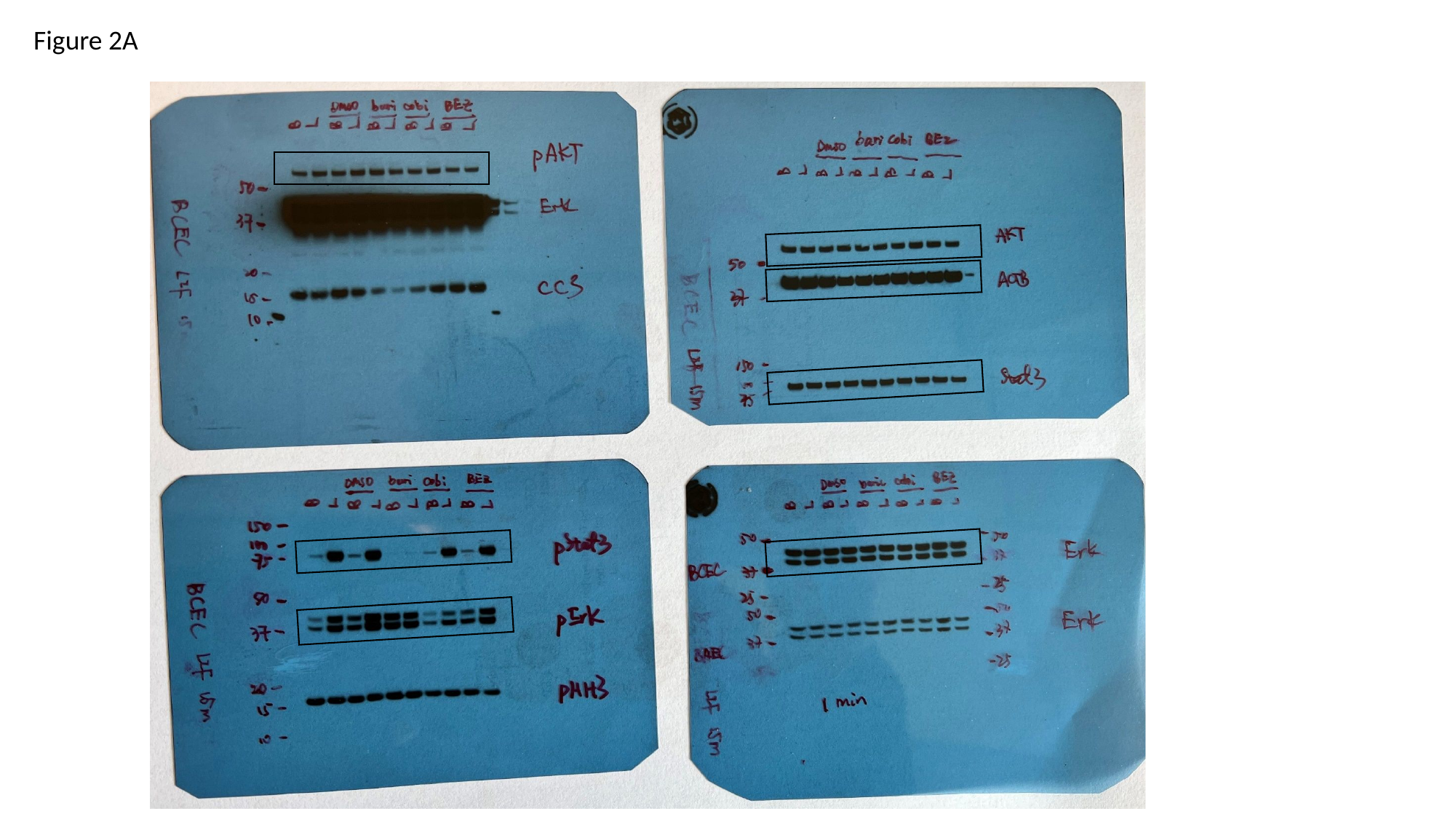

Figure 2A

## Slide 2
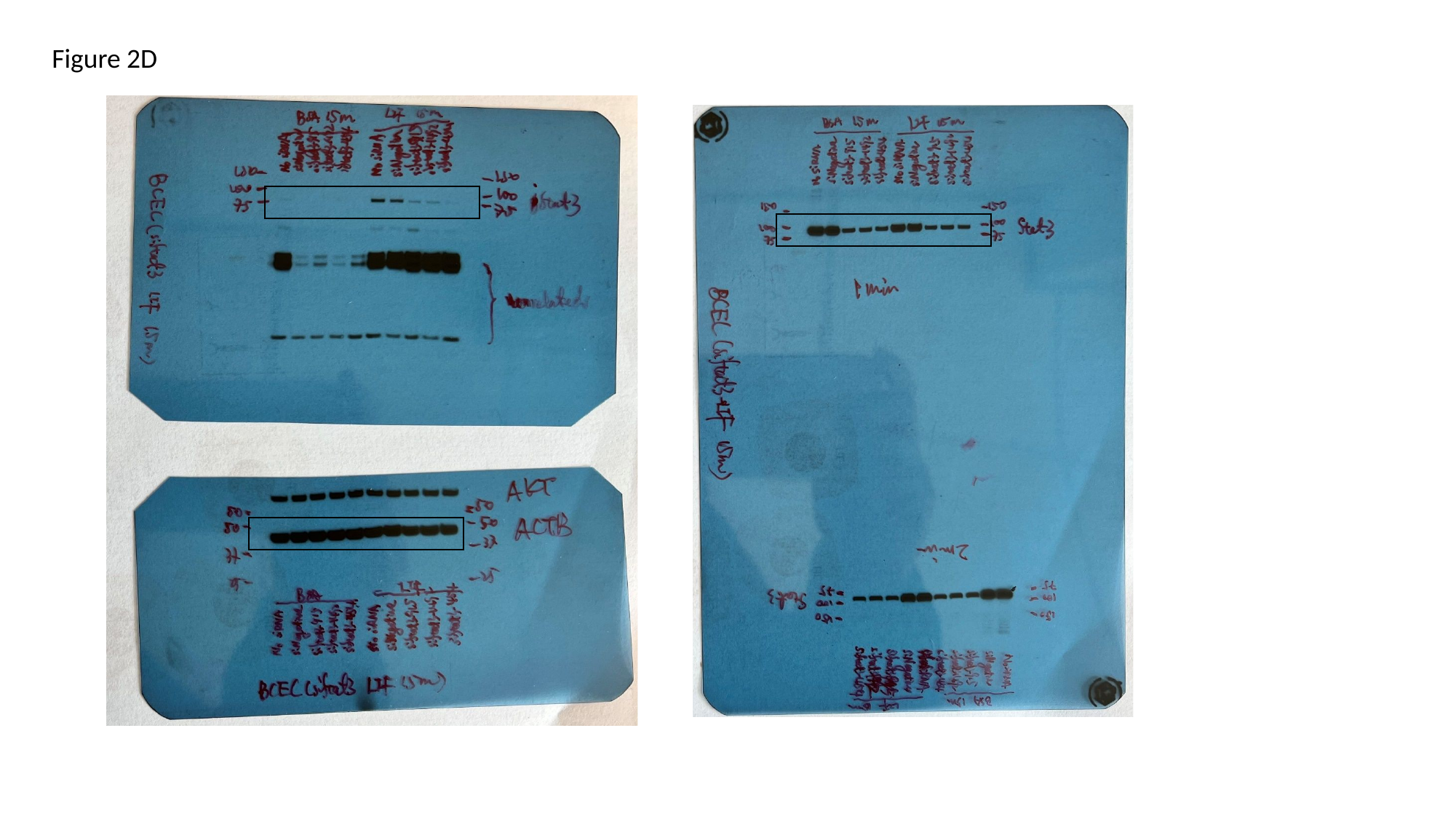

Figure 2D

Supplement: Supplementary file 8 — Source Data for Figure 2 [file EMMM-14-e14511-s012.zip › Original_western_blot_gels_Figure_2.pptx]

## Slide 1
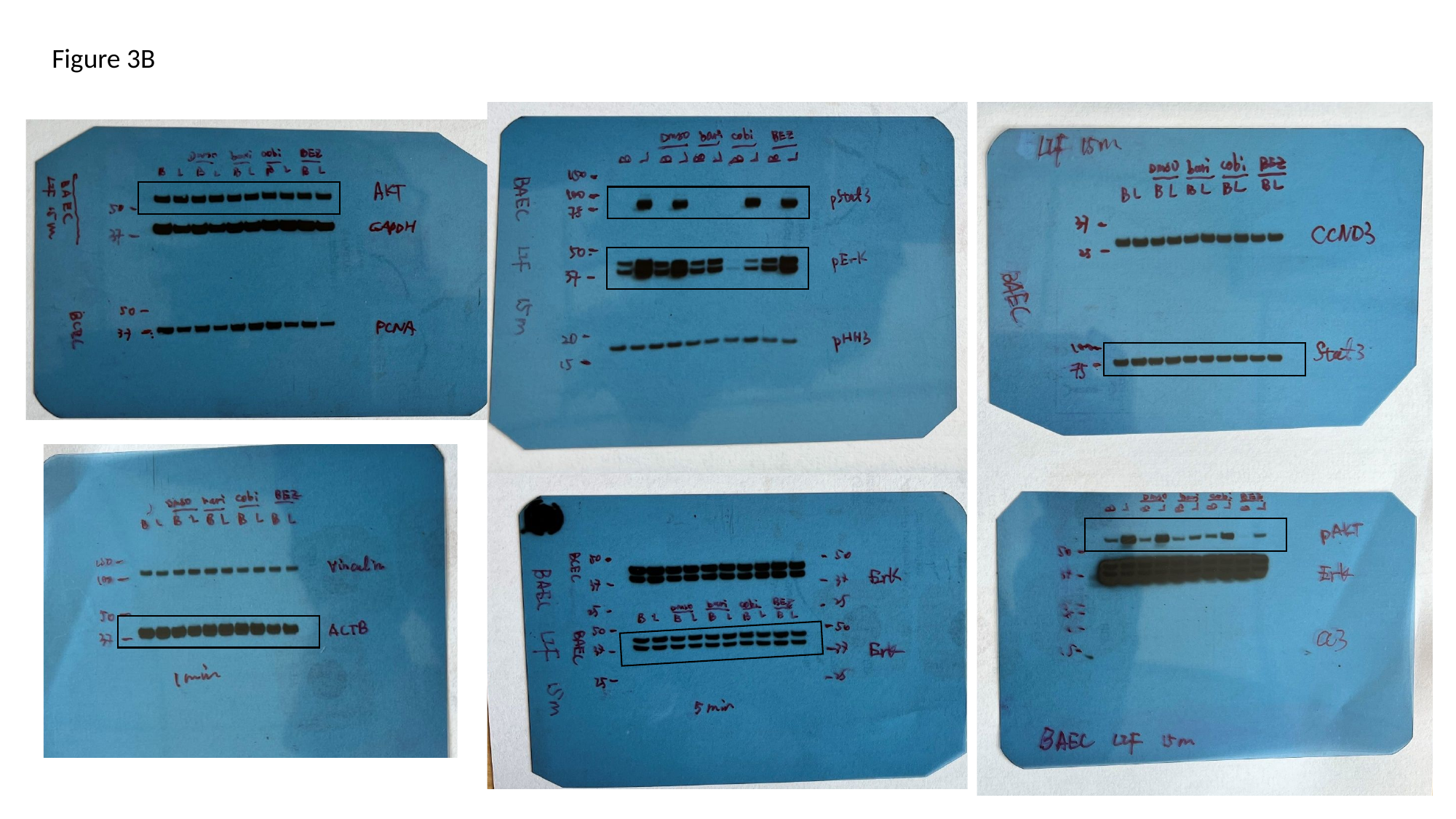

Figure 3B

## Slide 2
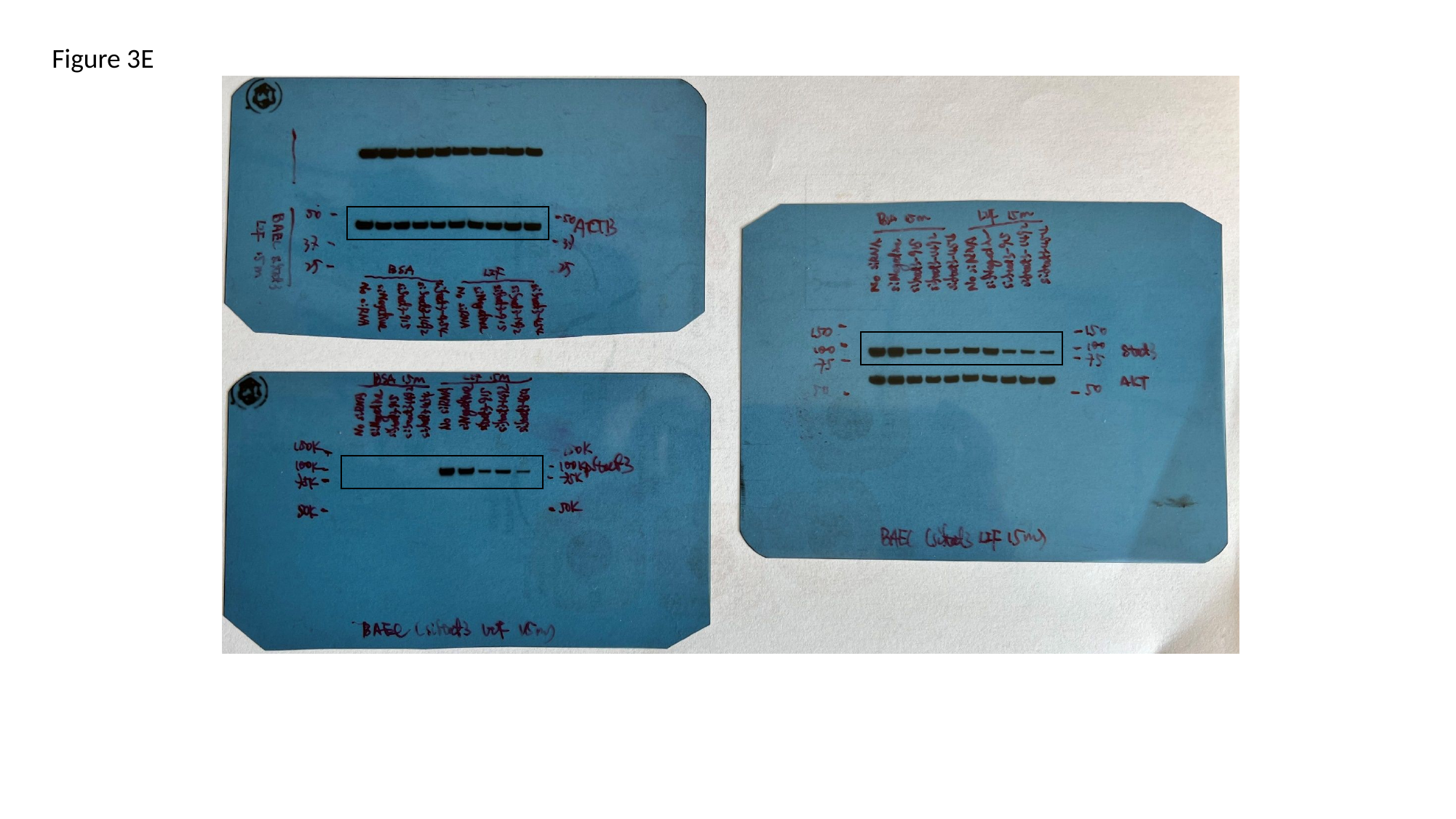

Figure 3E

Supplement: Supplementary file 9 — Source Data for Figure 3 [file EMMM-14-e14511-s007.zip › Original_western_blot_gels_Figure_3.pptx]
