# Supplementary material for: LIF, a mitogen for choroidal endothelial cells, protects the choriocapillaris: implications for prevention of geographic atrophy
Source: EMBO Mol Med. 2021 Nov 15;14(1):e14511. doi: 10.15252/emmm.202114511 (PMC8749470; doi:10.15252/emmm.202114511)
Supplement: Supplementary file 12 [file EMMM-14-e14511-s006.pdf]

EMBO Molecular Medicine

LIF is a mitogen for choroidal EC and protects choriocapillaris from injury: implication for AMD

Pin Li, Qin Li, Nilima Biswas, Hong Xin, Tanja Diemer, Lixian Liu, Lorena Perez Gutierrez, Giovanni Paternostro, Carlo Piermarocchi, Sergii Domanskyi, Ruikang Wang, and Napoleone Ferrara
DOI: 10.15252/emmm.202114511

Corresponding authors: Napoleone Ferrara (nferrara@ucsd.edu)

Review Timeline:

Submission Date:	5th May 21
Editorial Decision:	1st Jun 21
Revision Received:	22nd Sep 21
Editorial Decision:	7th Oct 21
Revision Received:	21st Oct 21
Accepted:	25th Oct 21

Editor: Lise Roth

Transaction Report:

(Note: With the exception of the correction of typographical or spelling errors that could be a source of ambiguity, letters and reports are not edited. Depending on transfer agreements, referee reports obtained elsewhere may or may not be included in this compilation. Referee reports are anonymous unless the Referee chooses to sign their reports.)

1st Editorial Decision 1st Jun 2021

1st Jun 2021

Dear Dr. Ferrara,

Thank you for the submission of your manuscript to EMBO Molecular Medicine. We have now received feedback from the three reviewers who agreed to evaluate your manuscript. As you will see from the reports below, the referees acknowledge the interest of the study and are overall supporting publication of your work pending appropriate revisions.

Addressing the reviewers' concerns in full will be necessary for further considering the manuscript in our journal, and acceptance of the manuscript will entail a second round of review. EMBO Molecular Medicine encourages a single round of revision only and therefore, acceptance or rejection of the manuscript will depend on the completeness of your responses included in the next, final version of the manuscript. For this reason, and to save you from any frustrations in the end, I would strongly advise against returning an incomplete revision.

Revised manuscripts should be submitted within three months of a request for revision; they will otherwise be treated as new submissions, except under exceptional circumstances in which a short extension is obtained from the editor.

When submitting your revised manuscript, please carefully review the instructions that follow below. We perform an initial quality control of all revised manuscripts before re-review; failure to include requested items will delay the evaluation of your revision.

We require:

1) A .docx formatted version of the manuscript text (including legends for main figures, EV figures and tables). Please make sure that the changes are highlighted to be clearly visible.

2) Individual production quality figure files as .eps, .tif, .jpg (one file per figure). For guidance, download the 'Figure Guide PDF' (https://www.embopress.org/page/journal/17574684/authorguide#figureformat).

3) A .docx formatted letter INCLUDING the reviewers' reports and your detailed point-by-point responses to their comments. As part of the EMBO Press transparent editorial process, the point-by-point response is part of the Review Process File (RPF), which will be published alongside your paper.

4) A complete author checklist, which you can download from our author guidelines (https://www.embopress.org/page/journal/17574684/authorguide#submissionofrevisions). Please insert information in the checklist that is also reflected in the manuscript. The completed author checklist will also be part of the RPF.

5) It is mandatory to include a 'Data Availability' section after the Materials and Methods. Before submitting your revision, primary datasets produced in this study need to be deposited in an appropriate public database, and the accession numbers and database listed under 'Data Availability'. Please remember to provide a reviewer password if the datasets are not yet public (see https://www.embopress.org/page/journal/17574684/authorguide#dataavailability).

In case you have no data that requires deposition in a public database, please state so in this section. Note that the Data Availability Section is restricted to new primary data that are part of this study.

6) For data quantification: please specify the name of the statistical test used to generate error bars and P values, the number (n) of independent experiments (specify technical or biological replicates) underlying each data point and the test used to calculate p-values in each figure legend. The figure legends should contain a basic description of n, P and the test applied. Graphs must include a description of the bars and the error bars (s.d., s.e.m.).

7) We would also encourage you to include the source data for figure panels that show essential data. Numerical data should be provided as individual .xls or .csv files (including a tab describing the data). For blots or microscopy, uncropped images should be submitted (using a zip archive if multiple images need to be supplied for one panel). Additional information on source data and instruction on how to label the files are available at
.

8) Our journal encourages inclusion of *data citations in the reference list* to directly cite datasets that were re-used and obtained from public databases. Data citations in the article text are distinct from normal bibliographical citations and should directly link to the database records from which the data can be accessed. In the main text, data citations are formatted as follows: "Data ref: Smith et al, 2001" or "Data ref: NCBI Sequence Read Archive PRJNA342805, 2017". In the Reference list, data citations must be labeled with "[DATASET]". A data reference must provide the database name, accession number/identifiers and a resolvable link to the landing page from which the data can be accessed at the end of the reference. Further instructions are available at .

9) We replaced Supplementary Information with Expanded View (EV) Figures and Tables that are collapsible/expandable online. A maximum of 5 EV Figures can be typeset. EV Figures should be cited as 'Figure EV1, Figure EV2" etc... in the text and their respective legends should be included in the main text after the legends of regular figures.

- For the figures that you do NOT wish to display as Expanded View figures, they should be bundled together with their legends in a single PDF file called *Appendix*, which should start with a short Table of Content. Appendix figures should be referred to in the main text as: "Appendix Figure S1, Appendix Figure S2" etc.
- Additional Tables/Datasets should be labeled and referred to as Table EV1, Dataset EV1, etc. Legends have to be provided in a separate tab in case of .xls files. Alternatively, the legend can be supplied as a separate text file (README) and zipped together with the Table/Dataset file.

See detailed instructions here:
.

10) The paper explained: EMBO Molecular Medicine articles are accompanied by a summary of the articles to emphasize the major findings in the paper and their medical implications for the non-specialist reader. Please provide a draft summary of your article highlighting
- the medical issue you are addressing,
- the results obtained and
- their clinical impact.

This may be edited to ensure that readers understand the significance and context of the research. Please refer to any of our published articles for an example.

11) For more information: There is space at the end of each article to list relevant web links for further consultation by our readers. Could you identify some relevant ones and provide such information as well? Some examples are patient associations, relevant databases, OMIM/proteins/genes links, author's websites, etc...

12) A Conflict of Interest statement should be provided in the main text.

13) Every published paper now includes a 'Synopsis' to further enhance discoverability. Synopses are displayed on the journal webpage and are freely accessible to all readers. They include a short stand first (maximum of 300 characters, including space) as well as 2-5 one-sentences bullet points that summarizes the paper. Please write the bullet points to summarize the key NEW findings. They should be designed to be complementary to the abstract - i.e. not repeat the same text. We encourage inclusion of key acronyms and quantitative information (maximum of 30 words / bullet point). Please use the passive voice. Please attach these in a separate file or send them by email, we will incorporate them accordingly.

Please also suggest a striking image or visual abstract to illustrate your article as a PNG file 550 px wide x 300-600 px high.

EMBO Molecular Medicine has a "scooping protection" policy, whereby similar findings that are published by others during review or revision are not a criterion for rejection. Should you decide to submit a revised version, I do ask that you get in touch after three months if you have not completed it, to update us on the status.

Looking forward to receiving your revised manuscript,

With kind regards,

Lise Roth

Lise Roth, PhD
Editor
EMBO Molecular Medicine

***** Reviewer's comments *****

Referee #1 (Comments on Novelty/Model System for Author):

The paper investigates an interesting mediator of angiogenesis and provides conceptual insight on how LIF can be used in AMD. The experiments are well conducted and conclusions general backed. While interesting, it is not immediately clear why the authors make claims on dry AMD (especially in the abstract) given that there is no relevant data on this form of AMD.

Referee #1 (Remarks for Author):

Li and colleagues introduce the pro-angiogenic role of LIF in choroidal endothelial cells using diverse endothelial cell models in vitro. They propose that LIF could be of clinical interest for AMD and performed experiments using laser induced-choroidal neovascularization and NaIO3-induced retina atrophy and evaluating the retinal or choroidal vasculature. Using meta-analytic approaches, they confirmed that the endothelial cells in the human choriocapillaries are enriched in LIFR. The study concludes that given its mitogenic potential, LIF is of therapeutic interest in AMD.

General comment:
The paper investigates an interesting mediator of angiogenesis and provides conceptual insight on how LIF can be used in AMD. The experiments are well conducted and conclusions general backed.While interesting, it is not immediately clear why the authors make claims on dry AMD (especially in the abstract) given that there is no relevant data on this form of AMD.

Specific comments:

1. In the introduction, please add reference for 'In search of novel regulators of choroidal EC growth/survival, we screened human glioblastoma (GBM) cell lines since tumors have been historically proven to be a rich source of a variety of bioactive factors.'

2. State the rationale to use this glioblastoma cell line and not others? This is an important question given the "unbiased" approach of the hypothesis.

3. With regards to therapeutic interventions, how was the working concentration for anti-LIF established in vitro?

4. Folds of increase of cells subjected to LIF and VEGFA stimulation has quite variable ranges from cell type to cell type (see FigS1). Are LIF receptors differentially expressed in the ECs tested?

5. Fig. 1: Indicate cell type in each graph. Indicate for what stand R26 and R27 (high mitogenic fractions)

6. I would suggest putting Fig. S1E in the main figure (Fig. 1) as it constitutes part of the rationale of the work.

7. Fig. S2 in general: indicate which cell type is each graph since some are bovine, some human...

8. Fig. 2A: How were the concentrations determined for Ba, Co and BE?

9. Fig. 3A: Authors should include a group using VEGF stimulation as a proliferation control.

10. The sentence 'Interestingly, cathepsin L mRNA (CTSL) expression in BCE cells was undetectable by qRT-PCR' is written twice in the result section. Since RNAseq experiment was also performed, what was CTSL expression in RNAseq rather only than by RT-qPCR

11. Fig. 4A: The vasculature in the flat-mounts displayed is not visible

12. Fig. S7B: The difference between LIF and VEGF phenotype is interesting, however in Fig 1F the authors showed that VEGF and LIF can have synergistic effect. It would be interesting to know if LIF could worsening leakage in diseased retina. What would be the effect of VEGF + LIF injection (or LIF injection in OIR model?) Did the authors quantified leakage?

13. Figure 5A AMD and CNV model are both highly dependent of inflammation. Does LIF has an effect on inflammatory cells (that could have paracrine angiogenic activity) or is strictly on ECs?

14. In the discussion authors wrote 'The finding that these cytokines have protective effects in the NaIO3 model suggests that they may have therapeutic value in protecting the CC and thus may prevent atrophy in AMD'. However, the laser-induced CNV model demonstrate that LIF increase pathological neovascularization. Theorically, using LIF as a treatment for early AMD could be protective for dry AMD progression, but may also drive the progression through sight-threating wet AMD (notably in a context of elevated level of VEGF, see Fig 1F)

Minor Points:

-State explicitly in the results section that LN229 is a glioblastoma cell line the first time that mentioned.

-In some parts of the manuscript I found some words underlined (?) or in different font color. Also follow the same style to name numbers numerically or by wording.

-In the section 'Expression of LIFR in retinal and choroidal cells by IHC' please introduce IL6ST before the sentence 'Treatment of human choroid EC and mouse retinal EC with LIF also resulted in STAT3 activation as assessed by a robust phosphorylation signal, indicating the presence of LIFR and IL6ST in human and mouse retinal EC (Fig. 6G)'.

-Methods should clearly indicate how was performed the quantification of retina vasculature (flat-mounts and OCT) in ImageJ for reproducibility purposes.

-In reagents section: Do human LIF (Sigma, CAT# SRP9001) and human LIF (Biolegend, CAT# 593902) give similar results?

-In 'Statistical analysis' section, it is not clear if statistical tests are performed on technical replicates within one experiment or with the means of technical replicates from each independent experiment.

-Please add reference for 'IL-8 lacks mitogenic effects on BCE cells.'

Referee #2 (Comments on Novelty/Model System for Author):

The distinct angiogenic responses of BCE, BRE vs BCE to LIF stimulation are very interesting. The reasons behind the opposite effects of LIF on retinal angiogenesis observed in this study and previous reports need to be further strengthened.

LIF/STAT3 signaling pathways have been well documented in ECs, RPE/retinas, which may dampen the novelty.

Referee #2 (Remarks for Author):

Li et al investigated the angiogenic impacts of leukemia inhibitory factor (LIF) on ocular vessels. The authors found that 1) distinct angiogenic responses of BCE, BRE vs BAE, identified with in vitro models and RNA-seq. 2) LIF stimulated choroidal and retinal angiogenesis via JAK-STAT3 pathways both in vitro and in vivo. More interestingly, LIF treatment decreased $NaIO_3$-induced avascular area and preserved retinal thickness, suggesting a potential therapeutic impact on dry AMD and geographic atrophy. Overall, the study is well-designed and carefully conducted. However, there are several concerns to be further addressed

1. Figure 2, 3: please also include the quantification of western blots (n number is at least 3).

2. Figure 4, intravitreal LIF delivery promoted retinal angiogenesis in adult and neonatal mice. However, in LIFKO and LIF-overexpressing mice, LIF have demonstrated inhibitory effects on retinal angiogenesis. The authors mentioned that it might due to LIF modulation of retinal astrocytic template. In the study by Sakimoto et al, intravitreal LIF at P3 APJ KO mouse retinas (with dense astrocytic network) prevented the overgrowth of immature astrocytes and hyperproliferation of retinal vessels 48 hours after LIF delivery. In the current manuscript, intravitreal LIF was delivered at P5 WT mouse retinas when the astrocyte network is established and is undergoing maturation. The authors shall also evaluate the impacts of LIF on P3 WT mouse retinas and see if LIF inhibition of retinal angiogenesis are observed as in the studies of LIFKO, LIF-overexpressing mice, as well as in APJ KO mice, before drawing the conclusion that the distinct roles of LIF in retinal angiogenesis is due to the different developmental stages.

3. Figure 5: please include the n number for Fig 5A. As there is usually a big variation in the laser-induced CNV model (PMID: 24136346), please include at least 6 mice per group in the study. Both genders shall be used. The graph is recommended to be shown in dot format instead of bar format.

4. Figure S10, LIF was used at 0, 10, 100, and 1000ng. However, the authors stated that "In the $NaIO_3$ model, LIF effects were dose-dependent from 10ng and the effect was saturated at 50ng" in the Result session. The authors please confirm.

5. Although the current study focused on LIF's angiogenic impacts, LIF has also been reported to change visual cycle genes and proteins in retina and RPE (PMID: 22645143). Would this be a concern when use LIF to protect choroidal vessels? The authors please comment.

6. As LIF has demonstrated inhibitory effects on BAE angiogenesis, would intravitreal LIF delivery lead to potential systemic side effects? There have been systemic concerns raised when use intraocular anti-VEGF therapy in retinal diseases. The authors please comment.

Referee #3 (Comments on Novelty/Model System for Author):

The medical relevance is high given that AMD still is the leading cause of blindness. The use of mouse models to study this is adequate.

Referee #3 (Remarks for Author):

N. Ferrara report interesting new findings about LIF acting on endothelial cells. They show that LIF inhibits EC proliferation as reported before. In addition, they unraveled a new mechanism how LIF can induce endothelial cell death. In endothelial cells in the mouse retina, LIF however leads to the opposite effect stimulating endothelial proliferation and vessel growth. Importantly, this was associated with better outcome in several eye disease models in mice. In contrast to VEGF, LIF treatment did not lead to vessel leakage. While the findings or very interesting (e.g. organ-specific endothelial cell functions) and have translational potential, this study needs substantial revision.

Major points:

The authors should determine absolute concentrations of LIF in the conditioned medium from LN-229 cells.

The strong differences in endothelial cell response to LIF is intriguing. This study would gain substantial impact if the authors could show this not only in BCE and BAE cells but also in endothelial cells derived from other organs.

Could it be that the differential endothelial cell response towards LIF is due to different expression of LIF receptors in organ-specific vascular beds? The authors may use of their RNAseq data sets to have a first look into this. In addition, the authors could block LIF receptors to determine whether these molecules indeed transmit the observed effects. Moreover, single cell RNAseq data bases of organ-specific endothelial cells should be used to determine LIF receptor expression patterns (as already done partially by the authors suppl Table 4).

Does LIF affect in particular fenestrated capillary endothelial cells?

The study lacks explanation why 10 to 100 ng LIF was injected into mouse eyes. What is the rationale for using this dose?

The in vitro experiments indicate that LIF acts directly on endothelial cells. The authors however also show LIF receptor expression in other cell types within the retina. This raises the question whether the observed effects upon intravitreal LIF injection are indeed mediated through LIF receptors on endothelial cells or by an unknown paracrine factor released from e.g. pericytes. Although this issue is difficult to address (cell type specific LIFR knockout mice would be ideal) the authors could try to stain tissue for LIF downstream signaling.

An important information lacking in this study is whether the pro-aniogenic effect of LIF in the eye is transient or durable. In addition, are the new blood vessels covered by pericytes in a similar degree as those in the control group?

The authors show a very interesting kind of cell death upon LIF treatment in BAE cells. However, it remained unclear whether this is only observed in this particular cell type under cell culture conditions or whether this would also occur in mice. This is also an important question when thinking about translation into clinical application.

Several figures should be improved. For example Fig. 7 has very low resolution making it difficult to read. Also p values should be indicated for all statistical analyses.

EMM-2021-14511

Response to the Reviewers

Referee #1 (Comments on Novelty/Model System for Author):

The paper investigates an interesting mediator of angiogenesis and provides conceptual insight on how LIF can be used in AMD. The experiments are well conducted and conclusions general backed. While interesting, it is not immediately clear why the authors make claims on dry AMD (especially in the abstract) given that there is no relevant data on this form of AMD.

Referee #1 (Remarks for Author):

Li and colleagues introduce the pro-angiogenic role of LIF in choroidal endothelial cells using diverse endothelial cell models in vitro. They propose that LIF could be of clinical interest for AMD and performed experiments using laser induced-choroidal neovascularization and NaIO3-induced retina atrophy and evaluating the retinal or choroidal vasculature. Using meta-analytic approaches, they confirmed that the endothelial cells in the human choriocapillaries are enriched in LIFR. The study concludes that given its mitogenic potential, LIF is of therapeutic interest in AMD.

Thank you for your comments

General comment:
The paper investigates an interesting mediator of angiogenesis and provides conceptual insight on how LIF can be used in AMD. The experiments are well conducted and conclusions general backed.While interesting, it is not immediately clear why the authors make claims on dry AMD (especially in the abstract) given that there is no relevant data on this form of AMD.

We thank the reviewer for these comments. It is difficult to model dry AMD in animals. However, the Na iodate model is believed to reflect damage from oxidative stress, which is thought to be a key feature of GA in dry AMD (PMID 4316465).

Specific comments:

1. In the introduction, please add reference for 'In search of novel regulators of choroidal EC growth/survival, we screened human glioblastoma (GBM) cell lines since tumors have been historically proven to be a rich source of a variety of bioactive factors.'

We added a reference.

2. State the rationale to use this glioblastoma cell line and not others? This is an important question given the "unbiased" approach of the hypothesis.

The goal of our study was identifying novel regulators of EC growth. Tumors such as GBM have been shown in many studies to produce a variety of growth factors, including endothelial mitogens. We chose the LN-229 cell line, primarily because of its unusual characteristics: very low expression of VEGF (PMID: 27470974) and absence of other angiogenic factors in the medium (Fig. S1B). Nevertheless, LN-229 cells have been reported to form invasive tumors (PMID: 27470974). We found that the conditioned

medium had robust mitogenic effects on endothelial cells. Thus, LN-229 cells appeared to be a very intriguing model to identify mitogens, with the appropriate EC as a bioassay.

3. With regards to therapeutic interventions, how was the working concentration for anti-LIF established in vitro?

As shown in Fig. 1D, an anti-LIF antibody was used in early studies to determine whether the mitogenic effects of reverse-phase HPLC fractions, R26 and R27, from LN-229 conditioned medium were truly mediated by LIF, as suggested by the mass spectrometry data. We tested a commercial polyclonal anti-LIF antibody and a control antibody at concentrations based on the nd50 value indicated in the manufacturer's datasheet. We tested a concentration 10-fold higher than the nd50 in order to fully neutralize the LIF in the conditioned medium. Fig. 1D illustrates the effects of 20 μg/ml antibody that neutralized the mitogenic effects of the fractions,but had no effects on VEGF.

4. Folds of increase of cells subjected to LIF and VEGFA stimulation has quite variable ranges from cell type to cell type (see FigS1). Are LIF receptors differentially expressed in the ECs tested?

Indeed, the fold increase under LIF is quite variable. This is not entirely unexpected, considering that the fold increases in BCE induced by VEGF also shows significant variability, ranging from 2 to 6 fold. We tested the expression of LIFR in different ECs. The expression level varied among different cell lines, but it is still in a relatively similar range (Fig. S16C). The variability in proliferation may also be due to cell passage tested.

5. Fig. 1: Indicate cell type in each graph. Indicate for what stand R26 and R27 (high mitogenic fractions)

We labeled cell types in the graph. R26 and R27 indicate fraction number from the reverse-phase chromatography.

6. I would suggest putting Fig. S1E in the main figure (Fig. 1) as it constitutes part of the rationale of the work.

We appreciate the suggestion. We moved Fig. S1E to main Fig.1.

7. Fig. S2 in general: indicate which cell type is each graph since some are bovine, some human...

We indicated the cell type on the graph.

8. Fig. 2A: How were the concentrations determined for Ba, Co and BE?

The concentrations used are consistent with previous studies using the same inhibitors. References have been added in the text. Our western blot data show that, at the concentrations tested, phosphor-stat3, pERK or pAkt were efficiently inhibited (Fig. 2A and 3A).

9. Fig. 3A: Authors should include a group using VEGF stimulation as a proliferation control.

We added a group testing VEGF in BAE cell proliferation.

10. The sentence 'Interestingly, cathepsin L mRNA (CTSL) expression in BCE cells was undetectable by qRT-PCR' is written twice in the result section. Since RNAseq experiment was also performed, what was CTSL expression in RNAseq rather only than by RT-qPCR

Thank you for pointing this out. We deleted the repeated sentences.

Cathepsin L mRNA was not detected by RNAseq in both BCE and BAE cells. However, the samples for RNA seq were harvested 6 hrs after LIF treatment, while the RNA for RT-qPCR were prepared after 24 hrs. Indeed, according to previous studies in different settings, such as mammary gland involution, upregulation of cathepsins begins 12 hours after STAT3 activation (PMID: 21336304).

11. Fig. 4A: The vasculature in the flat mounts displayed is not visible

The images have been replaced. Higher magnification images are now shown.

12. Fig. S7B: The difference between LIF and VEGF phenotype is interesting, however in Fig 1F the authors showed that VEGF and LIF can have synergistic effect. It would be interesting to know if LIF could worsening leakage in diseased retina. What would be the effect of VEGF + LIF injection (or LIF injection in OIR model?) Did the authors quantify leakage?

This is an important question. We injected LIF plus VEGF intravitreally and quantified the leakage area using the ratio of FITC-lectin area to TRITC-dextran area. Our results show that the retinal leakage induced by the LIF/VEGF combination was similar to VEGF alone, suggesting LIF did not worsen VEGF-induced retinal leakage. We replaced the previous images as Fig. S12B with this new image and quantification.

13. Figure 5A AMD and CNV model are both highly dependent of inflammation. Does LIF has an effect on inflammatory cells (that could have paracrine angiogenic activity) or is strictly on ECs?

While LIF is a member of the IL-6 family, several studies have shown surprising anti-inflammatory effects of LIF in different contexts (PMID: 6793494). Single cell transcriptomic studies in human choroid show that the specific IL-6 receptor (IL6R) is highly expressed in cells of the macrophage/monocyte lineage (Fig. 7D, while LIFR has little expression in these cells and has much higher expression in EC (Fig. 7B).

We show in this revision that intravitreal injection of LIF does not result in increase in the number of macrophages in the retina relative to control, arguing against pro-inflammatory effects of LIF (Fig. S17A). We also performed studies on isolated mouse peritoneal macrophages. After incubation with LIF, we tested the gene expression of several pro-angiogenic factors: VEGF, PDGF, bFGF, PlGF, TGFβ, angiopoietin-2 and LIF itself. However, only PlGF was slightly upregulated (1.9 fold) (Fig. S17B).

Another experiment that argues for a direct LIF effect on EC is the finding that, two hours after LIF injection, there was a clear increase in STAT3 phosphorylation in endothelial cells, based on CD31 IHC (Fig. 4E). The rapidity of this response argues for a direct effect of LIF on endothelial cells, rather than a paracrine mechanism. Please see also our response to reviewer # 3 on this point.

14. In the discussion authors wrote 'The finding that these cytokines have protective effects in the NaIO3 model suggests that they may have therapeutic value in protecting the CC and thus may prevent atrophy in AMD'. However, the laser-induced CNV model demonstrate that LIF increase pathological neovascularization. Theorically, using LIF as a treatment for early AMD could be protective for dry AMD progression, but may also drive the progression through sight-threating wet AMD (notably in a context of elevated level of VEGF, see Fig 1F)

These are truly excellent comments, but it is difficult to fully address them in animal models, given also the much shorter time window of events in rodents compared to humans: days or weeks vs. years. In the laser-induced CNV model, intense neovascular growth, which is known to be largely VEGF-dependent, occurs very rapidly after the injury, but is short-lived.

Interestingly, recent clinical studies show that the progression of GA is halted in proximity of neovascular areas, likely due to improved perfusion. These provocative studies raise the possibility that a pro-angiogenic treatment may have benefits in this setting, However, this carries the risks pointed out by the reviewer. We believe that protecting the CC from oxidative or other damage during the pre-vascular phase of AMD, which is associated with low VEGF levels, would be a more realistic goal. Needless to say, timing of the treatment, patient selection and monitoring would be critical.

Minor Points:

-State explicitly in the results section that LN229 is a glioblastoma cell line the first time that mentioned.
Done

-In some parts of the manuscript I found some words underlined (?) or in different font color. Also follow the same style to name numbers numerically or by wording.

Thanks. We checked and formatted the manuscript.

-In the section 'Expression of LIFR in retinal and choroidal cells by IHC' please introduce IL6ST before the sentence 'Treatment of human choroid EC and mouse retinal EC with LIF also resulted in STAT3 activation as assessed by a robust phosphorylation signal, indicating the presence of LIFR and IL6ST in human and mouse retinal EC (Fig. 6G)'.

As recommended, we have introduced the definition of IL6ST (GP130), before the sentence.

-Methods should clearly indicate how was performed the quantification of retina vasculature (flat-mounts and OCT) in ImageJ for reproducibility purposes.

We added a method section: quantification of retinal vasculature
Quantification of vasculature was carried out by software ImageJ. The threshold of vascular signal was selected, and vessel-covered area was calculated by Analyze the Particle tools. Vascular area was normalized with the control group.

-In reagents section: Do human LIF (Sigma, CAT# SRP9001) and human LIF (Biolegend, CAT# 593902) give similar results?

Thanks for the question. The Sigma and Biolegend proteins gave very similar results, but we should clarify that the Biolegend molecule was used only for in vitro studies, while Sigma LIF was used for in vitro and in vivo studies. This has been indicated in this revision.

We are aware that the purity of recombinant proteins and in particular the endotoxin (LPS) levels may be a major source of variability, especially for in vivo studies. The LPS conc. in the Sigma LIF is reported to be <0.1 EU/µg protein, a level that should be adequate, especially considering the low amounts of LIF injected in our experiments. To further address this issue, we produced in our lab recombinant LIF, with LPS levels of ≤ 0.2 EU/mg of protein. These levels are >100 fold lower than those we measured in the

Sigma LIF protein. BCE cell proliferation and retinal angiogenesis induced by our in-house LIF were similar to those induced by Sigma LIF.

In this revision, we also show a new control experiment: co-injection with an anti-LIF monoclonal antibody completely blocked LIF-induced retinal angiogenesis (Fig. S14).

-In 'Statistical analysis' section, it is not clear if statistical tests are performed on technical replicates within one experiment or with the means of technical replicates from each independent experiment.

Our statistical tests were performed on technical replicates within one representative experiment. We have at least three independent experiments that have the similar statistical test result.

-Please add reference for 'IL-8 lacks mitogenic effects on BCE cells.'

We have mentioned that, in pilot experiments, IL-8 had no mitogenic activity on BCE cells.

<u>Referee #2</u> (Comments on Novelty/Model System for Author):

The distinct angiogenic responses of BCE, BRE vs BCE to LIF stimulation are very interesting. The reasons behind the opposite effects of LIF on retinal angiogenesis observed in this study and previous reports need to be further strengthened.

Thank you for your comments

We believe that differences in experimental model systems, dosing regimens and probably also in the quality/purity of the reagents likely underlie the conflicting reports in the literature. We have made these points in the discussion. Please see also our response to reviewer #1 (end of p4, beginning of p5 in this letter).

We provide in this revision considerable new data strengthening our conclusion, including the demonstration that co-injecting a neutralizing anti-LIF monoclonal antibody blocks LIF-induced retinal angiogenesis (Fig. S14)

LIF/STAT3 signaling pathways have been well documented in ECs, RPE/retinas, which may dampen the novelty.

We certainly agree that other studies have examined LIF effects in retina and RPE. However, we present here a new perspective of LIF action/biological effects in ocular blood vessels. We show that, depending on EC-type specific STAT3 signaling, very different biological outcomes can be obtained. It has been previously reported that the LIF receptor (LIFR) is expressed only in non-endothelial cells in the retina, while LIF is produced by EC (PMID:2398738). Our data provide clear evidence, including rapid STAT3 phosphorylation in retinal EC following LIF injection (Fig 4E), of direct LIF effects. Our conclusions are entirely support by available single-cell transcriptomic data showing that, while LIF is rarely expressed, LIFR is strongly expressed in EC (Fig 7).

Referee #2 (Remarks for Author):

Li et al investigated the angiogenic impacts of leukemia inhibitory factor (LIF) on ocular vessels. The

authors found that 1) distinct angiogenic responses of BCE, BRE vs BAE, identified with in vitro models and RNA-seq. 2) LIF stimulated choroidal and retinal angiogenesis via JAK-STAT3 pathways both in vitro and in vivo. More interestingly, LIF treatment decreased NaIO3-induced avascular area and preserved retinal thickness, suggesting a potential therapeutic impact on dry AMD and geographic atrophy. Overall, the study is well-designed and carefully conducted. However, there are several concerns to be further addressed

1. Figure 2, 3: please also include the quantification of western blots (n number is at least 3).

We added quantification of western blots from three independent experiments and indicated the values in Figure 2 and 3.

2. Figure 4, intravitreal LIF delivery promoted retinal angiogenesis in adult and neonatal mice. However, in LIFKO and LIF-overexpressing mice, LIF have demonstrated inhibitory effects on retinal angiogenesis. The authors mentioned that it might due to LIF modulation of retinal astrocytic template. In the study by Sakimoto et al, intravitreal LIF at P3 APJ KO mouse retinas (with dense astrocytic network) prevented the overgrowth of immature astrocytes and hyperproliferation of retinal vessels 48 hours after LIF delivery. In the current manuscript, intravitreal LIF was delivered at P5 WT mouse retinas when the astrocyte network is established and is undergoing maturation. The authors shall also evaluate the impacts of LIF on P3 WT mouse retinas and see if LIF inhibition of retinal angiogenesis are observed as in the studies of LIFKO, LIF-overexpressing mice, as well as in APJ KO mice, before drawing the conclusion that the distinct roles of LIF in retinal angiogenesis is due to the different developmental stages.

We agree that these are important points. We recognize that there is not enough evidence to conclusively state that the distinct roles of LIF in neonatal mouse are due to the different developmental stages. We have toned down this statement in the results section. Studies with a constitutive transgenic LIF overexpression system may be difficult to interpret in view of the bell-shaped dose response of this cytokine and possibly other factors.

As recommended by the reviewer, we injected LIF intravitreally into P3 mice. In our experiments, LIF neither promoted nor inhibited retinal angiogenesis at P5, the time point examined by Sakimoto et al. (PMID: 22357924). However, we observed an increase in retinal angiogenesis at P8 (Fig. S10).

It is difficult to compare the study by Sakimoto et al., which examined LIFKO and APJ KO mice, known to have abnormalities in the retinal vasculature, with our experiments in wild-type mice. In addition, Sakimoto et al. tested a single dose of LIF, 500 ng. Instead, we tested multiple doses. While 50 ng LIF induced angiogenesis at P8, the 500ng dose had no growth promoting effects, consistent with the bell-shaped dose response of this cytokine.

Another possible explanation for the different results could be in the reagents employed. Sakimoto et al. reported in the "Methods" that they used LIF from ESGRO-Chemicon. There is no claim in the manufacturer's data sheet that this reagent is suitable for in vivo studies. The LPS level is not even indicated. The recommended use is for embryonic stem cell culture. To replicate the experiment by Sakimoto et al., we used a LIF preparation with very low LPS levels (Fig. S10).

As noted, it is possible that differences in experimental model systems, dosing regimens and probably also in the quality/purity of the reagents likely underlie the conflicting reports in the literature. We have made these points in the discussion.

3. Figure 5: please include the n number for Fig 5A. As there is usually a big variation in the laser-

induced CNV model (PMID: 24136346), please include at least 6 mice per group in the study. Both genders shall be used. The graph is recommended to be shown in dot format instead of bar format.

For all in vivo experiments in this manuscript, we used at least 10 mice per group to ensure a large enough sample size. As recommended, data on both genders are included. We repeated the CNV model with both genders and obtained similar results We replaced Fig. 5A with the new images. In addition, we replaced all quantification graphs to a dot format, as requested.

4. Figure S10, LIF was used at 0, 10, 100, and 1000ng. However, the authors stated that "In the NaIO3 model, LIF effects were dose-dependent from 10ng and the effect was saturated at 50ng" in the Result session. The authors please confirm.

We apologize for the confusion. We performed a dose response study with NaIO3 at 0, 10, 50, 100, 500, and 1000 ng. The statement "In the NaIO3 model, LIF effects were dose-dependent from 10ng and the effect was saturated at 50 ng" is correct. We replaced Figure S15 with the full dose range.

5. Although the current study focused on LIF's angiogenic impacts, LIF has also been reported to change visual cycle genes and proteins in retina and RPE (PMID: 22645143). Would this be a concern when use LIF to protect choroidal vessels? The authors please comment.

The authors of PMID: 22645143 tested higher doses than those we tested in our study (up to 3ug) in order to elicit the noted effects. Importantly, similar effects as LIF on visual cycle genes were reported with CNTF (PMID: 17192435). This cytokine was tested in GA patients and no evidence of detrimental effects was reported; in fact, a trend toward improved vision was noted (PMID: 21444807). Both LIF and CNTF have neurotrophic effects that in principle should be useful in treating AMD patients. However, unlike LIFR, CNTFR is not expressed in choroidal EC (single cell transcriptomics) and we hypothesize that this is one of the reasons why CNTF treatment did not slow-down GA progression.

6. As LIF has demonstrated inhibitory effects on BAE angiogenesis, would intravitreal LIF delivery lead to potential systemic side effects? There have been systemic concerns raised when use intraocular anti-VEGF therapy in retinal diseases. The authors please comment.

This is a valid point. However, anti-VEGF antibodies have a long systemic half-life, in the order of weeks. In contrast, LIF has a very short half-life. In the original *lif* knockout study, Stewart and colleagues (PMID 1522892) reported that the LIF $t_{1/2}$ in the systemic circulation is only 3-5 min. LIF injections could not rescue the failure of blastocyst implantation and only a continuous infusion with a micro-osmotic pump rescued this phenotype. This should alleviate concerns of systemic side effects, but further studies are needed. We mentioned the short half-life of LIF in the text.

<u>**Referee #3**</u> (Comments on Novelty/Model System for Author):

The medical relevance is high given that AMD still is the leading cause of blindness. The use of mouse models to study this is adequate.

Referee #3 (Remarks for Author):

N. Ferrara report interesting new findings about LIF acting on endothelial cells. They show that LIF

inhibits EC proliferation as reported before. In addition, they unraveled a new mechanism how LIF can induce endothelial cell death. In endothelial cells in the mouse retina, LIF however leads to the opposite effect stimulating endothelial proliferation and vessel growth. Importantly, this was associated with better outcome in several eye disease models in mice. In contrast to VEGF, LIF treatment did not lead to vessel leakage. While the findings or very interesting (e.g. organ-specific endothelial cell functions) and have translational potential, this study needs substantial revision.

Thank you for your comments

Major points:

The authors should determine absolute concentrations of LIF in the conditioned medium from LN-229 cells.

We have indicated the LIF concentrations by ELISA in the LN229 conditioned medium in the "Results". The value was 1.5 ng/ml.

The strong differences in endothelial cell response to LIF is intriguing. This study would gain substantial impact if the authors could show this not only in BCE and BAE cells but also in endothelial cells derived from other organs.

We tested human endothelial cells from several organs: skin, lung, choroid, and liver. Interestingly, we found that while LIF can promote mitogenesis in human choroidal and liver sinusoidal endothelial cells, it inhibits proliferation of dermal microvascular endothelial cells (Fig. S2C and D, Fig. S7A). In addition, qPCR analysis showed that, among the human endothelial cells tested, expression of Cathepsin L was upregulated only in dermal microvascular cells, at LIF concentrations that resulted in growth inhibition. These findings are consistent with our observations in bovine aortic EC (Fig. S7B).

Could it be that the differential endothelial cell response towards LIF is due to different expression of LIF receptors in organ-specific vascular beds? The authors may use of their RNAseq data sets to have a first look into this.

We re-analyzed single cell RNAseq data from *Tabula muris* and examined endothelial cells from different organs. The single-cell data set showed that, although the expression level of LIFR in liver sinusoid ECs was the highest, the level was similar among ECs from most of other organs (Fig. S16A). Therefore, the expression level may not be the only factor that determine whether promoting or inhibiting EC growth. Indeed, a conclusion of our study is that EC type-specific STAT3 signaling, in particular whether a mitogenic or pro-apoptotic pathway is triggered, is a major determinant of the LIF effects on EC.

In addition, the authors could block LIF receptors to determine whether these molecules indeed transmit the observed effects.

To address this question, we used siRNA to knock-down LIFR in BCE and BAE cells and then carried out proliferation assays. With 90% LIFR knockdown, the mitogenic effects of LIF on BCE were virtually abolished and so were the inhibitory effects on BAE cells (Fig. S3). These findings confirm that LIFR mediates both LIF pro- and anti-mitogenic effect in EC.

Moreover, single cell RNAseq data bases of organ-specific endothelial cells should be used to determine LIF receptor expression patterns (as already done partially by the authors suppl Table 4).

Thanks for the comment. We have provided a Figure based on analysis of *Tabula muris*, illustrating LIFR/LIF expression only in endothelial cells from different organs (Fig. S16). Please see our response to the reviewer' earlier point.

Does LIF affect in particular fenestrated capillary endothelial cells?

According to available single cell RNAseq data, LIFR expression is particularly high in liver sinusoid EC (LSEC) (and other fenestrated EC such as those in choroid and choroid plexus (SFig16). We carried out proliferation assays in human LSEC and found that LIF indeed promoted their proliferation (Fig. S2D). Besides LSEC, we tested four other human microvascular endothelial cells from different organs. LIF promoted growth of EC from choroid, while it had inhibitory effects on dermal EC (Fig. S7A). Bases on these findings, it may be difficult to conclude that LIF affects only fenestrated ECs.

The study lacks explanation why 10 to 100 ng LIF was injected into mouse eyes. What is the rationale for using this dose?

We employed three different mouse models in this manuscript: retinal angiogenesis, laser-induced CNV model and sodium iodate induced oxidative injury model. For each model, we have tested different doses of LIF and the effective concentration were slightly different among different models. We make a table here to clarify the working dose of LIF in each model (see below) and we reorganized the dose dependent results in (Fig. S15). As shown in the table, the optimal dose range of LIF in the retinal angiogenesis model was 10 - 50 ng, in the CNV model it was 10 – 50 ng and in sodium iodate model is 50-100 ng. It seems to us that these differences are within expected variability ranges. To be consistent among models, we injected 50 ng LIF if only one dose was used.

Regarding the permeability assay, since we injected VEGF at the dose of 100 ng per eye (which is commonly used according to the literature), we also used 100 ng LIF (nearly equimolar) to compare the permeability properties.

The in vitro experiments indicate that LIF acts directly on endothelial cells. The authors however also show LIF receptor expression in other cell types within the retina. This raises the question whether the observed effects upon intravitreal LIF injection are indeed mediated through LIF receptors on endothelial cells or by an unknown paracrine factor released from e.g., pericytes. Although this issue is difficult to address (cell type specific LIFR knockout mice would be ideal) the authors could try to stain tissue for LIF downstream signaling.

This is an excellent point. To address it, we performed immuno-fluorescent staining of phosphor-stat3 on retinal sections. Two hours after LIF injection, there was a clear increase in STAT3 phosphorylation in endothelial cells, based on CD31 IHC. The rapidity of this response argues for a direct effect of LIF on endothelial cells, rather than a paracrine mechanism. Cell-type specific conditional LIFR inactivation should be very informative to further address these questions. This is the current phase of our project, but these studies are expected to be time consuming. Besides endothelial cells, as expected, there was increased STAT3 phosphorylation also in the retinal ganglion cell layer (Fig. 4E).

An important information lacking in this study is whether the pro-angiogenic effect of LIF in the eye is transient or durable.

We agree that this is an important question. However, there is evidence that, in the absence of injury or other pathophysiological stimulus, newly formed blood vessels in response to the application of a defined angiogenic factor such as VEGF tend to rapidly regress, especially in some tissues (PMID: 10653008). With these caveats, we intravitreally injected LIF at day 1 and then at day 7. Our data show that there was a measurable growth promoting effect of LIF even at day 14 (Fig. S9B). Future studies are needed to develop a delivery system that can ensure steady LIF concentrations.

In addition, are the new blood vessels covered by pericytes in a similar degree as those in the control group?

We performed a flat mount co-staining of CD31 and NG2 in LIF-treated retina. The results show that the percentage of vessels with NG2 signal in LIF-treated retina is similar to that in PBS group, (Fig. S9C).

The authors show a very interesting kind of cell death upon LIF treatment in BAE cells. However, it remained unclear whether this is only observed in this particular cell type under cell culture conditions or whether this would also occur in mice. This is also an important question when thinking about translation into clinical application.

Although cathepsin-mediated cell death has not been previously reported in response to LIF, it has been implicated in STAT3-dependent pathophysiological processes, for example post-lactation involution of mammary gland (PMID21336304). We show in this revision that LIF results in cathepsin upregulation also in human dermal microvascular EC, at concentrations that are strongly correlated with growth inhibition (Fig. S7B). Furthermore, we show that OSM, another member of the IL-6 family, inhibits retinal angiogenesis and has detrimental effects in the Na Iodate model (Fig5, A, B). Fig E). In contrast to LIF, OSM injection upregulated cathepsin expression in the retina (Fig. S12 C-E). The data suggest that cathepsin upregulation could be a biomarker for detrimental/proapoptotic effects of LIF and other member of the IL-6 family, that could guide patient selection and dosing regimens. We have mentioned these points in the Discussion.

Several figures should be improved. For example, Fig. 7 has very low resolution making it difficult to read. Also p values should be indicated for all statistical analyses.

Thank you for the comment. We improved the resolution of the figures and indicated the p value in each graph.

7th Oct 2021

Dear Dr. Ferrara,

Thank you for the submission of your revised manuscript to EMBO Molecular Medicine. As you will see from the reports below, the three referees are satisfied with the revisions and support publication of the manuscript. I am therefore pleased to inform you that we will be able to accept your manuscript once the following minor editorial points will be addressed:

1/ Main manuscript text:
- During our standard cross-check analysis, text similarities with previously published materials from your lab were found in the introduction (see screenshots attached), therefore please slightly modify these paragraphs.
- We can accommodate a maximum of 5 keywords, please adjust accordingly.
- Please remove the highlights in the text, accept the changes, and only keep in track changes mode any new modification.
- Material and methods:
o Cells: please indicate whether the cells were authenticated (when applicable) and tested for mycoplasma contamination.
o Mice: please indicate the origin of the mice, as well as their housing and husbandry conditions. Make sure the gender of the mice is indicated for all experiments.
- Data Availability section: RNAseq raw data generated in this study need to be deposited in an appropriate public database, and the accession numbers and database listed under 'Data Availability'. The text you have currently under this section should be removed.
- Conflict of Interest: Thank you for providing a statement, please link it to an (or several) author (s).

2/ Appendix:
- Please provide exact p values for the appendix figures as well (including for ns, non-significant)
- Please indicate the size of the error bar for Fig. S11B.

3/ Checklist:
- section C/7: please indicate whether the cells were tested for mycoplasma contamination.
- section D/10: please confirm compliance with ARRIVE guidelines.
- section F/18-19: please include the deposition number and URL for deposited RNAseq data generated in this study.

4/ We would also encourage you to include the source data for figure panels that show essential data. Numerical data should be provided as individual .xls or .csv files (including a tab describing the data). For blots or microscopy, uncropped images should be submitted (using a zip archive if multiple images need to be supplied for one panel). Additional information on source data and instruction on how to label the files are available at
.

Regarding the data you already shared with us (for Figure S13A), we would suggest to include the link to the main manuscript text.

5/ Please provide a "Paper Explained" section: EMBO Molecular Medicine articles are accompanied by a summary of the articles to emphasize the major findings in the paper and their medical implications for the non-specialist reader. Please provide a draft summary of your article highlighting:
- the medical issue you are addressing,
- the results obtained and
- their clinical impact.
This may be edited to ensure that readers understand the significance and context of the research. Please refer to any of our published articles for an example.

6/ Please provide a synopsis text and figure. Synopses are displayed on the journal webpage and are freely accessible to all readers. They include a short stand first (maximum of 300 characters, including space) as well as 2-5 one-sentences bullet points that summarizes the paper. Please write the bullet points to summarize the key NEW findings. They should be designed to be complementary to the abstract - i.e. not repeat the same text. We encourage inclusion of key acronyms and quantitative information (maximum of 30 words / bullet point). Please use the passive voice.
Please also suggest a striking image or visual abstract to illustrate your article as a TIFF/PNG file 550 px wide x 300-600 px high.
Please attach these in a separate file or send them by email, we will incorporate them accordingly.

7/ Please enter the funding information in the submission system

8/ As part of the EMBO Publications transparent editorial process initiative (see our Editorial at

http://embomolmed.embopress.org/content/2/9/329), EMBO Molecular Medicine will publish online a Review Process File (RPF) to accompany accepted manuscripts.
This file will be published in conjunction with your paper and will include the anonymous referee reports, your point-by-point response and all pertinent correspondence relating to the manuscript. Let us know whether you agree with the publication of the RPF and as here, if you want to remove or not any figures from it prior to publication.
Please note that the Authors checklist will be published at the end of the RPF.

I look forward to receiving your revised manuscript.

With kind regards,

Lise Roth

Lise Roth, PhD
Editor
EMBO Molecular Medicine

***** Reviewer's comments *****

Referee #1 (Remarks for Author):

The authors have addressed all my concerns including toning down conclusions in results. I have no further requests.

Referee #2 (Comments on Novelty/Model System for Author):

The model system was appropriate.

Referee #2 (Remarks for Author):

The authors have addressed all my concerns.

Referee #3 (Remarks for Author):

The authors have addressed all of my concerns. With this the manuscript appears ready for publication.

The authors performed the requested editorial changes.

25th Oct 2021

Dear Dr. Ferrara,

Thank you for depositing the RNAseq data in a public repository. I am very pleased to inform you that your manuscript is now accepted for publication and will be sent to our publisher to be included in the next available issue of EMBO Molecular Medicine!

Congratulations on your interesting work,

With kind regards,

Lise Roth

Lise Roth, Ph.D
Editor
EMBO Molecular Medicine

Follow us on Twitter @EmboMolMed
Sign up for eTOCs at embopress.org/alertsfeeds

EMBO PRESS

YOU MUST COMPLETE ALL CELLS WITH A PINK BACKGROUND ➔

PLEASE NOTE THAT THIS CHECKLIST WILL BE PUBLISHED ALONGSIDE YOUR PAPER

Corresponding Author Name: Napoleone Ferrara
Journal Submitted to: EMBO Molecular Medicine
Manuscript Number: EMM-2021-14511

Reporting Checklist For Life Sciences Articles (Rev. June 2017)

This checklist is used to ensure good reporting standards and to improve the reproducibility of published results. These guidelines are consistent with the Principles and Guidelines for Reporting Preclinical Research issued by the NIH in 2014. Please follow the journal's authorship guidelines in preparing your manuscript.

A- Figures
1. Data
The data shown in figures should satisfy the following conditions:

- ➔ the data were obtained and processed according to the field's best practice and are presented to reflect the results of the experiments in an accurate and unbiased manner.
- ➔ figure panels include only data points, measurements or observations that can be compared to each other in a scientifically meaningful way.
- ➔ graphs include clearly labeled error bars for independent experiments and sample sizes. Unless justified, error bars should not be shown for technical replicates.
- ➔ if n< 5, the individual data points from each experiment should be plotted and any statistical test employed should be justified
- ➔ Source Data should be included to report the data underlying graphs. Please follow the guidelines set out in the author ship guidelines on Data Presentation.

2. Captions

Each figure caption should contain the following information, for each panel where they are relevant:

- ➔ a specification of the experimental system investigated (eg cell line, species name).
- ➔ the assay(s) and method(s) used to carry out the reported observations and measurements
- ➔ an explicit mention of the biological and chemical entity(ies) that are being measured.
- ➔ an explicit mention of the biological and chemical entity(ies) that are altered/varied/perturbed in a controlled manner.

- ➔ the exact sample size (n) for each experimental group/condition, given as a number, not a range;
- ➔ a description of the sample collection allowing the reader to understand whether the samples represent technical or biological replicates (including how many animals, litters, cultures, etc.).
- ➔ a statement of how many times the experiment shown was independently replicated in the laboratory.
- ➔ definitions of statistical methods and measures:
  - common tests, such as t-test (please specify whether paired vs. unpaired), simple $\chi 2$ tests, Wilcoxon and Mann-Whitney tests, can be unambiguously identified by name only, but more complex techniques should be described in the methods section;
  - are tests one-sided or two-sided?
  - are there adjustments for multiple comparisons?
  - exact statistical test results, e.g., P values = x but not P values < x;
  - definition of 'center values' as median or average;
  - definition of error bars as s.d. or s.e.m.

Any descriptions too long for the figure legend should be included in the methods section and/or with the source data.

In the pink boxes below, please ensure that the answers to the following questions are reported in the manuscript itself. Every question should be answered. If the question is not relevant to your research, please write NA (non applicable). We encourage you to include a specific subsection in the methods section for statistics, reagents, animal models and human subjects.

USEFUL LINKS FOR COMPLETING THIS FORM

http://www.antibodypedia.com
http://1degreebio.org
http://www.equator-network.org/reporting-guidelines/improving-bioscience-research-repor
http://grants.nih.gov/grants/olaw/olaw.htm
http://www.mrc.ac.uk/Ourresearch/Ethicsresearchguidance/Useofanimals/index.htm
http://ClinicalTrials.gov
http://www.consort-statement.org
http://www.consort-statement.org/checklists/view/32-consort/66-title
http://www.equator-network.org/reporting-guidelines/reporting-recommendations-for-tumo
http://datadryad.org
http://figshare.com
http://www.ncbi.nlm.nih.gov/gap
http://www.ebi.ac.uk/ega
http://biomodels.net/
http://biomodels.net/miriam/
http://jjj.biochem.sun.ac.za
https://osp.od.nih.gov/biosafety-biosecurity-and-emerging-biotechnology/
http://www.selectagents.gov/

B- Statistics and general methods

Please fill out these boxes ➔ (Do not worry if you cannot see all your text once you press return)

1.a. How was the sample size chosen to ensure adequate power to detect a pre-specified effect size?	Sample size was chosen according to previous literature.
1.b. For animal studies, include a statement about sample size estimate even if no statistical methods were used.	Yes, we included the sample size in each figure legends
2. Describe inclusion/exclusion criteria if samples or animals were excluded from the analysis. Were the criteria pre-established?	We included all the samples for analysis.
3. Were any steps taken to minimize the effects of subjective bias when allocating animals/samples to treatment (e.g. randomization procedure)? If yes, please describe.	Mice were allocated randomlly and blindly to different groups by another person.
For animal studies, include a statement about randomization even if no randomization was used.	We included the statement in "Method" section.
4.a. Were any steps taken to minimize the effects of subjective bias during group allocation or/and when assessing results (e.g. blinding of the investigator)? If yes please describe.	Mice were blindly allocated and quantification were done by two different investigators.
4.b. For animal studies, include a statement about blinding even if no blinding was done	We included the statement in "Method" section.
5. For every figure, are statistical tests justified as appropriate?	Yes.
Do the data meet the assumptions of the tests (e.g., normal distribution)? Describe any methods used to assess it.	Yes, data were normal distributed and assessed by Shapiro–Wilk test.
Is there an estimate of variation within each group of data?	The estimate of variation was carried out by Graphpad ANOVA analysis function.

Is the variance similar between the groups that are being statistically compared?	Yes.

C- Reagents

6. To show that antibodies were profiled for use in the system under study (assay and species), provide a citation, catalog number and/or clone number, supplementary information or reference to an antibody validation profile. e.g., Antibodypedia (see link list at top right), 1DegreeBio (see link list at top right).	Yes, we included the catalog number of each antibodies we used.
7. Identify the source of cell lines and report if they were recently authenticated (e.g., by STR profiling) and tested for mycoplasma contamination.	Our cell lines are all commercially available and were provided with authentication files and are tested to be mycoplasma-free.

* for all hyperlinks, please see the table at the top right of the document

D- Animal Models

8. Report species, strain, gender, age of animals and genetic modification status where applicable. Please detail housing and husbandry conditions and the source of animals.	Yes, we included the detail information of mice we used.
9. For experiments involving live vertebrates, include a statement of compliance with ethical regulations and identify the committee(s) approving the experiments.	Yes, we inclued the ethical regulations and committee approving the experiments in "Methods" section.
10. We recommend consulting the ARRIVE guidelines (see link list at top right) (PLoS Biol. 8(6), e1000412, 2010) to ensure that other relevant aspects of animal studies are adequately reported. See author guidelines, under 'Reporting Guidelines'. See also: NIH (see link list at top right) and MRC (see link list at top right) recommendations. Please confirm compliance.	All animal studies were under compliance with ARRIVE guidelines.

E- Human Subjects

11. Identify the committee(s) approving the study protocol.	NA
12. Include a statement confirming that informed consent was obtained from all subjects and that the experiments conformed to the principles set out in the WMA Declaration of Helsinki and the Department of Health and Human Services Belmont Report.	NA
13. For publication of patient photos, include a statement confirming that consent to publish was obtained.	NA
14. Report any restrictions on the availability (and/or on the use) of human data or samples.	NNA
15. Report the clinical trial registration number (at ClinicalTrials.gov or equivalent), where applicable.	NA
16. For phase II and III randomized controlled trials, please refer to the CONSORT flow diagram (see link list at top right) and submit the CONSORT checklist (see link list at top right) with your submission. See author guidelines, under 'Reporting Guidelines'. Please confirm you have submitted this list.	NA
17. For tumor marker prognostic studies, we recommend that you follow the REMARK reporting guidelines (see link list at top right). See author guidelines, under 'Reporting Guidelines'. Please confirm you have followed these guidelines.	NA

F- Data Accessibility

18: Provide a "Data Availability" section at the end of the Materials & Methods, listing the accession codes for data generated in this study and deposited in a public database (e.g. RNA-Seq data: Gene Expression Omnibus GSE39462, Proteomics data: PRIDE PXD000208 etc.) Please refer to our author guidelines for 'Data Deposition'.<br><br>Data deposition in a public repository is mandatory for:<br>a. Protein, DNA and RNA sequences<br>b. Macromolecular structures<br>c. Crystallographic data for small molecules<br>d. Functional genomics data<br>e. Proteomics and molecular interactions	The RNA sequencing datasets in this study are available on NCBI Gene Expression Omnibus with the accession number GSE186481 (https://www.ncbi.nlm.nih.gov/geo/query/acc.cgi?acc=GSE186481).
19. Deposition is strongly recommended for any datasets that are central and integral to the study; please consider the journal's data policy. If no structured public repository exists for a given data type, we encourage the provision of datasets in the manuscript as a Supplementary Document (see author guidelines under 'Expanded View' or in unstructured repositories such as Dryad (see link list at top right) or Figshare (see link list at top right).	NA
20. Access to human clinical and genomic datasets should be provided with as few restrictions as possible while respecting ethical obligations to the patients and relevant medical and legal issues. If practically possible and compatible with the individual consent agreement used in the study, such data should be deposited in one of the major public access-controlled repositories such as dbGAP (see link list at top right) or EGA (see link list at top right).	NA
21. Computational models that are central and integral to a study should be shared without restrictions and provided in a machine-readable form. The relevant accession numbers or links should be provided. When possible, standardized format (SBML, CellML) should be used instead of scripts (e.g. MATLAB). Authors are strongly encouraged to follow the MIRIAM guidelines (see link list at top right) and deposit their model in a public database such as Biomodels (see link list at top right) or JWS Online (see link list at top right). If computer source code is provided with the paper, it should be deposited in a public repository or included in supplementary information.	NA

G- Dual use research of concern

22. Could your study fall under dual use research restrictions? Please check biosecurity documents (see link list at top right) and list of select agents and toxins (APHIS/CDC) (see link list at top right). According to our biosecurity guidelines, provide a statement only if it could.	No.
